# How marine are Marine Stramenopiles (MAST)? A cross-system evaluation

**DOI:** 10.1093/femsec/fiae130

**Published:** 2024-10-07

**Authors:** Aleix Obiol, Javier del Campo, Colomban de Vargas, Frédéric Mahé, Ramon Massana

**Affiliations:** Department of Marine Biology and Oceanography, Institut de Ciències del Mar (ICM-CSIC), Barcelona, Catalonia, Spain; Biodiversity Program, Institut de Biologia Evolutiva (CSIC-Universitat Pompeu Fabra), Barcelona, Catalonia, Spain; Sorbonne Université, CNRS, Station Biologique de Roscoff, UMR7144, ECOMAP, Roscoff, France; Research Federation for the study of Global Ocean Systems Ecology and Evolution, FR2022/Tara GOSEE, Paris, France; CIRAD, UMR PHIM, F-34398 Montpellier, France; PHIM, Univ Montpellier, CIRAD, INRAE, Institut Agro, IRD, Montpellier, France; Department of Marine Biology and Oceanography, Institut de Ciències del Mar (ICM-CSIC), Barcelona, Catalonia, Spain

**Keywords:** biogeography, diversity and distribution, habitat, Marine Stramenopiles, microbial ecology

## Abstract

Marine Stramenopiles (MAST) were first described two decades ago through ribosomal RNA gene (rRNA gene) sequences from marine surveys of microbial eukaryotes. MAST comprise several independent lineages at the base of the Stramenopiles. Despite their prevalence in the ocean, the majority of MAST diversity remains uncultured. Previous studies, mainly in marine environments, have explored MAST’s cell morphology, distribution, trophic strategies, and genomics using culturing-independent methods. In comparison, less is known about their presence outside marine habitats. Here, we analyse the extensive EukBank dataset to assess the extent to which MAST can be considered marine protists. Additionally, by incorporating newly available rRNA gene sequences, we update Stramenopiles phylogeny, identifying three novel MAST lineages. Our results indicate that MAST are primarily marine with notable exceptions within MAST-2 and MAST-12, where certain subclades are prevalent in freshwater and soil habitats. In the marine water column, only a few MAST species, particularly within clades -1, -3, -4, and -7, dominate and exhibit clear latitudinal distribution patterns. Overall, the massive sequencing dataset analysed in our study confirms and partially expands the previously described diversity of MASTs groups and underscores the predominantly marine nature of most of these uncultured lineages.

## Introduction

Microbial and unicellular forms harbour the vast majority of organismal diversity within eukaryotes (Burki et al. 2020) and populate all habitats on Earth. Through their activity, microbial eukaryotes play essential roles in global biogeochemical cycles as primary producers, grazers, parasites, and decomposers. Molecular techniques applied to environmental surveys began to unravel the phylogenetic diversity of microbial eukaryotes. It was in the early 2000s, when a largely unknown diversity within microbial eukaryotes was identified (Díez et al. 2001, López-García et al. 2001, Moon-Van Der Staay et al. 2001) and new taxonomic groups were described (Massana et al. 2004, Not et al. 2007, Guillou et al. 2008). Among these new taxa are Marine Stramenopiles (MAST), from which 18 different lineages scattered across the non-Ochrophyta phylogenetic space are currently defined (Massana et al. 2014). MAST are widespread in the ocean (Obiol et al. 2021), and just three lineages account for about 20% of cells within heterotrophic flagellates assemblages (Mangot et al. 2018). Although less data exists in nonmarine environments, some MAST clades have also been detected in freshwater and soils surveys (Massana et al. 2014, Simon et al. 2015, Metz et al. 2022a, Singer et al. 2021). MAST remain largely uncultured, with a few exceptions within MAST-3 (Cavalier-Smith and Scoble 2013) and MAST-6 (Shiratori et al. 2017, Cho et al. 2023). Despite being generally considered heterotrophs, little is known about the functional roles of these groups. Thus far, there exists microscopic evidence of phagotrophic feeding within MAST-1, -2, -3, -4, -6, and -7 (Massana et al. 2009, Piwosz and Pernthaler 2010, Cavalier-Smith and Scoble 2013, Piwosz et al. 2013, Rodríguez-Martínez et al. 2022) and of parasitism within some MAST-3 species (Gómez et al. 2011). More recently, genomic data obtained through single-cell genomics is proving invaluable in furthering our understanding of MAST cell biology and ecology (Seeleuthner et al. 2018, Labarre et al. 2021, Latorre et al. 2021).

It was 10 years ago when the last reevaluation of MAST diversity was conducted (Massana et al. 2014). At that time, ribosomal RNA (rRNA) gene sequences derived mostly from clone libraries, a tedious approach that retrieves up to hundreds of sequences per sample. Subsequent studies have yielded additional nearly complete rRNA gene sequences, which could be used in updated phylogenetic trees to reevaluate the existing clades and potentially identify new ones. However, the most critical change during the last 10 years has been the emergence of high-throughput sequencing platforms. These have dramatically increased sequencing depths while reducing costs (Goodwin et al. 2016), making metabarcoding routine to survey microbial diversity (Burki et al. 2021). This technological revolution has facilitated the collection and sequencing of thousands of samples at both global (Acinas et al. 2022) and regional scales (Giner et al. 2019, Ollison et al. 2021), each sample with thousands of sequences, leading to the public release of extensive datasets. Most of these studies used partial regions of the 18S rRNA gene, typically the V4 or V9 regions, but recent efforts with longer amplicons are also becoming successful (Jamy et al. 2020, 2022). The collaborative initiatives to gather and analyse all available metabarcoding data from microbial eukaryotes have resulted in the development of tools such as metaPR^2^ (Vaulot et al. 2022) and consortia like EukBank (Berney et al. 2023). These advancements now enable the reevaluation of MAST diversity and distribution at a global scale and across marine and terrestrial habitats with unprecedented resolution.

In this study, we evaluate the distribution of MAST across marine, freshwater, and soil habitats using the EukBank dataset. This dataset encompasses information derived from the V4 region of the 18S rRNA gene, offering the most comprehensive compilation of global metabarcoding data to date. The overall aim of our study is to update the standing framework for MAST diversity and distribution reported 10 years ago (Massana et al. 2014). We hypothesize that the substantial increase in both sequencing effort and sample diversity, including previously unexplored habitats, would significantly expand our current understanding of MAST diversity. Thus, we conducted our analyses to answer the following specific questions: (1) Is there still undescribed diversity within MAST? (2) What are the patterns of MAST distribution at the clade, subclade, and sequence levels within and across different habitats? (3) Are there any exclusively terrestrial MAST taxa?

## Materials and methods

### Building a phylogenetic reference of 18S rRNA gene for Stramenopiles

We built a reference phylogenetic tree of Stramenopiles with the small subunit rRNA gene (18S rRNA gene). As we focused on the non-Ochrophyta region of the Stramenopiles where MAST are placed, we downloaded non-Ochrophyta sequences from PR^2^ version 5.0.0 (Guillou et al. 2013). We removed sequences shorter than 1100 bp and longer than 2000 bp that did not belong to MAST and clustered the remaining sequences at 99% identity using command *–cluster_fast* from VSEARCH version 2.18.0 (Rognes et al. 2016). We aligned them using MAFFT version 7.402 (Katoh and Standley 2013) with *-auto* option and trimmed long tails surpassing the 18S region in some sequences. We mapped the sequences against eukaryotesV4 version 8 database (Obiol et al. 2020) using BLAST version 2.7.1 to simplify taxonomic labels. To detect additional sequences not deposited in PR^2^, we searched in GenBank using as queries the EukBank Stramenopile ASVs and identified nine sequences >800 bp that represented additional diversity. We clustered the resulting non-Ochrophyta dataset at 97% identity with VSEARCH and combined it with Ochrophyta sequences (97% clustered) from Massana et al. (2014), as well as 19 outgroup sequences. We aligned them using MAFFT with G-INS-i method and built a tree with IQ-TREE version 2.0.6 (Nguyen et al. 2015) using model GTR+F+R10 with 1000 replicates using ultrafast bootstrap (UFBoot) and Shimodaira–Hasegawa approximate likelihood-ratio test (SH-aLRT) (Guindon et al. 2010, Hoang et al. 2018). We manually inspected long branches in the resulting tree, which were often chimeras; we removed them and started a new alignment and a new tree. We iterated this process five times, until we did not detect new chimeric sequences. The final reference tree contained 1120 Stramenopiles sequences (median length 1653 bp) and we used it to validate existing MAST clades and identify new ones. To evaluate the internal topology of each MAST clade, we built separate trees per clade using all available sequences in PR^2^ plus the newly obtained GenBank sequences. We built these trees following the same process as for the general reference tree. To define MAST-6 subclades, we used additional data from Cho et al. (2023).

### The non-Ochrophyta Stramenopiles from the EukBank dataset

The EukBank global dataset is a compilation of metabarcoding projects targeting the V4 region of the 18S rRNA gene. It comprises 12 672 samples collected from terrestrial and marine habitats. A detailed explanation on how this dataset was built can be found in Berney et al. (2023). Briefly, raw sequences were downloaded from EMBL/EBI-ENA, paired-end reads were merged with VSEARCH’s *–fastq_mergepairs* command and then trimmed with cutadapt to remove primers and adapters. Reads were dereplicated using VSEARCH’s command -*derep_fulllength* and amplicon sequence variants (ASVs) were obtained using Swarm (Mahé et al. 2021). Chimeras were removed with VSEARCH’s command -*uchime_denovo* and low-quality sequences were discarded. Taxonomic assignment was performed using the EukRibo version 1 database (Berney et al. 2022).

To build the EukBank dataset of non-Ochrophyta Stramenopiles, we removed 2083 samples that were sequenced with Roche 454, had missing or wrong coordinates (e.g. marine samples with coordinates pointing to land), came from organisms (microbiomes), or belonged to ‘land_water’ and ‘marine_ice’ habitats (as they comprised a low number of ill-defined samples). For water column samples (‘marine water’ and ‘freshwater’), we removed 894 samples in which the lower size fraction was larger than 10 µm. We kept 321 aquatic samples in which the size fraction was not mentioned, assuming that these contained at least the picoplanktonic fraction (0.2–3 µm). Next, we removed ASVs shorter than 300 bp and ASVs assigned to Metazoa and Streptophyta. After this filtering, we recalculated the total amount of reads per sample (used later to transform absolute counts to relative read abundances) and removed 1907 samples with less than 10 000 reads. We kept 7788 samples (belonging to 89 different surveys) for further analyses.

We then kept ASVs belonging to Stramenopiles based on the EukRibo taxonomy and found in at least five samples (16 999 ASVs). To retrieve ASVs of interest that were not initially classified as Stramenopiles, we performed a BLAST search of all EukBank ASVs using our Stramenopiles reference complemented with 63 ASVs coming from Obiol et al. (2021) that could belong to new MAST clades. We added to the dataset all ASVs that had a hit with at least 95% identity and a minimum coverage of 300 bp (878 ASVs from which 388 belonged to Ochrophyta). Then, we removed Ochrophyta ASVs (10 335 ASVs) from the dataset and refined the taxonomic assignment of the remaining ASVs by performing a phylogenetic placement to our Stramenopiles reference clustered at 99% identity. To do so, we added the ASVs to the reference alignment and phylogenetic tree using PaPaRa version 2.5 (Berger and Stamatakis 2011) and assigned taxonomy to them using EPA-ng version 0.3.8 (Barbera et al. 2019) coupled with gappa version 0.8.4 (Czech et al. 2020). We labelled as ‘InSedMAST’ (Incertae Sedis MAST) those ASVs without a clear taxonomic assignment. Furthermore, we built separate trees for each MAST clade by aligning ASVs assigned to a given clade to the aligned unclustered sequences used in Figure S1 and its corresponding phylogenetic tree by using PaPaRa coupled with IQ-TREE. The final non-Ochrophyta dataset contained 7542 phylogenetically assigned ASVs with an average length of 386 bp (12 bp standard deviation) representing 29 673 533 total reads.

### Data analyses

We performed all data analyses using R version 4.3.2 (R Core Team 2023) and the packages tidyverse version 2.0.0 (Wickham et al. 2019) and phyloseq version 1.44.0 (McMurdie and Holmes 2013) as implemented in speedyseq (McLaren 2023). We conducted ordination analyses using the package vegan version 2.6.4 (Oksanen et al. 2022) using the table of ASV counts for all Stramenopiles. First, we calculated the average of Bray–Curtis distances from 100 iterations computed with function avgdist() with a subsampling of 10 000 reads. Then, we performed a nonmetric multidimensional scaling (NMDS) with function metaMDS(). We conducted heatmap analyses with package ComplexHeatmap version 2.15.4 (Gu 2022).

## Results

### An updated definition of MAST clades within the phylogeny of Stramenopiles

**Figure 1.**
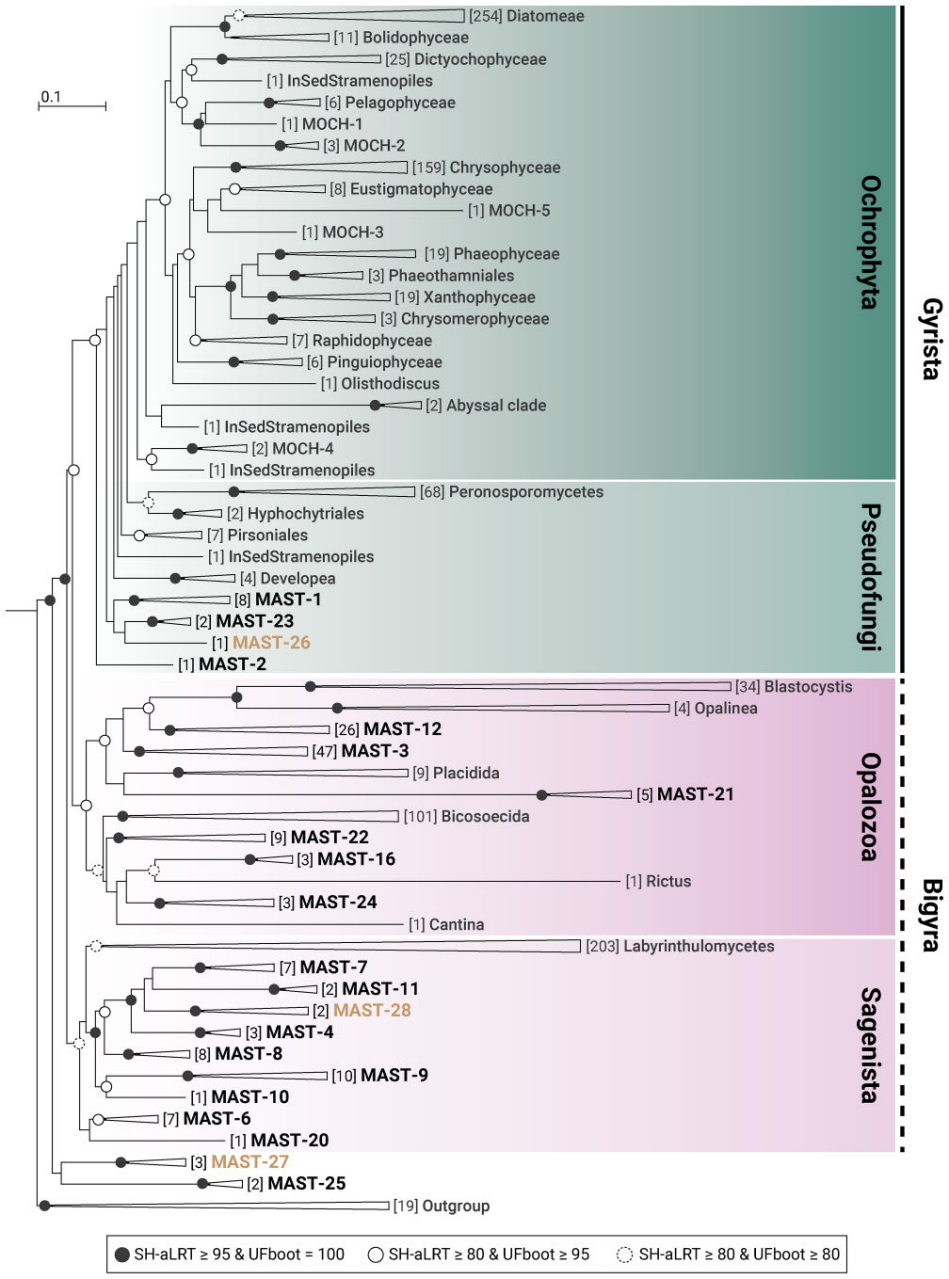
High-rank phylogeny of Stramenopiles based on almost complete 18S rRNA gene sequences. The tree was built in IQ-TREE with model GTR+F+R10 using 1120 Stramenopiles sequences and 19 outgroup sequences (clustered at 97% identity; 4671 sites). Support values were calculated with 1000 SH-aLRT and 1000 UFBoot replicates. These are shown in dots of different colours and outlines for the main groups. The number of sequences within each clade is denoted in brackets preceding their names. The new clades defined in this study are highlighted.

We built a reference tree of Stramenopiles using the currently available nearly complete 18S rRNA gene sequences clustered at 97% identity (Fig. 1). The topology was consistent with previous 18S rRNA gene phylogenies of Stramenopiles, with a highly diverse and monophyletic Ochrophyta, several lineages basal to Ochrophyta that form the loose group Pseudofungi (Ochrophyta plus Pseudofungi form the Gyrista), and two large groups basal to Gyrista formed by Sagenista and Opalozoa (which together form the loose group Bigyra). The 18 previously described MAST clades formed as expected (Fig. 1), and here we proposed three additional MAST lineages, one within Pseudofungi (MAST-26), one within Sagenista (MAST-28), and one at the very base of Stramenopiles (MAST-27). As compared with many of the previously defined clades, these new clades were formed by only two to three rRNA gene sequences (Table S1). No new MAST clades emerged within Opalozoa. When looking at the internal structure of each MAST clade we also confirmed the previously defined subclades and found a few additional ones within four clades (Figure S1). We defined new subclades in MAST-3 (M), MAST-8 (G), MAST-9 (E), and MAST-12 (F–H). Moreover, we divided MAST-6 into clades A–E (Figure S1). The final dataset included 21 MAST clades, which were divided into 60 subclades (Table 1; Table S1).

**Table 1.**
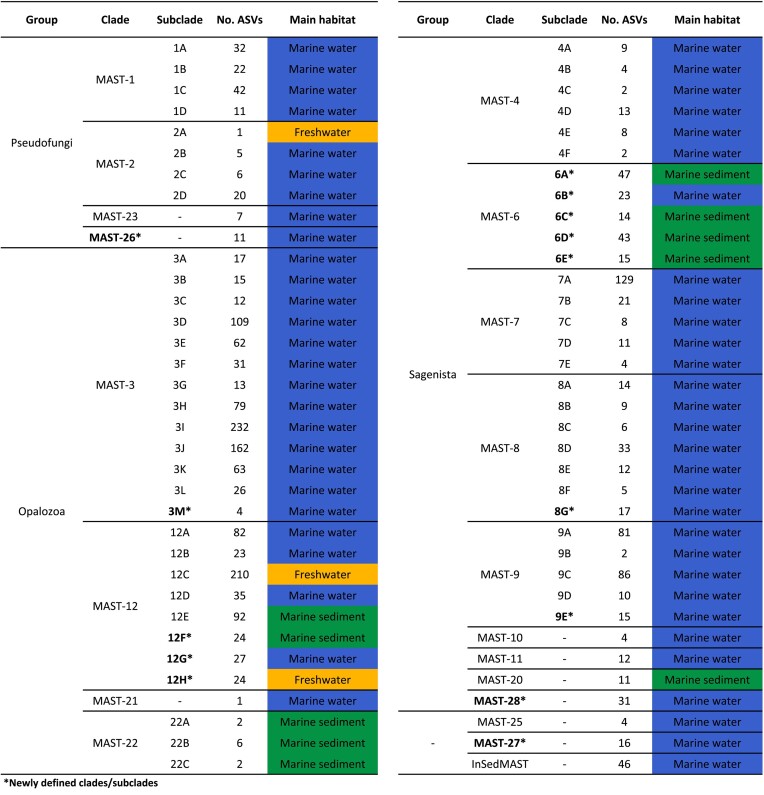
An overview of MAST diversity and distribution. General taxonomy, total number of ASVs, and preferred habitat (habitat where each group displays highest mean relative read abundance) for each MAST clade and subclade.

### Overview of Stramenopiles across the EukBank dataset

**Figure 2.**
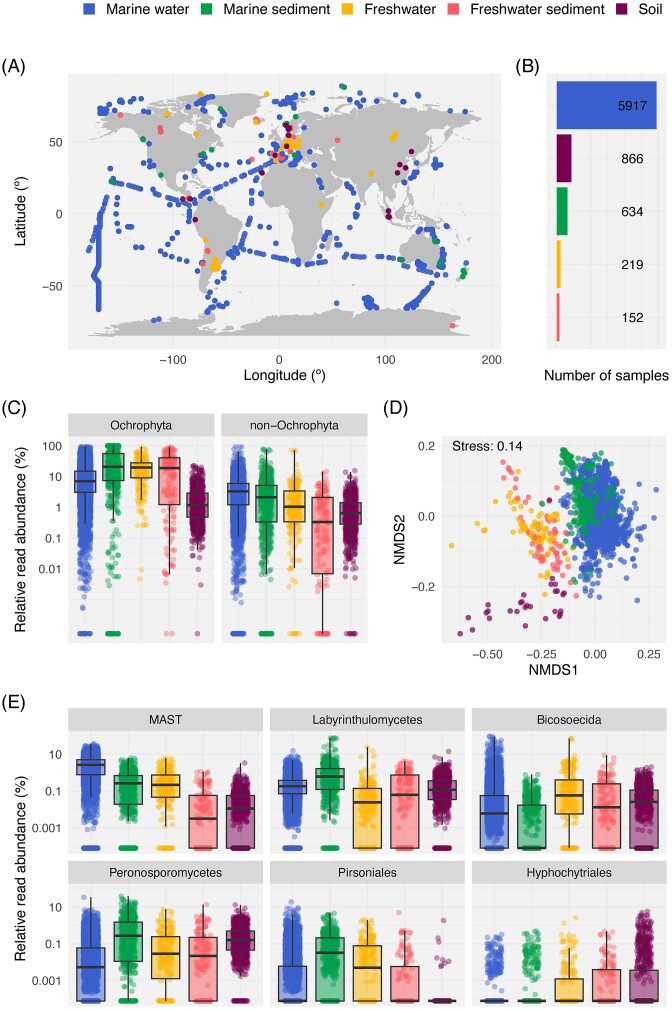
The EukBank dataset for Stramenopiles. (A) World map displaying the geographic distribution of samples coloured by the habitat they belong to. (B) Number of samples per habitat. (C) Relative read abundance of the Ochrophyta and non-Ochrophyta Stramenopiles in the five sampled habitats. (D) NMDS of samples based on Stramenopiles ASVs using Bray–Curtis dissimilarities. Only samples with at least 10 000 Stramenopile reads were considered for this analysis. (E) Relative read abundance of the main groups of Stramenopiles (excluding Ochrophyta) in the five sampled habitats. In figures (C) and (E), each dot represents the pooled relative read abundance of all ASVs belonging to a given group in a sample. Values are displayed in logarithmic scale using the lowest positive value in the dataset as a pseudocount. Groups are arranged by total mean relative read abundance. Boxes in plots represent the interquartile range (IQR), with the median line inside. Whiskers extend 1.5 times the IQR and individual points beyond this range represent outliers.

The dataset analysed in this study comprised 7788 samples across the globe (Table S2) that were divided into five different habitats: ‘marine water’, ‘marine sediment’, ‘freshwater’, ‘freshwater sediment’, and ‘soil’. Samples from ‘marine water’ clearly outnumbered the sampling of the rest of the habitats (Fig. 2A and B) and covered the majority of oceanic regions. ‘Marine sediment’ consisted mainly of coastal samplings from a depth of <200 m; most of the ‘freshwater’ and ‘freshwater sediment’ samples were collected in the northern hemisphere; and ‘soil’ samples were restricted to a few terrestrial regions (Fig. 2A). Within this dataset Stramenopiles averaged 16% of the overall protist signal in all habitats. Ochrophyta displayed high relative read abundances in marine and terrestrial habitats (Fig. 2C), with the highest values in marine sediment and freshwater, followed by freshwater sediment and marine water (Table S3). The non-Ochrophyta groups showed their highest read abundances in marine samples and had lower values in terrestrial ones (Fig. 2C). Overall, Ochrophyta displayed significantly higher relative read abundances than non-Ochrophyta groups in all sampled habitats (Table S3A). An ordination of all samples based on their content in Stramenopile ASVs revealed a drastic separation between marine and terrestrial samples (Fig. 2D; Table S4). Marine samples exhibited a partial division based on their origin (sediment or water column), but such differentiation was not observed in freshwater samples, where both the water column and sediments appeared together (Fig. 2D). Besides, a small number of sediment samples from both marine and freshwater habitats were grouped. Soil samples were markedly separated from the rest (Fig. 2D). Regarding the main non-Ochrophyta taxonomic groups, MAST presented a marked preference for marine water (Fig. 2E), where they accounted for more than 3% on average of the overall protist signal (Table S3B). Labyrinthulomycetes, Peronosporomycetes, and Pirsoniales had marine sediment as their preferred habitat (Fig. 2E), Bicosoecida had its highest abundance in freshwater samples, and Hyphochytriales exhibited low abundances in the five sampled habitats (Fig. 2E; Table S3B).

### MAST distribution across systems

**Figure 3.**
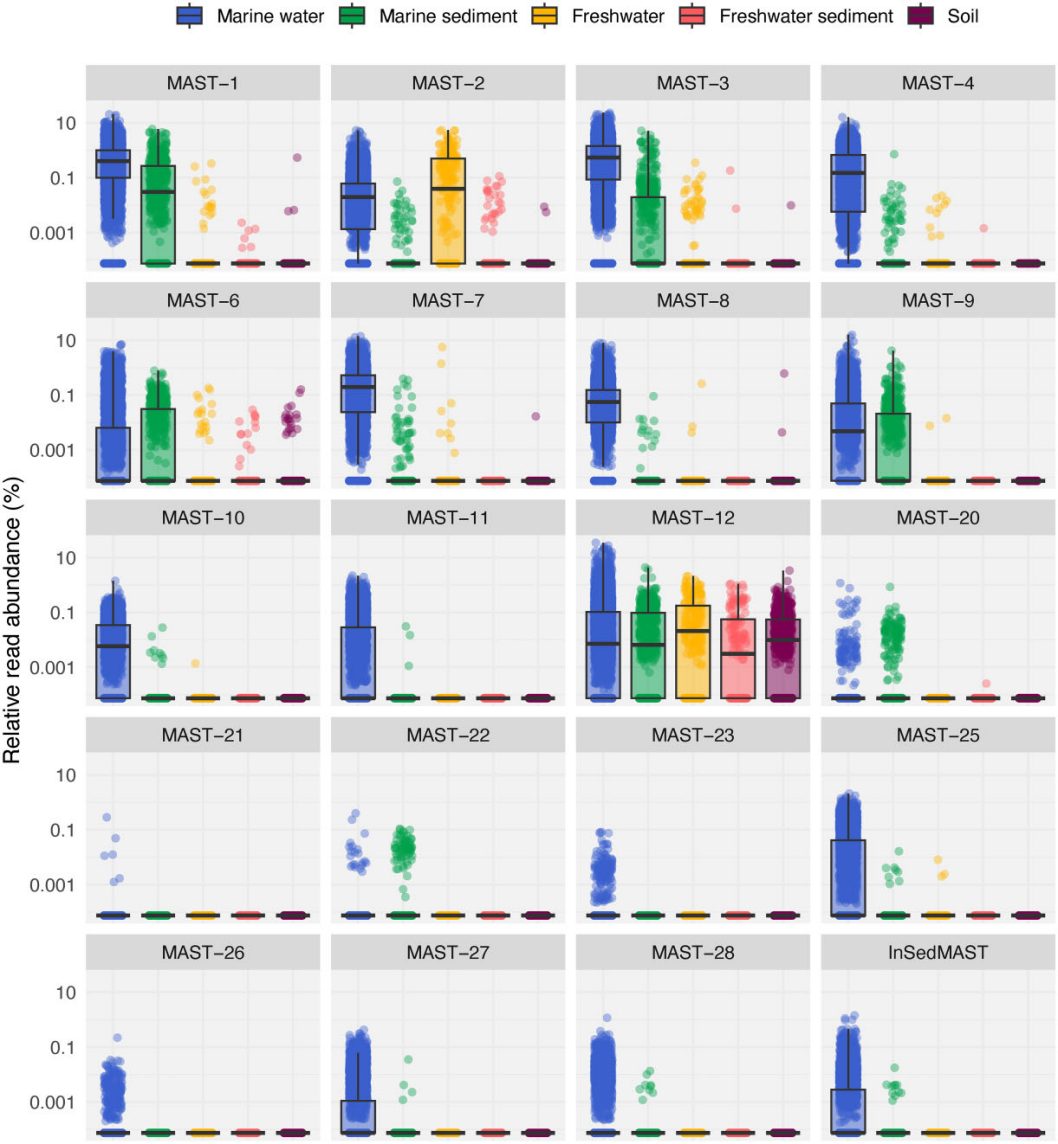
Distribution of MAST clades among different habitats. Relative read abundance of MAST clades in the five sampled habitats. Each dot represents the pooled relative read abundance of all ASVs belonging to a given clade in a sample. Values are displayed in logarithmic scale using the lowest positive value in the dataset as a pseudocount. Groups are arranged by name.

Most of the MAST clades were marine and, more specifically, pelagic (Fig. 3; Table 1). Some groups also displayed markedly high relative read abundances in marine sediments, namely MAST-1, -3, -6, and -9 (Fig. 3). Most of this signal derived from specific subclades: MAST-1C, MAST-3 J, MAST-6A, and MAST-9A (Figure S2). Only two clades, MAST-2 and -12, deviated from this clear marine preference and had a strong presence in terrestrial habitats (Fig. 3). For MAST-2, this was explained by subclade A, which was freshwater-specific, while the other subclades followed the most common marine distribution (Figure S2). In the case of MAST-12, this was the only clade showing a relatively high abundance in all habitats. This was again explained by the different preferences of its subclades (Fig. 3). Thus, while the majority of MAST-12 subclades displayed a clear marine pattern, MAST-12C and the newly described MAST-12H were terrestrial, and MAST-12C was the only subclade in the whole dataset with a strong signal in freshwater sediments and soils (Figure S2). The three newly described clades (MAST-26, -27, and -28) and the disparate collection of unclassified ASVs grouped under the InSedMAST category were primarily obtained from the marine water column (Fig. 3) and displayed a notable diversity in terms of the number of ASVs (Table 1). In our primary analysis of water column samples, we targeted protists smaller than 10 µm in size, but most of the samples belonged to the picoplanktonic fraction (0.2–3 µm) (Table S2). When considering picoplanktonic (4720 samples) and nanoplanktonic (≥3 µm; 1095 samples) fractions separately, the majority of MAST groups were also found in the nanoplanktonic fraction but generally at lower abundances (Figure S3). Only in a few cases, namely MAST-1, -2, and -12, the relative read abundances were comparable or even higher in the nanoplanktonic fraction (Figure S3).

**Figure 4.**
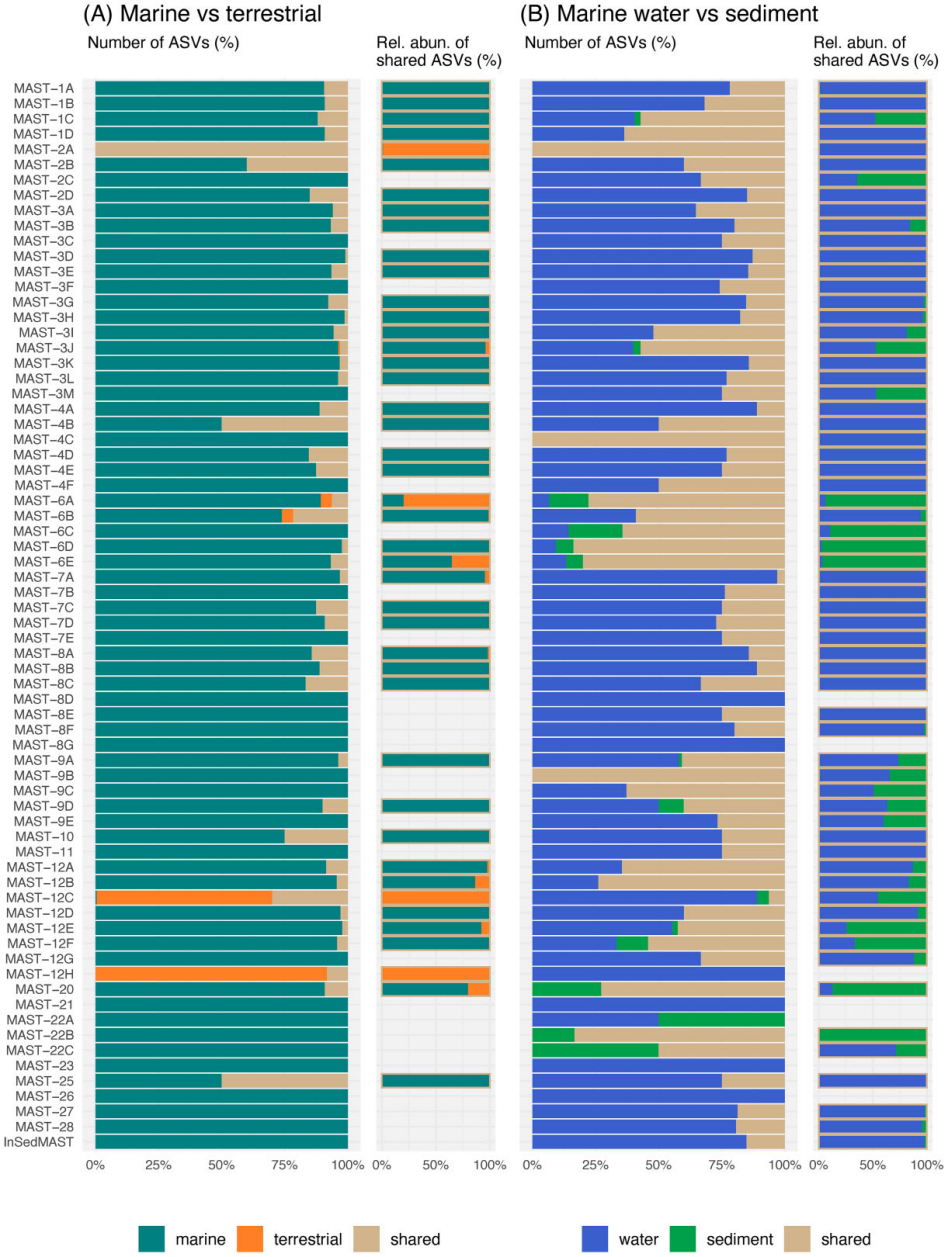
Habitat occupancy of MAST ASVs. (A) Comparison between marine (marine sediment + marine water) and terrestrial (freshwater + freshwater sediment + soil) habitats and (B) between marine sediments and marine water. For each comparison, the percentages of unique and shared ASVs between habitats are displayed, as well as the mean relative abundance of the shared ASVs in both habitats.

We then did a similar habitat analysis for all the individual MAST ASVs detected in the dataset. The vast majority of ASVs were unique to the marine environment and were not detected in terrestrial samples (Fig. 4A). The opposite situation, ASVs unique to terrestrial habitats, was rare and restricted to some MAST-6 and -12 subclades (Fig. 4A). Despite this marine dominance, many MAST subclades had a few ASVs present in both marine and terrestrial habitats. However, these ASVs tended to have a higher relative read abundance in marine habitats than in terrestrial ones (Fig. 4A) and were among the most abundant in marine datasets (Figure S4). Given that most ASVs were typically marine, we then analysed their presence in the plankton versus sediment. The majority of ASVs were either uniquely present in the water column or both in water and sediment (Fig. 4B). ASVs uniquely present in the sediment belonged mainly to MAST-6, -12, and -22 subclades. Generally, ASVs present in both the water column and sediments were more abundant in the former (Fig. 4B).

### The most abundant marine ASVs

**Figure 5.**
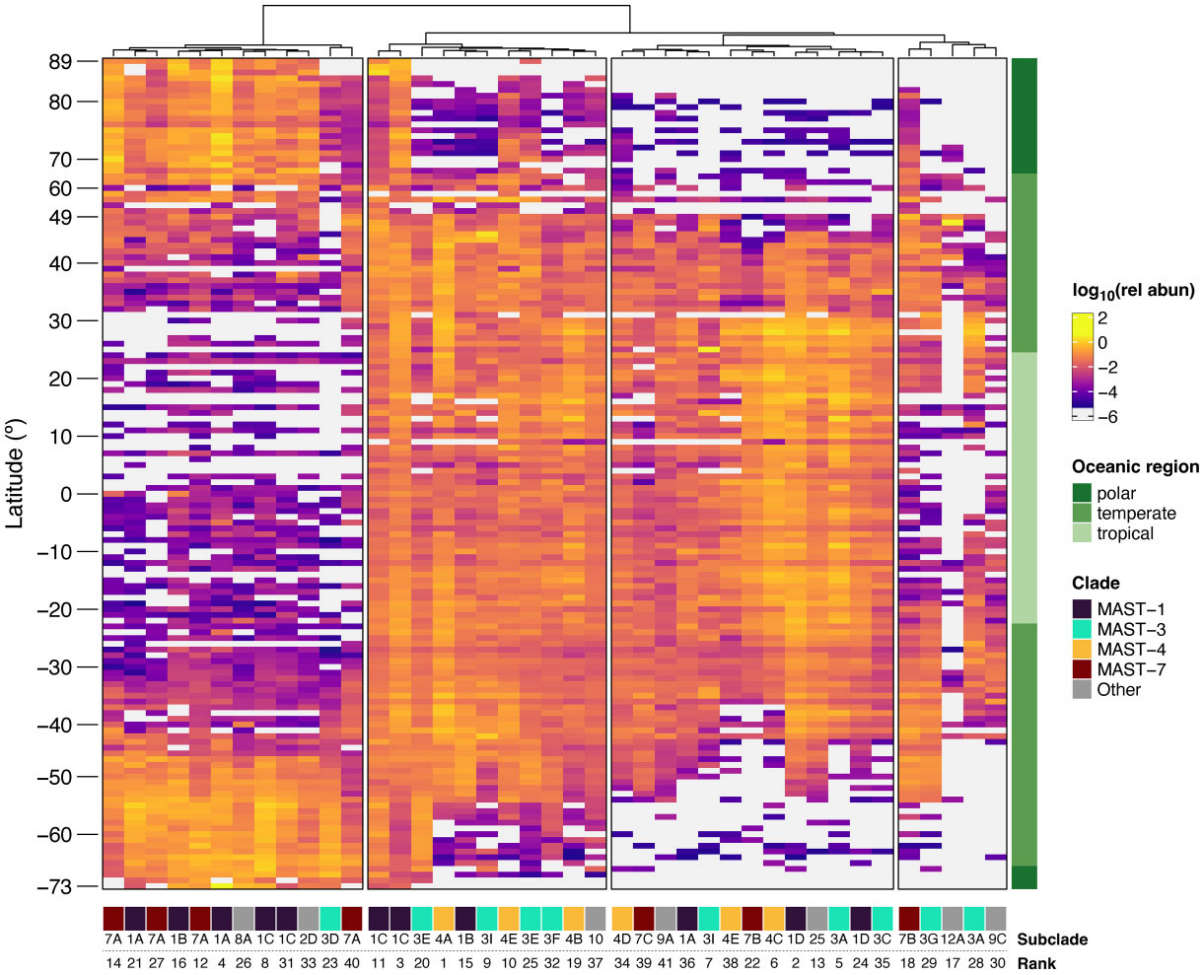
Latitudinal distribution of the 41 most abundant MAST in the ocean. Samples were pooled by latitude in intervals of one degree and the mean relative read abundance of each ASV within each interval was calculated. Values were then transformed to a logarithmic scale using the lowest positive value in the dataset as a pseudocount. Clustering of ASVs was performed with Euclidean distances using Ward D2 method. ASVs are grouped according to this clustering. Latitudinal ranges are coloured by oceanic region (polar, temperate, or tropical) and ASVs are coloured according to the MAST clade they belong to. The numbers displayed below each ASV represent their rank of abundance (i.e. number 1 is the most abundant ASV in the analysed dataset).

Finally, we evaluated the biogeographic distribution in marine planktonic samples of the most abundant ASVs, those that collectively accounted for 60% of the MAST signal (Table S5). This resulted in only 41 ASVs belonging to 10 different MAST clades, with predominant representation from MAST-1, -3, -4, and -7 (Fig. 5). These highly abundant ASVs displayed four different patterns according to their latitudinal distribution: (1) predominantly found in high-latitude regions, (2) widely distributed, (3) absent in high-latitude regions but present in temperate and tropical waters, and (4) primarily located in temperate waters (Fig. 5). Across most clades, more than one of these patterns could be observed (Fig. 5). High-latitude ASVs were primarily associated with MAST-1 and -7, while globally widespread ASVs belonged to MAST-1, -3, and -4, excluding -7. ASVs with widespread distribution but absent from high-latitude regions were assigned to all mentioned clades, whereas ASVs predominantly found in temperate waters were linked to MAST-3 and -7.

## Discussion

### An updated phylogenetic framework for MAST diversity

The first objective of our study was to construct an updated 18S rRNA gene phylogeny to hold possible new MAST clades within Stramenopiles. Despite working with a single gene, our Stramenopiles topology aligned with recent phylogenomic analyses. Specifically, Pseudofungi was the most closely related group to Ochrophyta, and Sagenista and Opalozoa formed separate groups (Azuma et al. 2022, Cho et al. 2023). Due to the prevailing lack of cultured species, from which reference genomes could be obtained (del Campo et al. 2023), recent advances in single-cell genomics (Labarre et al. 2021, Seeleuthner et al. 2018), metagenomics (Delmont et al. 2022), and metatranscriptomics (Obiol et al. 2023) have become crucial sources of new genomic data. Current phylogenomic trees have begun to incorporate some MAST species retrieved from single cell genomics, but they still lack the vast majority of MAST diversity. Therefore, new genomic data, both from cultured and uncultured species, is still needed to enhance our understanding of Stramenopiles evolution and deep tree topology.

In the last reevaluation of MAST diversity, hundreds of newly obtained long 18S rRNA gene sequences were used (Massana et al. 2014) thanks to the general use of clone libraries and Sanger sequencing. However, in the following years, the release of new long 18S rRNA gene sequences decreased drastically, mainly due to the adoption of high-throughput short-read sequencing. Fortunately, new sequencing platforms based on long reads are starting to be used in metabarcoding surveys (Jamy et al. 2020, 2022) and could reverse this trend. Even with the low amount of nearly complete sequences added to our reevaluation, we were able to describe three new MAST lineages and 11 new subclades in already established clades. Additionally, we found a signal in short read data of putative new clades lacking a long reference sequence, something already reported in previous works (Obiol et al. 2021). Putting all this new diversity in the context of the EukBank dataset, these three new clades represented less than 0.5% of the overall MAST signal. Thus, the current framework of 18 clades was maintained (Massana et al. 2014), with the addition of three minor clades.

### MAST global distribution across systems

The use of the EukBank dataset, with 7788 curated metabarcoding samples from different habitats, enabled us to obtain the most comprehensive report of MAST global distribution to date. Our results show that MAST are mostly inhabiting marine environments as there was not a single clade or even a subclade completely absent from those samples. Thus, the answer to “How marine are MAST?” is straightforward: MAST are largely marine, with a few remarkable exceptions. Specifically, only three subclades deviated from this prevalent marine trend, namely MAST-2A, MAST-12C, and the newly described MAST-12H. These displayed a preference for freshwater habitats. MAST-12C was also highly present in soils, being the only MAST clearly present in this habitat. This image is highly consistent with the results reported in the last MAST reevaluation (Massana et al. 2014) and subsequent studies including terrestrial samples (Metz et al. 2022a, Simon et al. 2015, Singer et al. 2021). Thus, our results did not align with our initial hypothesis, as the massive increase in sequencing data did not substantially alter the current view of MAST diversity and distribution.

Even though the majority of MAST groups were mainly present in marine samples, we detected several ASVs from strictly marine groups (i.e. all except MAST-2A, -12C, and -12H) also in terrestrial samples. However, these shared ASVs displayed low read abundances (Fig. 4) and occurrences (Figure S5) in terrestrial samples, with only 14 ASVs being present in at least five of them. Their presence in terrestrial habitats could be explained through dispersal from marine waters, as ASVs were generally detected in sites near the ocean (Figure S6). In addition, these ASVs were generally very abundant in marine waters (Figure S4), therefore being the most susceptible to disperse to terrestrial habitats. Whether these ASVs represent species actively growing in freshwater or soil environments remains a question. A plausible explanation could be that these marine ASVs represent rare taxa in terrestrial habitats, the result of random dispersion, and incapable of thriving under these specific environmental conditions (Pedrós-Alió 2006). Overall, this minimal number of ASVs detected in both marine and terrestrial samples agrees with the fact that transition rates in non-Ochrophyta Stramenopiles are generally low (Jamy et al. 2022). The cases of MAST-2A, -12C, and -12H are significant exceptions to this rule. Interestingly, the timing of these transitions seems to vary among clades, as MAST-2A was represented by a single widespread ASV, while MAST-12C and -12H were formed by 210 and 24 ASVs, respectively. This discrepancy suggests that MAST-12 subclades may have transitioned earlier, leading to extensive diversification and further habitat colonization (in subclade C), while the transition of MAST-2A appears to be more recent. In marine habitats, most MAST ASVs were either exclusive to the water column or present in both the water column and the sediment. Similar to the dispersion from marine to terrestrial habitats, this could be explained by the sinking of planktonic species to the benthos and may represent taxa not able to thrive in sediments. However, there are also a few examples of MAST taxa exclusively found in sediments, mainly within MAST-6, -12, -20, and -22 (Lin et al. 2022, Piwosz and Pernthaler 2010). So, while the majority of MAST species seem to be planktonic, there are a few ones within particular phylogenetic clades that seem better adapted to a benthic existence.

In the analysed EukBank dataset the marine environment was overrepresented as compared to the rest of habitats. Moreover, the bulk of terrestrial samples were collected in the northern hemisphere (mainly Europe and North America), being Africa the continent with fewer (and nearly absent) samplings. ASV accumulation curves based on the number of samples indicate a clear saturation for marine water samples, which reached 80% of ASVs with just 631 samples (Figure S7). In contrast, the accumulation curves for the other habitats did not reach saturation, although their slopes began to decrease (Figure S7). Thus, while the sampling coverage of the ocean can be considered global and rather complete, the analysis of terrestrial habitats is largely partial. New insights could be gained with an increased sampling effort in soils, which exhibit higher patchiness (Geisen et al. 2018), as well as in freshwater systems from the southern hemisphere (Metz et al. 2022b) and high-mountain lakes (Boenigk et al. 2018). Overall, there is the possibility that extending the sampling coverage could reveal additional terrestrial MASTs. Marine anoxic environments were also absent in the analysed EukBank dataset. Notably, here we did not detect any sequences related to MAST-16 and -24, which remain exclusive to anoxic habitats (Massana et al. 2014). Other previously thought anoxic clades, namely MAST-20, -21, -22, and -23, were found here in nonanoxic samples, but the lack of anoxic samples in the EukBank dataset did not allow to establish their oxic–anoxic preference. Since the last MAST reevaluation, only a few metabarcoding studies have been conducted in anoxic habitats, primarily in oxygen minimum zones of the Pacific Ocean (Duret et al. 2015, Fuchsman et al. 2022, Parris et al. 2014) and in sulfidic waters of the Black Sea (Wylezich et al. 2018). While some of these studies detected a minor presence of previously described MAST groups, the sequencing efforts were generally insufficient to draw clear conclusions. Therefore, more extensive datasets are still required to reveal solid patterns of the distribution of MAST groups in marine anoxic habitats.

### MAST global distribution in the marine water column

Our findings underscore the significance of MAST in the marine water column. We detected them in 99% of the samples from this habitat, and represented around 3% of the whole protist signal on average (Table S3; Figure S8). The majority of these clades appeared to contain pico-sized (0.2–3 µm) species, as their abundances were in general higher in this size fraction than in the nanoplanktonic fraction. This aligns with previous microscopy studies that have documented similar cell pico-sizes (Mangot et al. 2018, Piwosz et al. 2021). To date, there is no evidence of photosynthetic species within MAST groups (Labarre et al. 2021) and some studies have reported phagotrophic activity in certain clades, with also a case of a parasitic member (Gómez et al. 2011). Besides, bacterial grazing in the ocean is primarily mediated by heterotrophic flagellates within the 2–5 µm size range (Jürgens and Massana 2008). Thus, MAST species exhibiting a globally widespread distribution and relatively high abundances are clearly main players in marine food web dynamics and are expected to play a crucial role as pico-sized bacterial grazers. In terms of diversity, the number of ASVs greatly varied among clades. For instance, having similar overall abundances, MAST-3 represented 36% of all MAST ASVs, while MAST-4 only accounted for just 1.7%. Thus, albeit assumed to be mostly picoplanktonic heterotrophic protists, each MAST clade has a completely distinct evolutionary story (Latorre et al. 2021) that awaits to be unravelled.

At the level of specific sequences, very few ASVs represented the majority of the signal in the marine water column. A similar trend was reported in a previous study targeting marine heterotrophic flagellates in the tropical and subtropical ocean (Obiol et al. 2021). Here, the inclusion of polar and temperate ocean zones allowed us obtaining global patterns of the most abundant MAST species. The observed latitudinal patterns seemed to be clearly governed by temperature, which is the main driving factor in microbial communities (Ibarbalz et al. 2019). Nevertheless, we detected some ASVs, especially two MAST-1C sequences (Fig. 5), that did not seem to be limited by temperature, as they were present in nearly all analysed samples. These could either represent single species adapted to thrive on any latitude or different ecological species that are hidden within the same ASV. Additionally, we also detected three MAST-4 ASVs present in polar regions, which were previously thought to be absent from these waters due to temperature barriers (Rodríguez-Martínez et al. 2013). These may become sentinels of climate change, as they could inform of new inputs of temperate waters or temperature shifts in polar sites. As expected, none of the most abundant 41 ASVs represent cultured species, highlighting the lack of model species in this assemblage.

## Conclusion

In the present study, we perform a comprehensive reevaluation of MAST diversity and distribution at a global scale and across different habitats. Our updated MAST phylogeny confirms the existing framework while introducing three new MAST clades and 11 new subclades, enriching our understanding of their evolutionary relationships. Contrary to our initial hypothesis, the substantial increase in sequencing data, both in terms of sequencing depth and sampled habitats, did not fundamentally alter the current understanding of MAST ecology. MAST are primarily marine, with only a few clear cases of marine–freshwater transitions and diversifications. They are mostly planktonic and belong to the pico-sized fraction. Our findings underscore the relevance of MAST in the ocean, suggesting their pivotal role in bacterial grazing dynamics. In addition, they highlight the need for further exploration of these uncultured lineages, particularly through culturing efforts and single-cell genomic studies, to gain a deeper understanding of these significant components of marine ecosystems.

## Supplementary Material

fiae130_Supplemental_Files

## Data Availability

The EukBank dataset is available in Zenodo with DOI 10.5281/zenodo.7804945. All code used for data processing and analyses is available in GitHub (https://github.com/aleixop/mast_eukbank).
