# Supplementary material for: How marine are Marine Stramenopiles (MAST)? A cross-system evaluation
Source: FEMS Microbiol Ecol. 2024 Oct 7;100(11):fiae130. doi: 10.1093/femsec/fiae130 (PMC11523054; doi:10.1093/femsec/fiae130)
Supplement: fiae130_Supplemental_Files [file fiae130_supplemental_files.zip › figureS4.pdf]

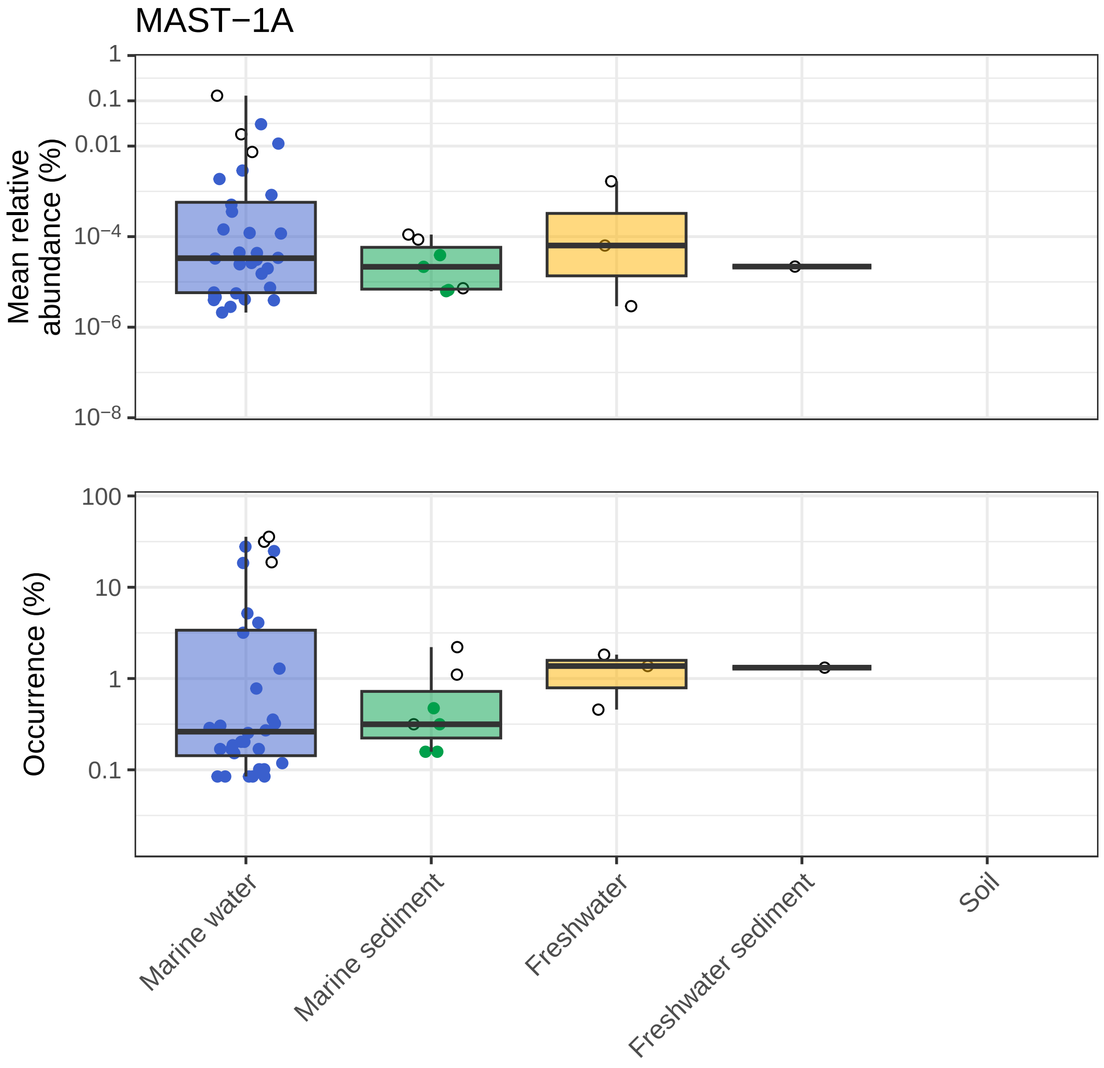

MAST−1A
1
0.1
0.01
10^{-4}
10^{-6}
10^{-8}
Mean relative abundance (%)
100
10
1
0.1
Occurrence (%)
Marine water
Marine sediment
Freshwater
Freshwater sediment
Soil

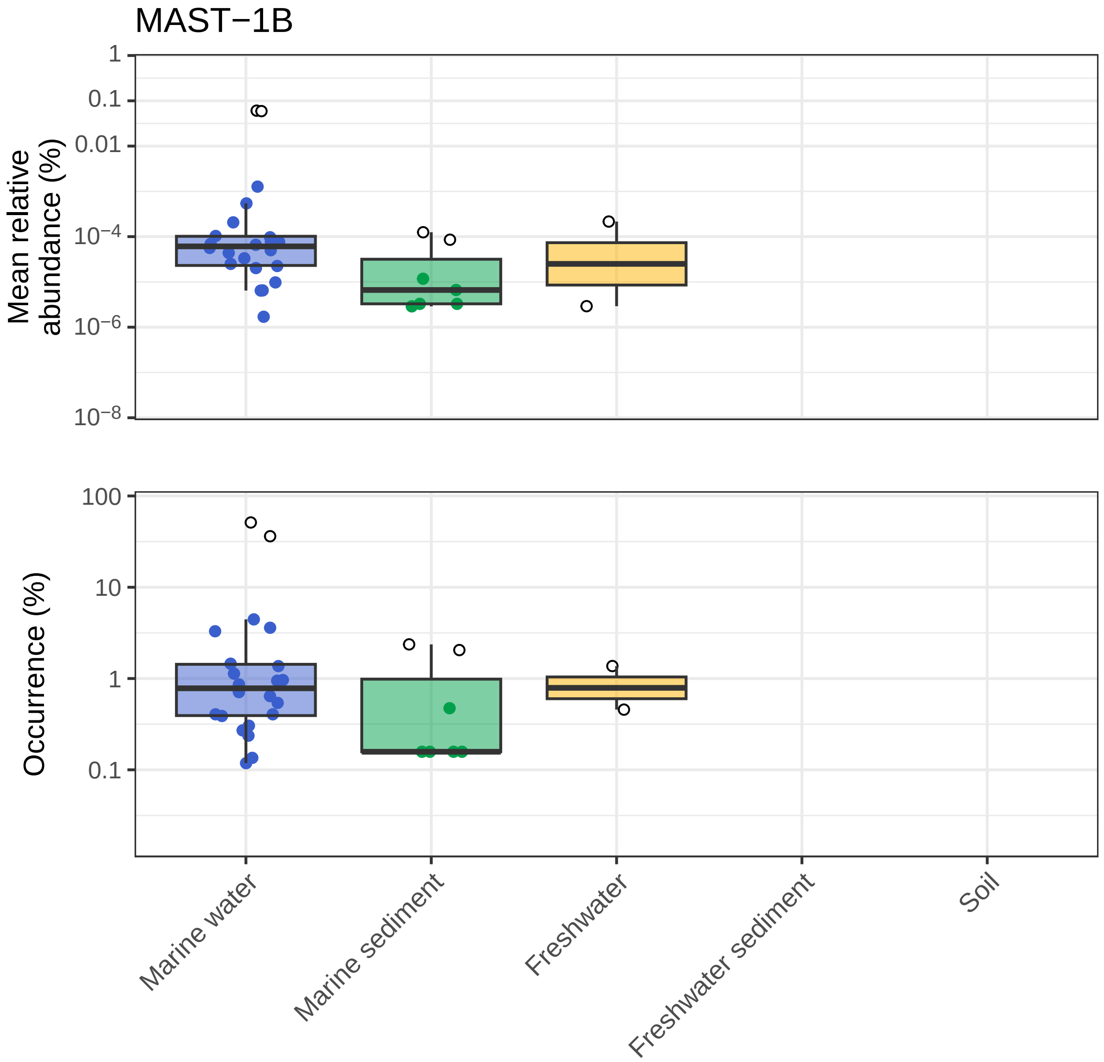

MAST−1B
Mean relative abundance (%)
1
0.1
0.01
10−4
10−6
10−8
Occurrence (%)
100
10
1
0.1
Marine water
Marine sediment
Freshwater
Freshwater sediment
Soil

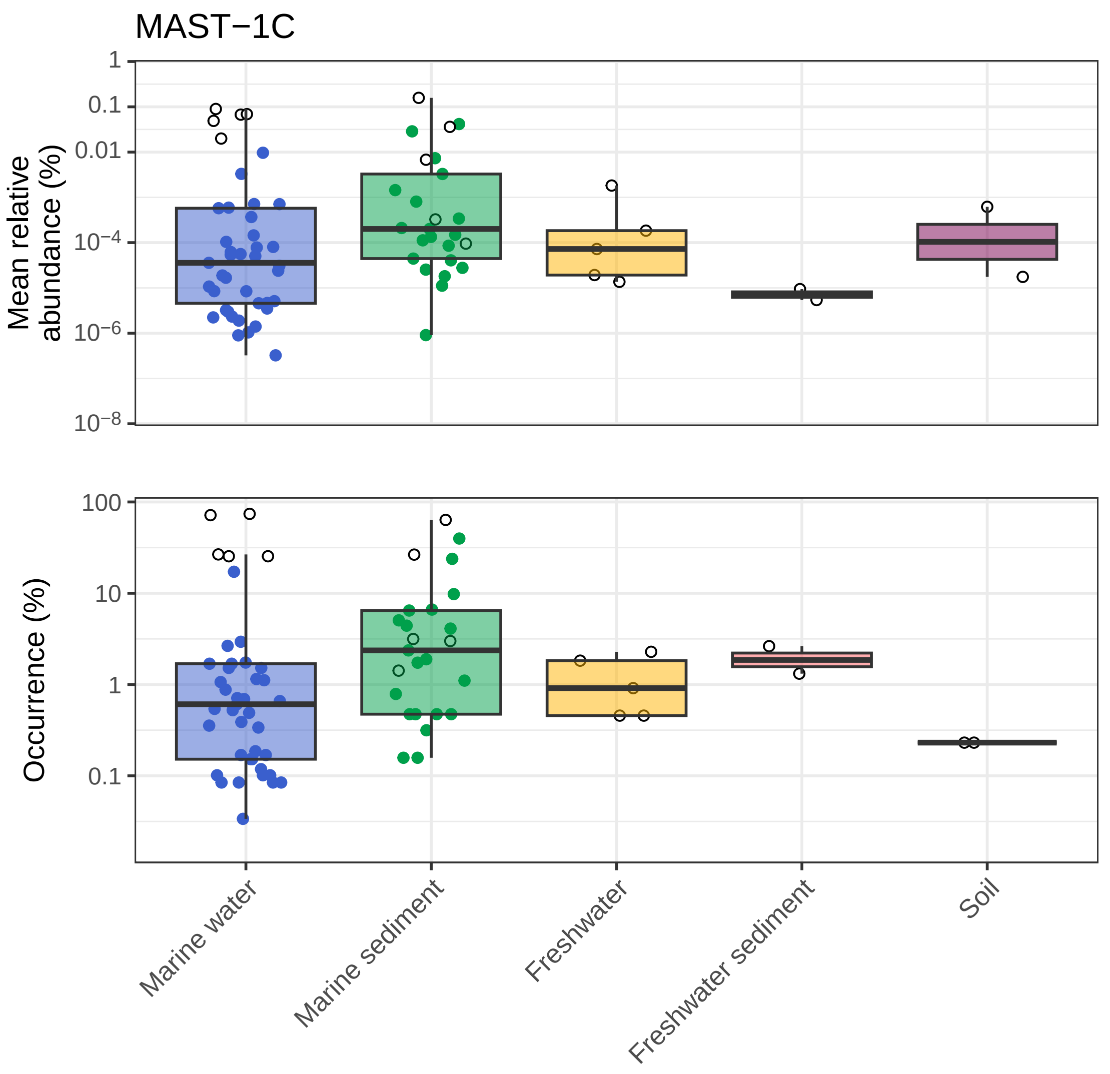

MAST−1C
Mean relative abundance (%)
1
0.1
0.01
10^{-4}
10^{-6}
10^{-8}
Occurrence (%)
100
10
1
0.1
Marine water
Marine sediment
Freshwater
Freshwater sediment
Soil

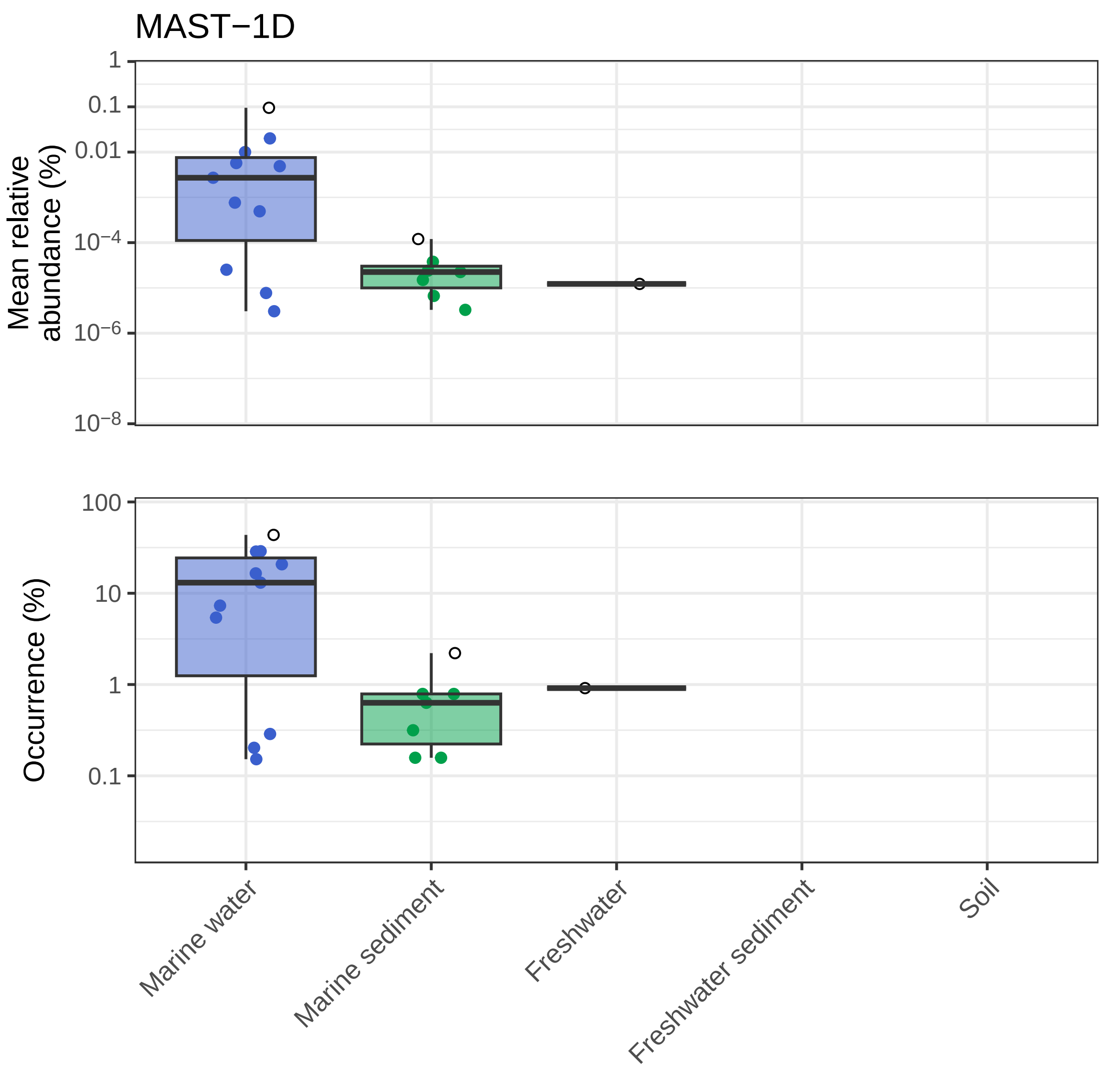
MAST−1D
Mean relative abundance (%)
1
0.1
0.01
10−4
10−6
10−8
Occurrence (%)
100
10
1
0.1
Marine water
Marine sediment
Freshwater
Freshwater sediment
Soil

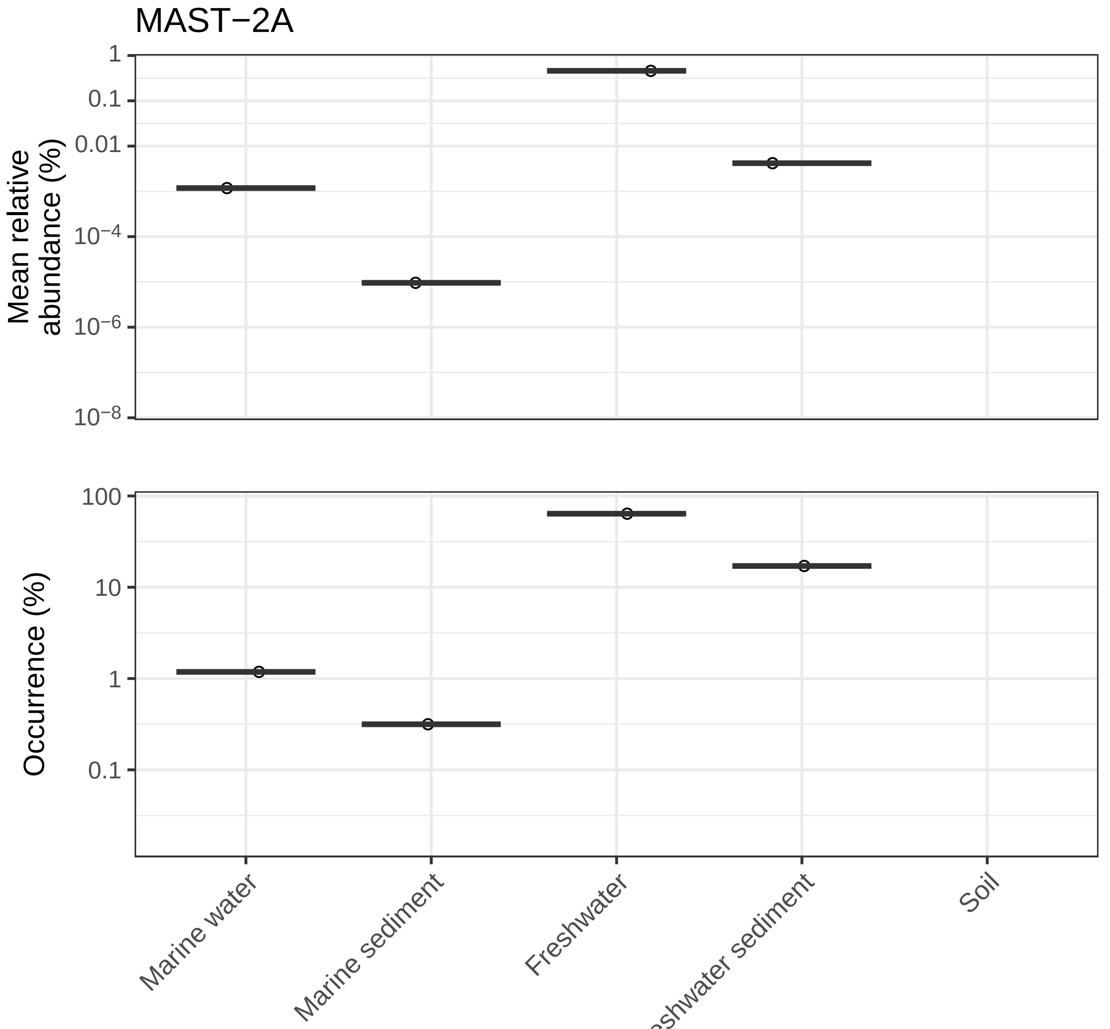

MAST−2A
Mean relative abundance (%)
1
0.1
0.01
10^{-4}
10^{-6}
10^{-8}
Occurrence (%)
100
10
1
0.1
Marine water
Marine sediment
Freshwater
Freshwater sediment
Soil

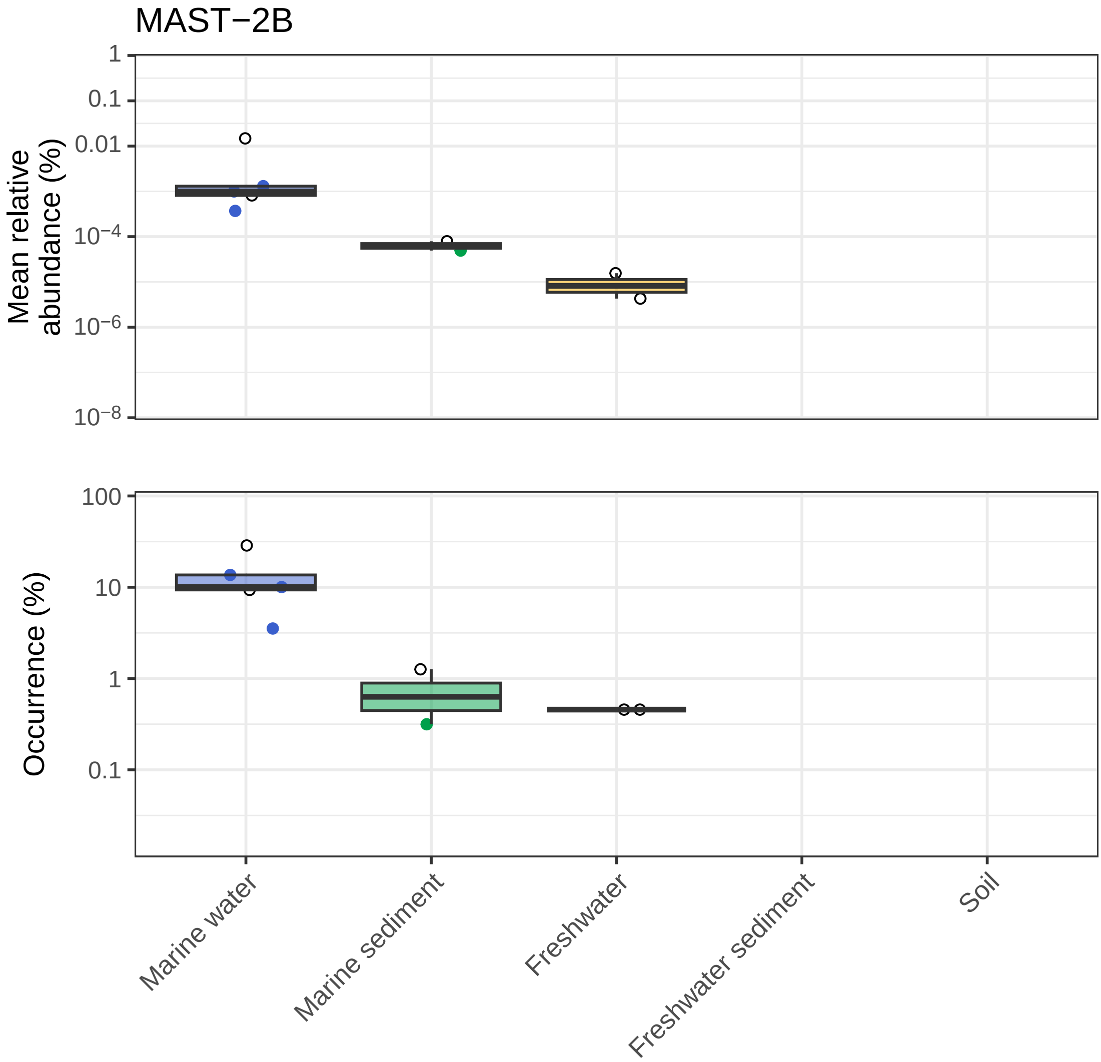

MAST−2B
Mean relative abundance (%)
1
0.1
0.01
10^{-4}
10^{-6}
10^{-8}
Occurrence (%)
100
10
1
0.1
Marine water
Marine sediment
Freshwater
Freshwater sediment
Soil

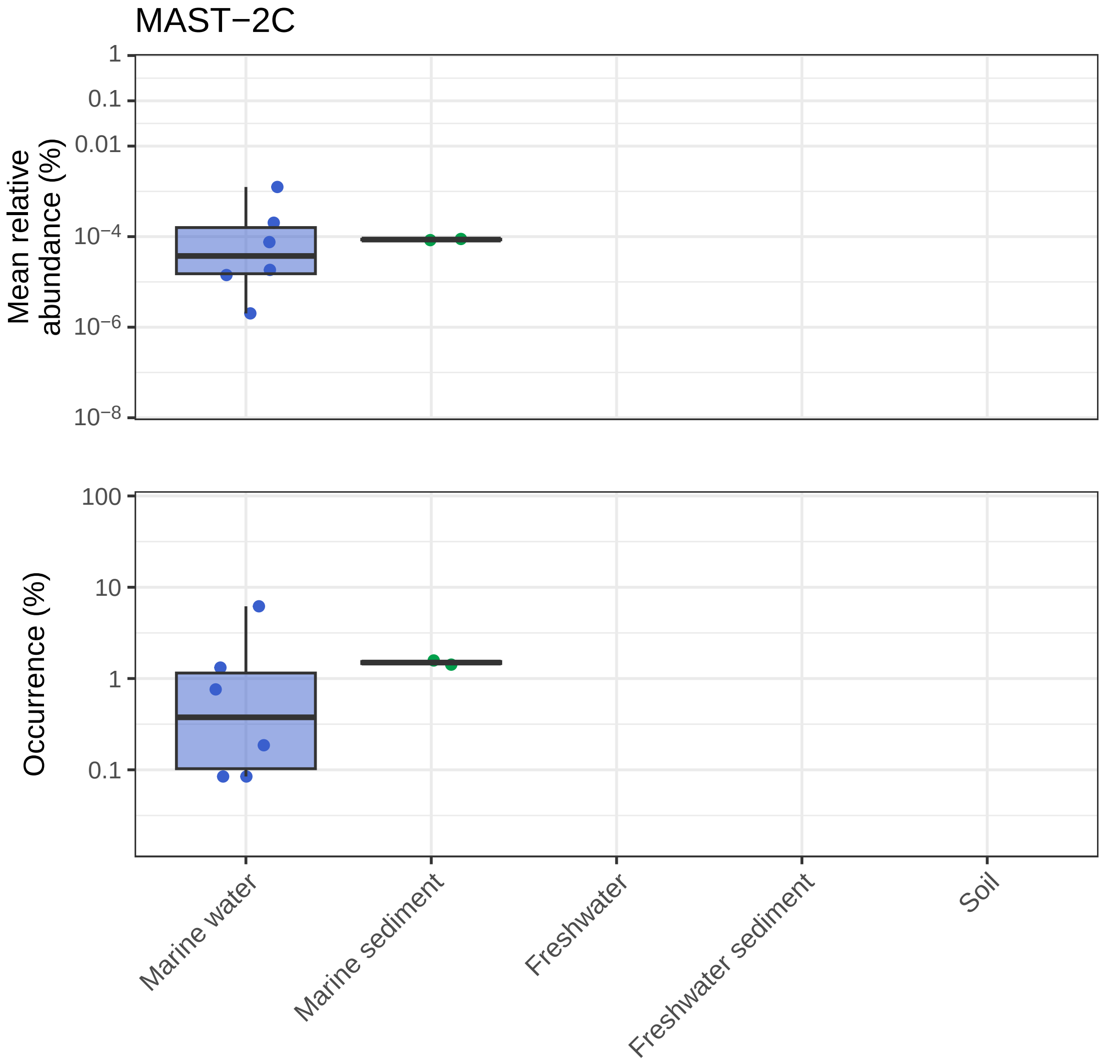
MAST−2C
Mean relative abundance (%)
1
0.1
0.01
10^{-4}
10^{-6}
10^{-8}
Occurrence (%)
100
10
1
0.1
Marine water
Marine sediment
Freshwater
Freshwater sediment
Soil

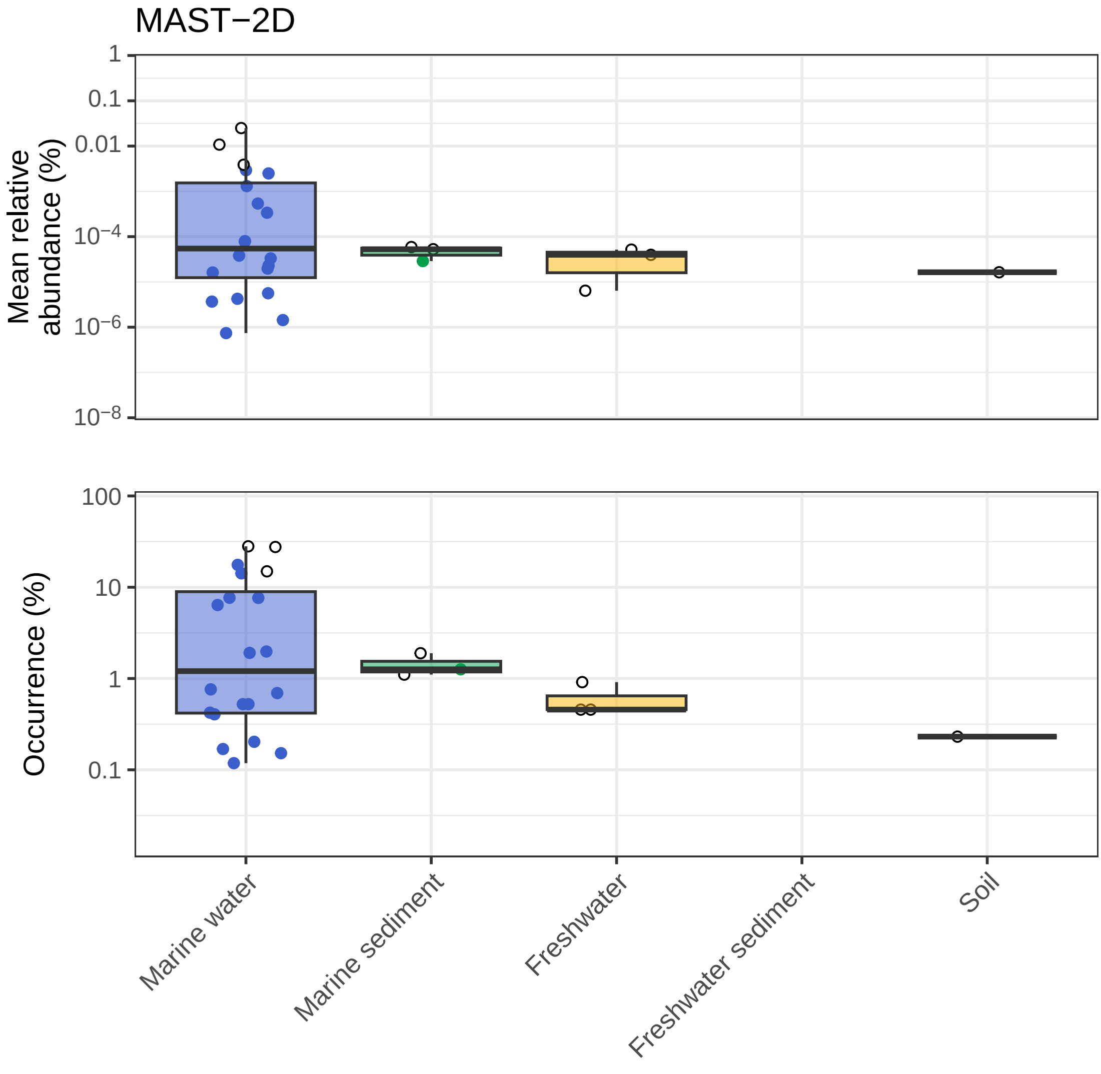

MAST−2D
Mean relative abundance (%)
1
0.1
0.01
10^{-4}
10^{-6}
10^{-8}
Occurrence (%)
100
10
1
0.1
Marine water
Marine sediment
Freshwater
Freshwater sediment
Soil

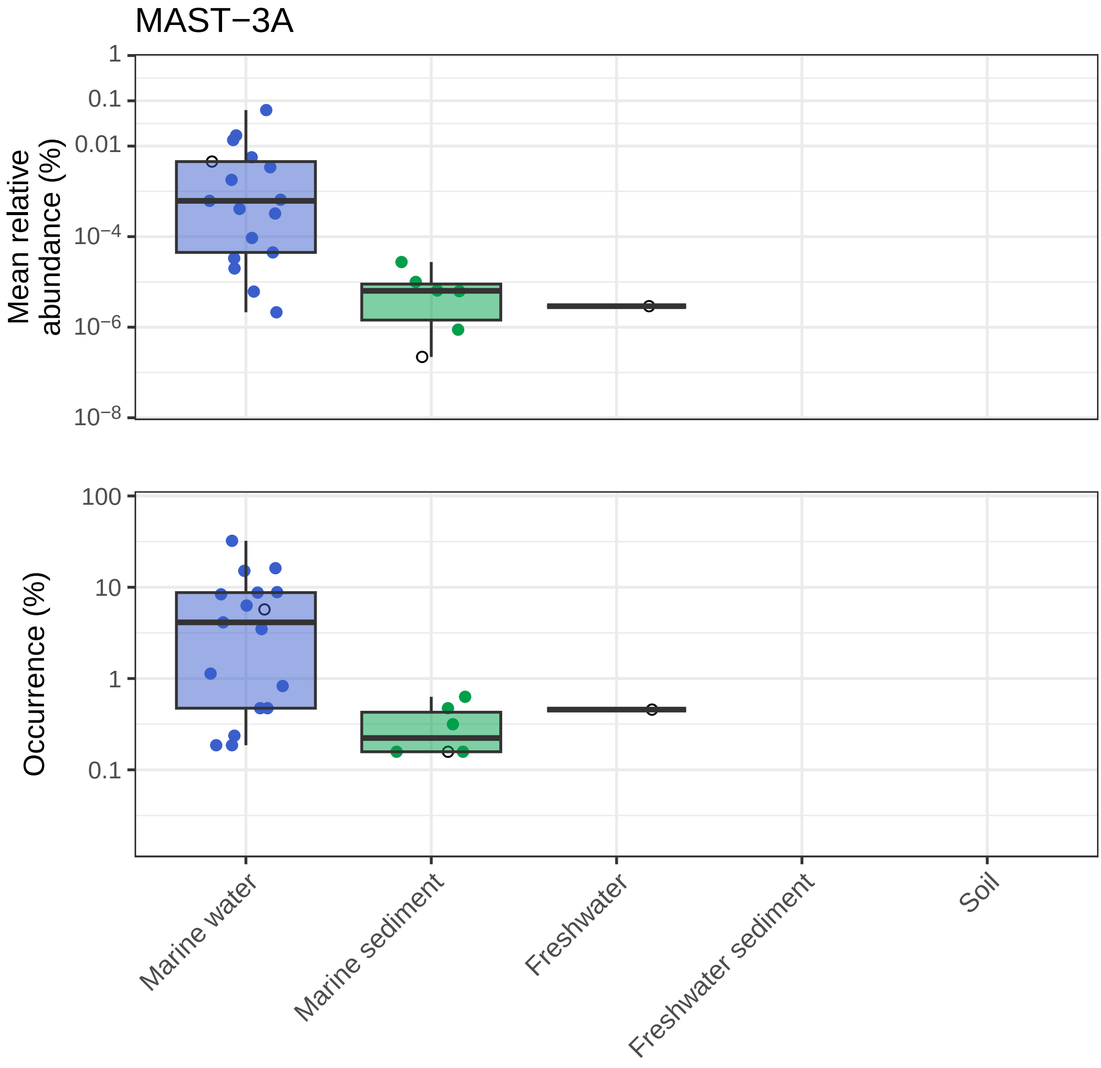

MAST−3A
Mean relative abundance (%)
1
0.1
0.01
10^{-4}
10^{-6}
10^{-8}
Occurrence (%)
100
10
1
0.1
Marine water
Marine sediment
Freshwater
Freshwater sediment
Soil

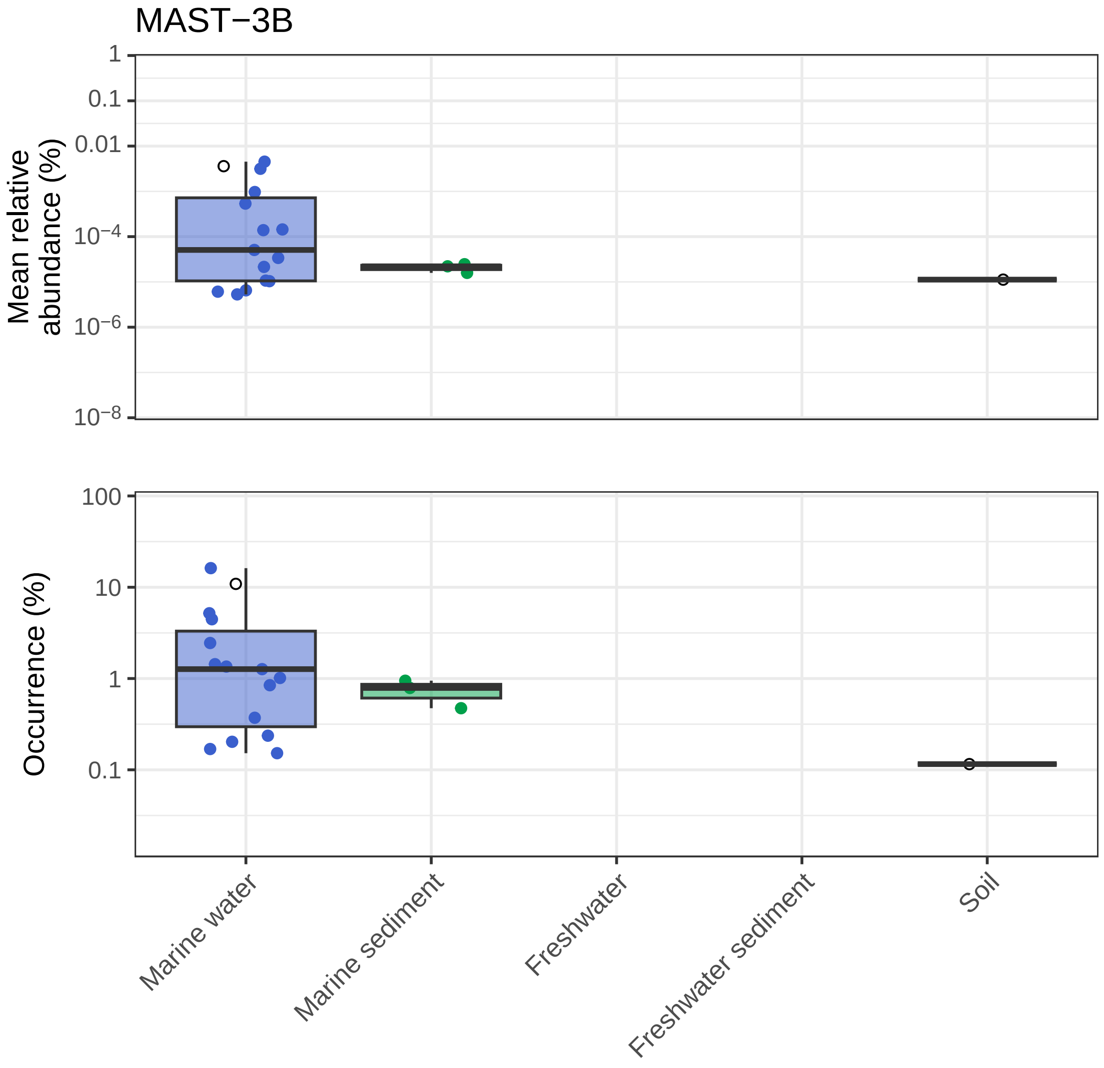

MAST−3B
Mean relative abundance (%)
1
0.1
0.01
10^{-4}
10^{-6}
10^{-8}
Occurrence (%)
100
10
1
0.1
Marine water
Marine sediment
Freshwater
Freshwater sediment
Soil

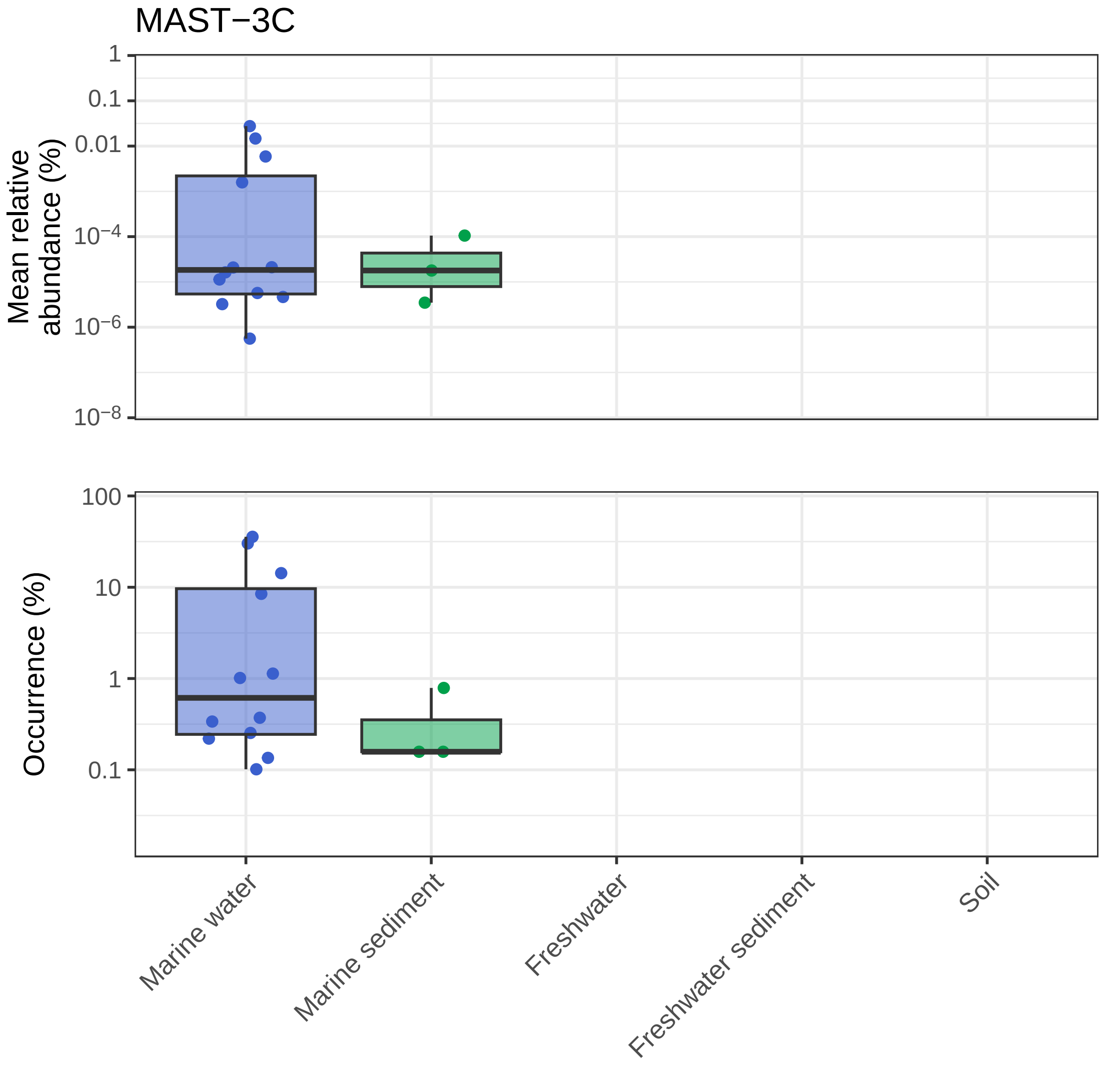

MAST−3C
Mean relative abundance (%)
1
0.1
0.01
10^{-4}
10^{-6}
10^{-8}
Occurrence (%)
100
10
1
0.1
Marine water
Marine sediment
Freshwater
Freshwater sediment
Soil

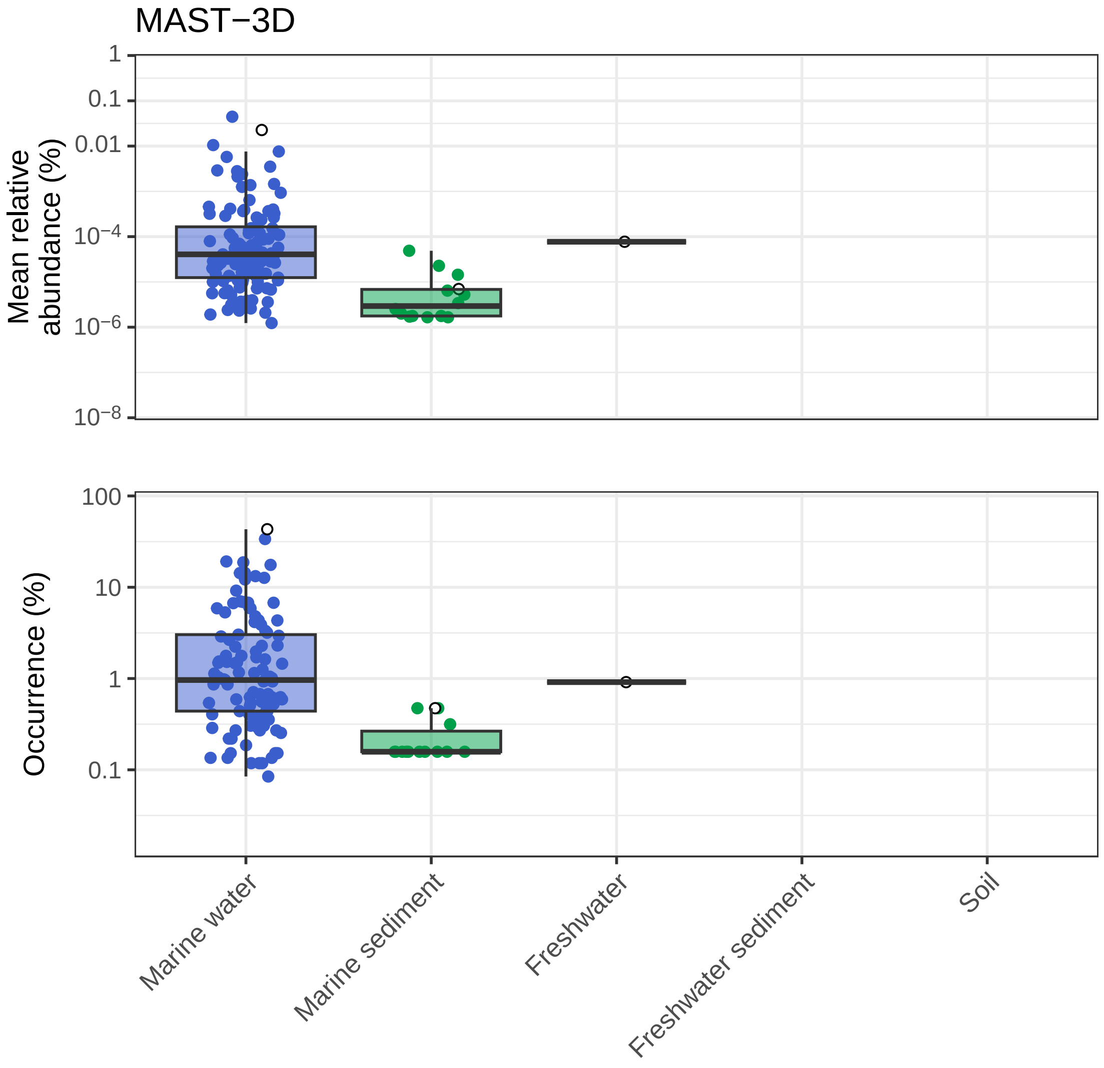

MAST−3D
Mean relative abundance (%)
1
0.1
0.01
10^{-4}
10^{-6}
10^{-8}
Occurrence (%)
100
10
1
0.1
Marine water
Marine sediment
Freshwater
Freshwater sediment
Soil

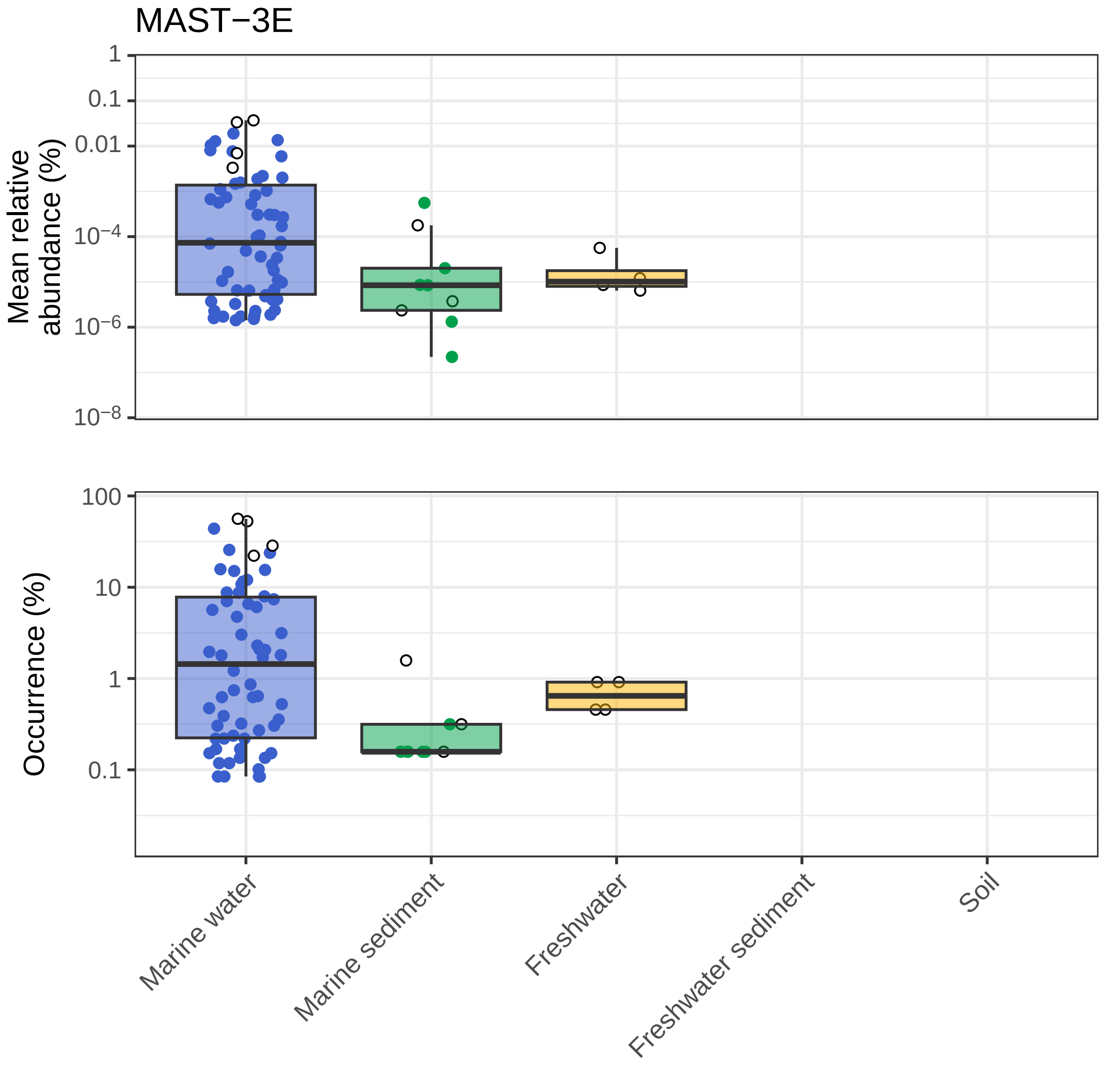

MAST−3E
Mean relative abundance (%)
1
0.1
0.01
10^{-4}
10^{-6}
10^{-8}
Occurrence (%)
100
10
1
0.1
Marine water
Marine sediment
Freshwater
Freshwater sediment
Soil

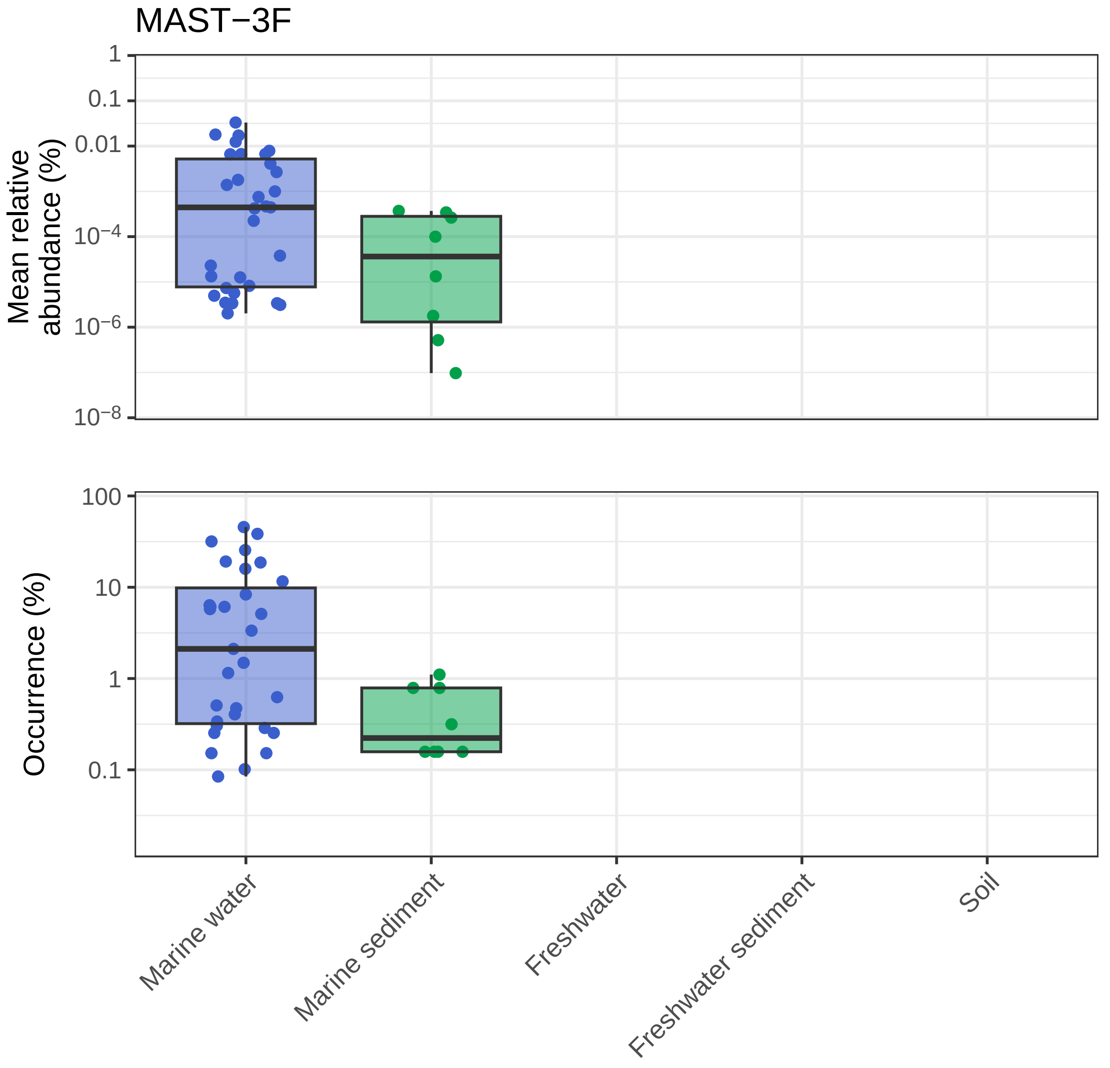
MAST−3F
Mean relative abundance (%)
1
0.1
0.01
10^{-4}
10^{-6}
10^{-8}
Occurrence (%)
100
10
1
0.1
Marine water
Marine sediment
Freshwater
Freshwater sediment
Soil

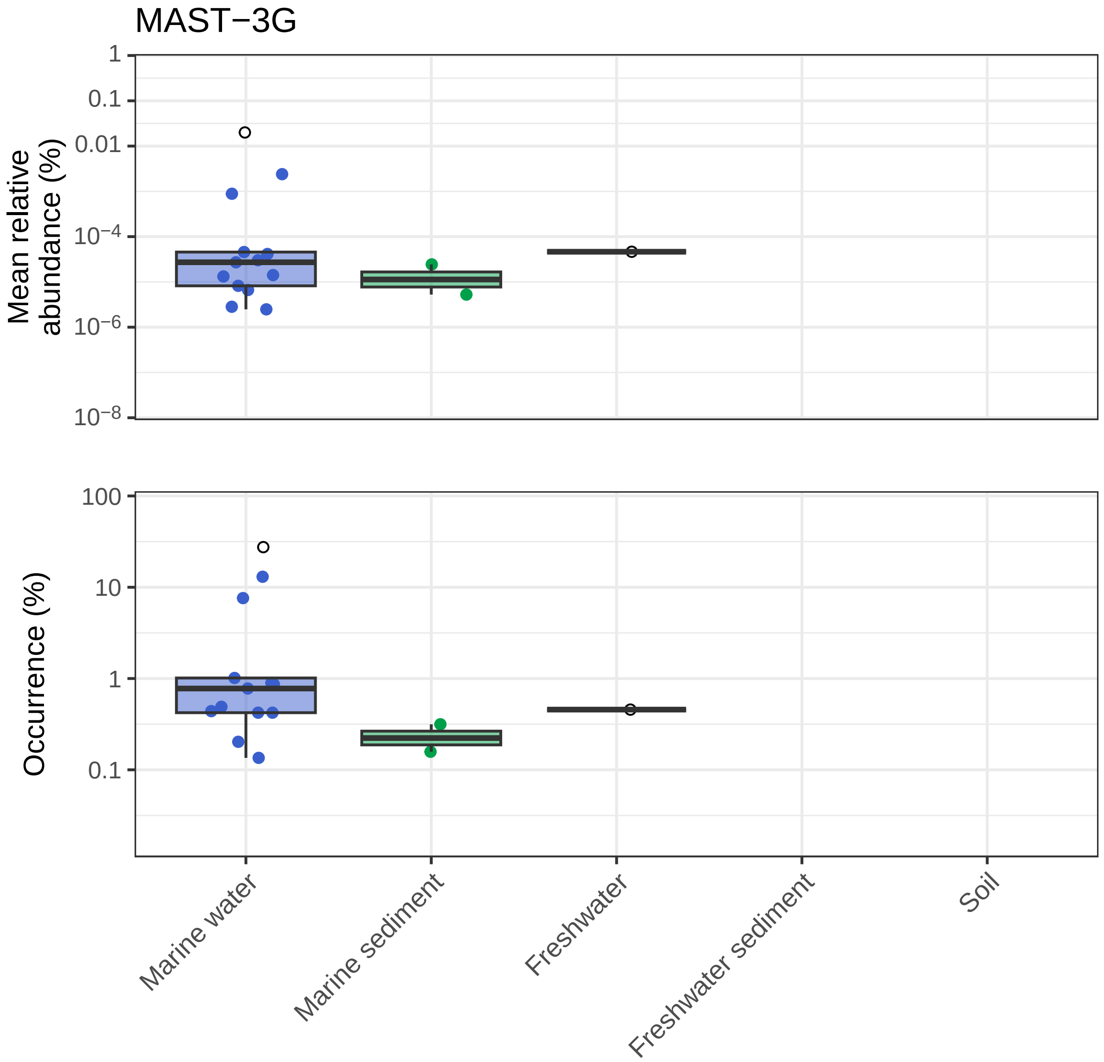
MAST−3G
Mean relative abundance (%)
1
0.1
0.01
10−4
10−6
10−8
Occurrence (%)
100
10
1
0.1
Marine water
Marine sediment
Freshwater
Freshwater sediment
Soil

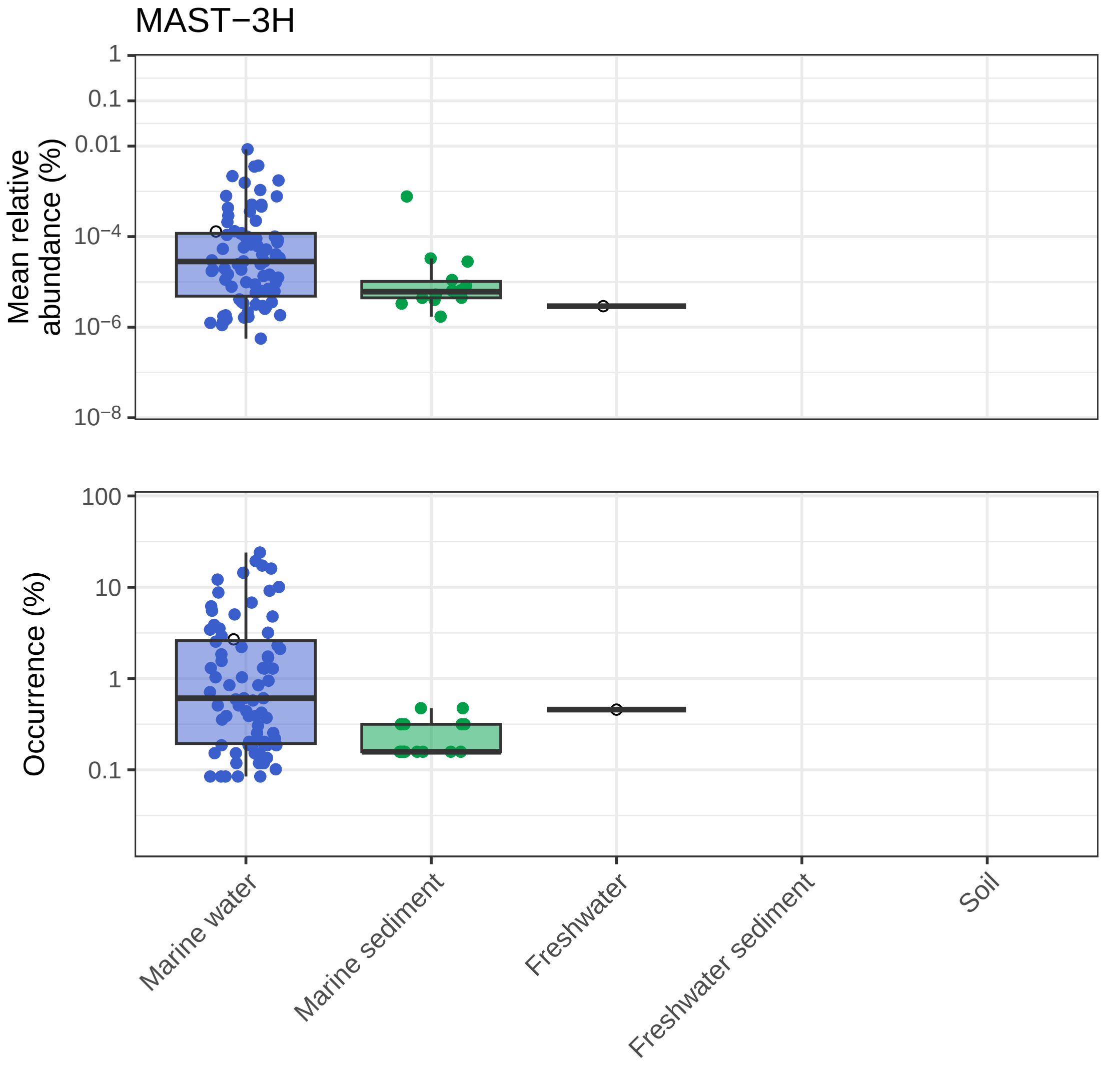

MAST−3H
Mean relative abundance (%)
1
0.1
0.01
10^{-4}
10^{-6}
10^{-8}
Occurrence (%)
100
10
1
0.1
Marine water
Marine sediment
Freshwater
Freshwater sediment
Soil

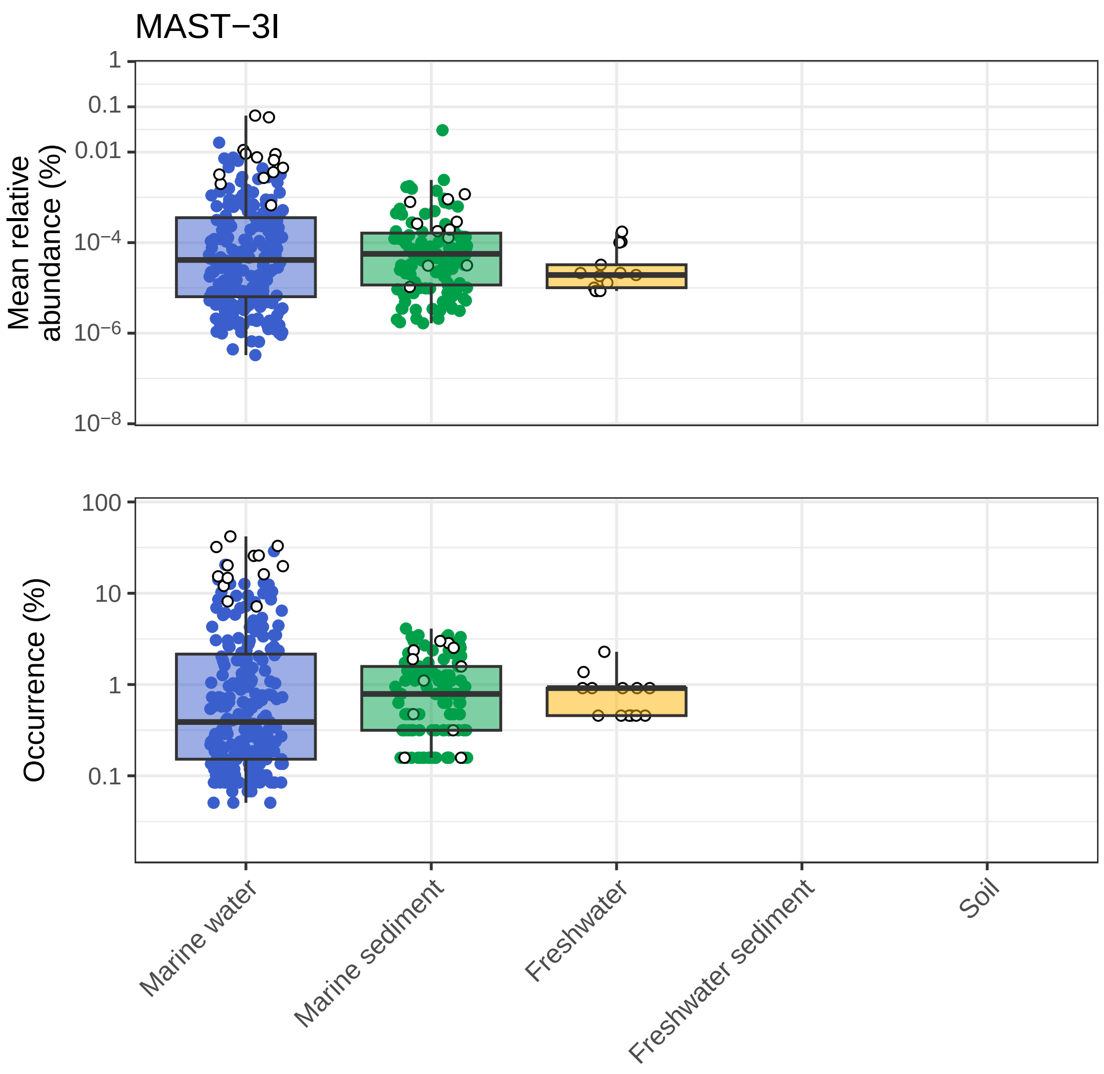

MAST−3I
Mean relative abundance (%)
1
0.1
0.01
10^{-4}
10^{-6}
10^{-8}
Occurrence (%)
100
10
1
0.1
Marine water
Marine sediment
Freshwater
Freshwater sediment
Soil

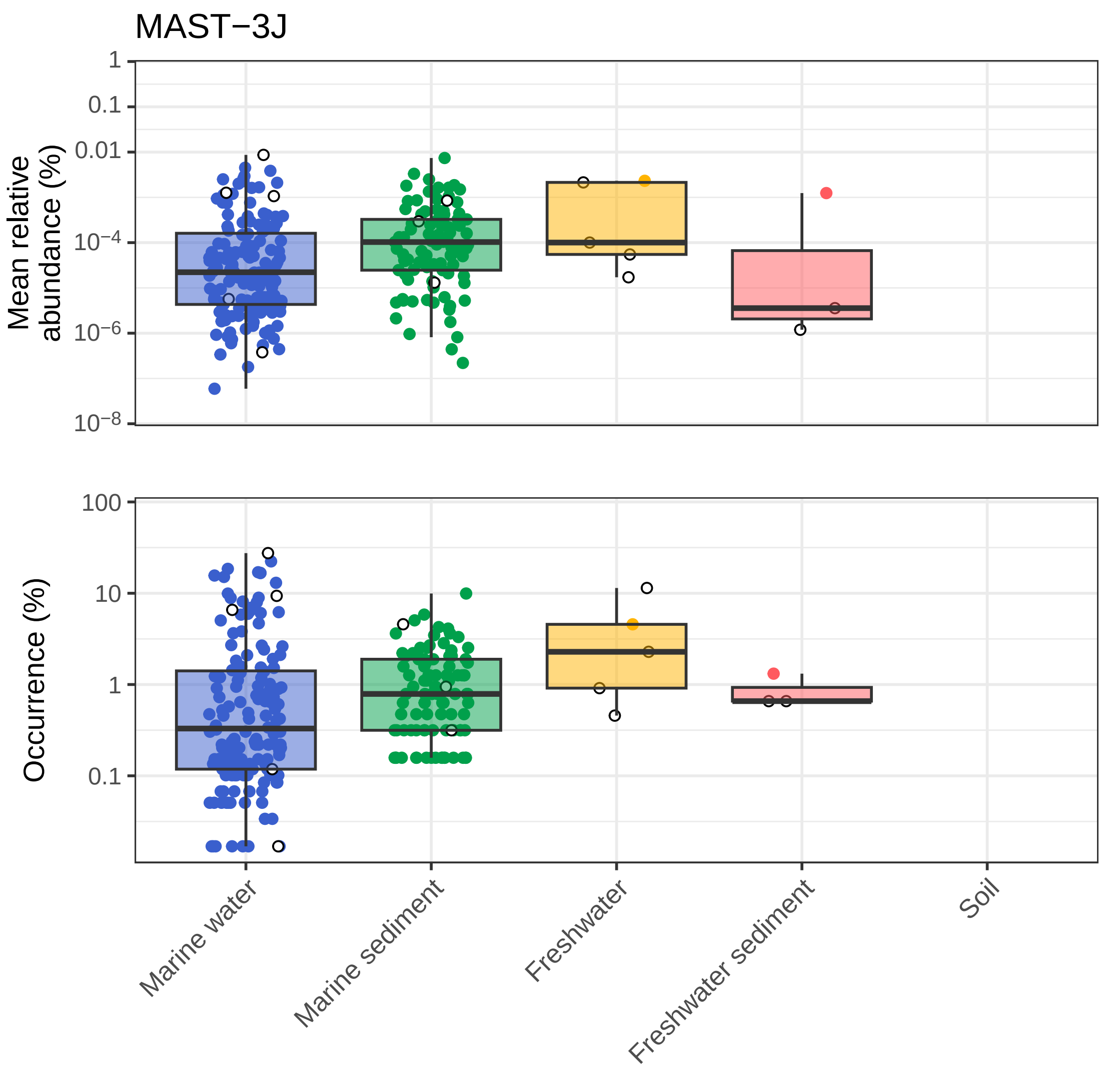

MAST−3J
Mean relative abundance (%)
1
0.1
0.01
10^{-4}
10^{-6}
10^{-8}
Occurrence (%)
100
10
1
0.1
Marine water
Marine sediment
Freshwater
Freshwater sediment
Soil

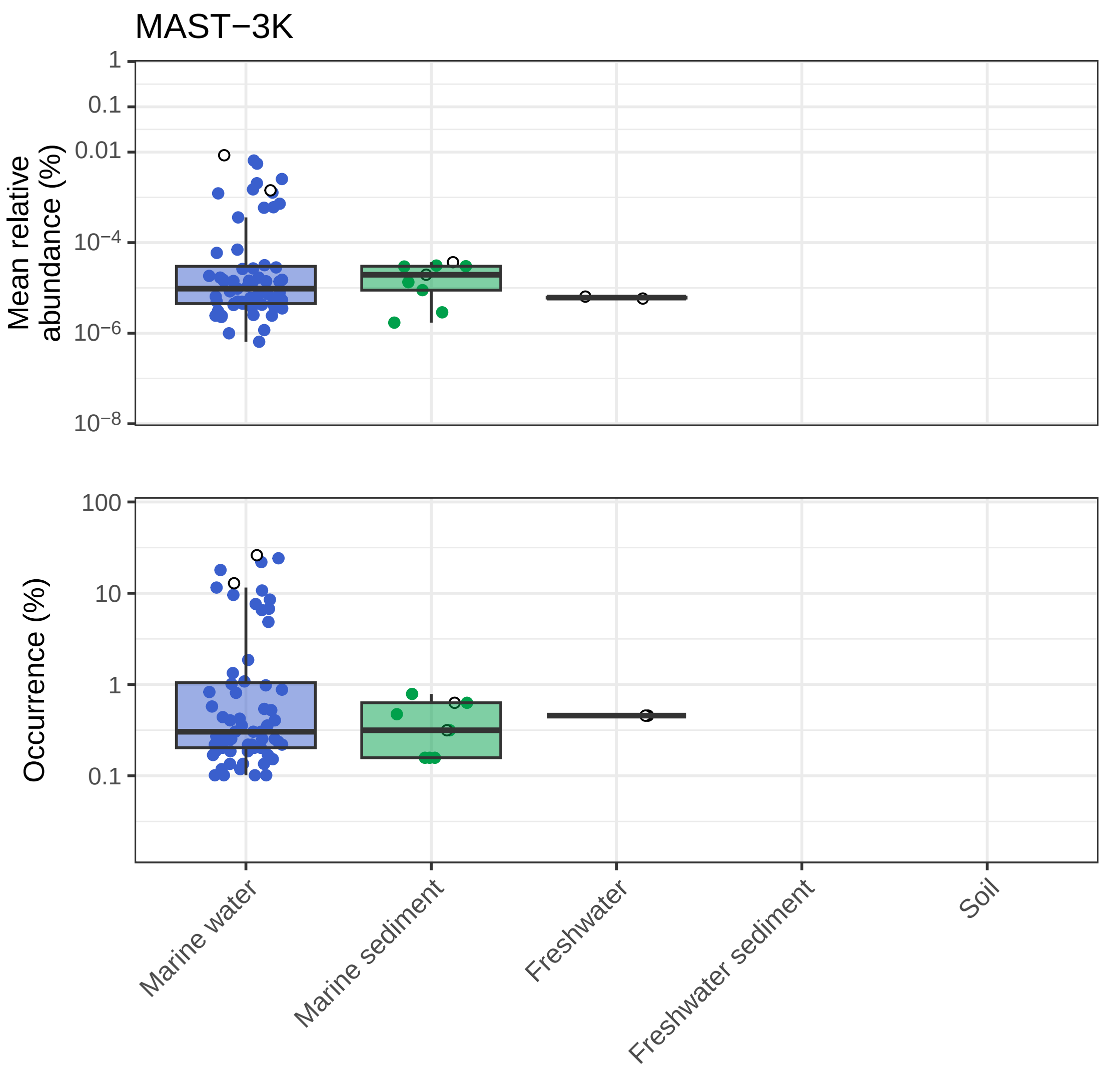

MAST−3K
Mean relative abundance (%)
1
0.1
0.01
10−4
10−6
10−8
Occurrence (%)
100
10
1
0.1
Marine water
Marine sediment
Freshwater
Freshwater sediment
Soil

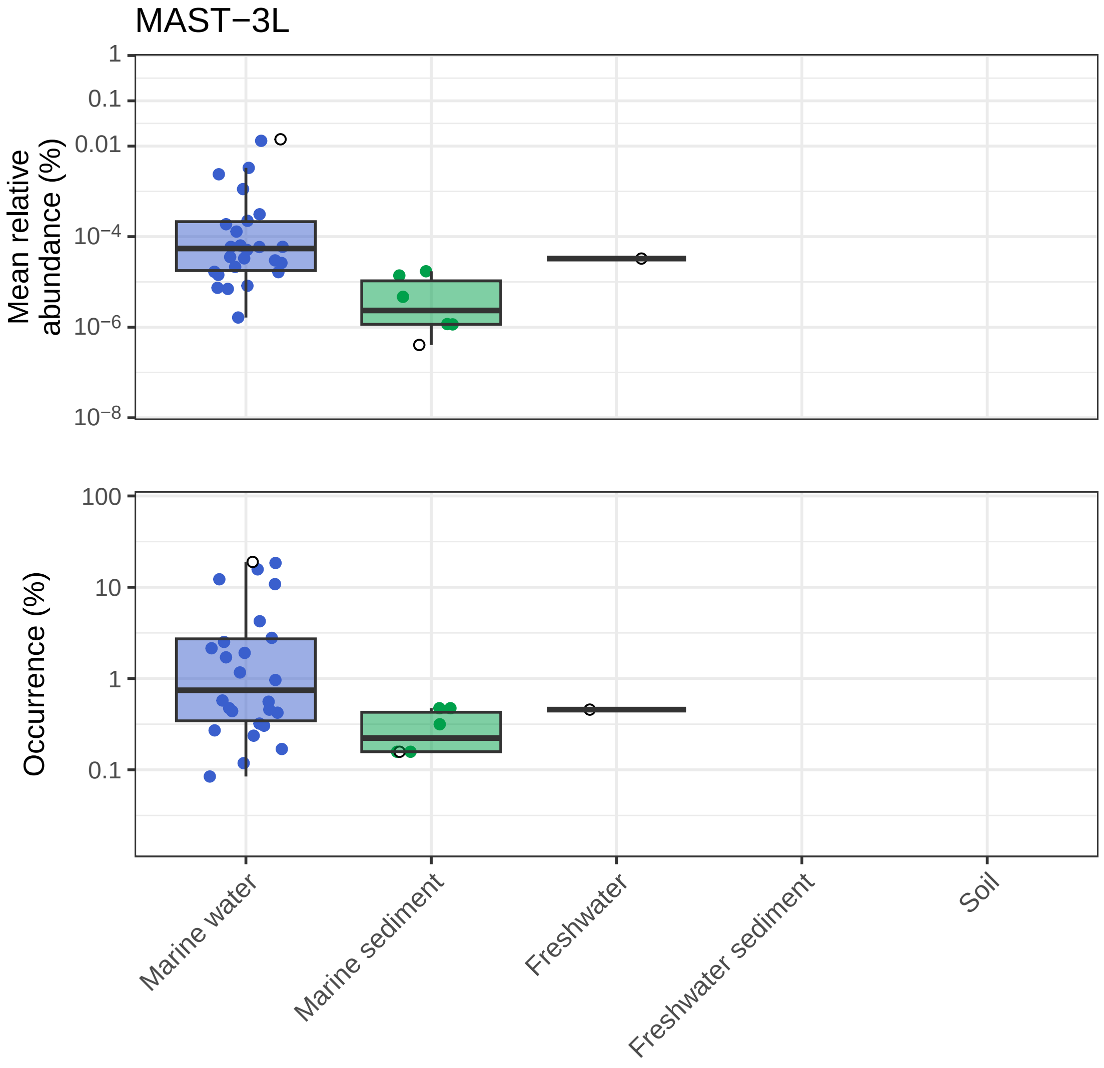
MAST−3L
Mean relative abundance (%)
1
0.1
0.01
10^{-4}
10^{-6}
10^{-8}
Occurrence (%)
100
10
1
0.1
Marine water
Marine sediment
Freshwater
Freshwater sediment
Soil

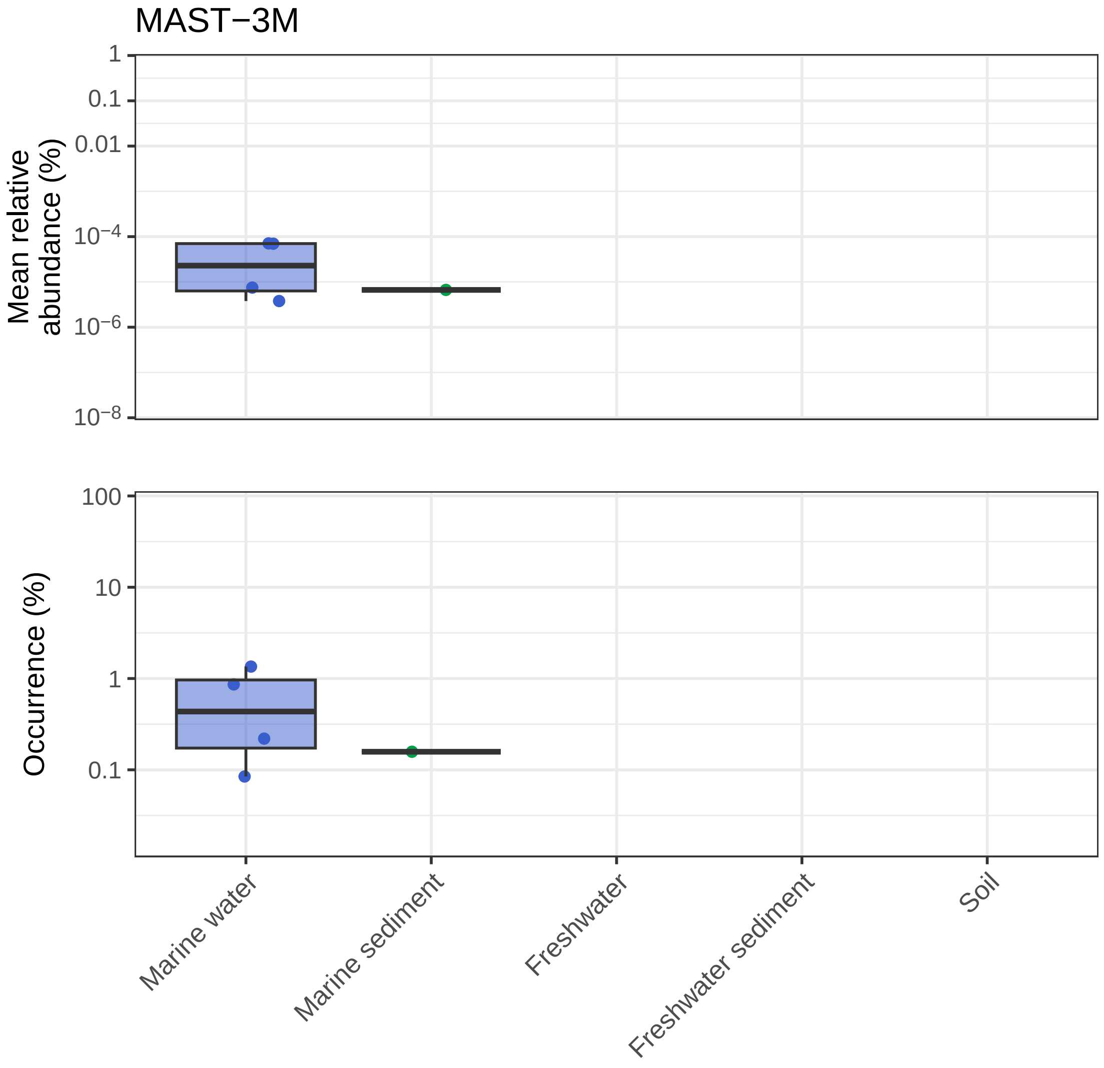

MAST−3M
Mean relative abundance (%)
1
0.1
0.01
10−4
10−6
10−8
Occurrence (%)
100
10
1
0.1
Marine water
Marine sediment
Freshwater
Freshwater sediment
Soil

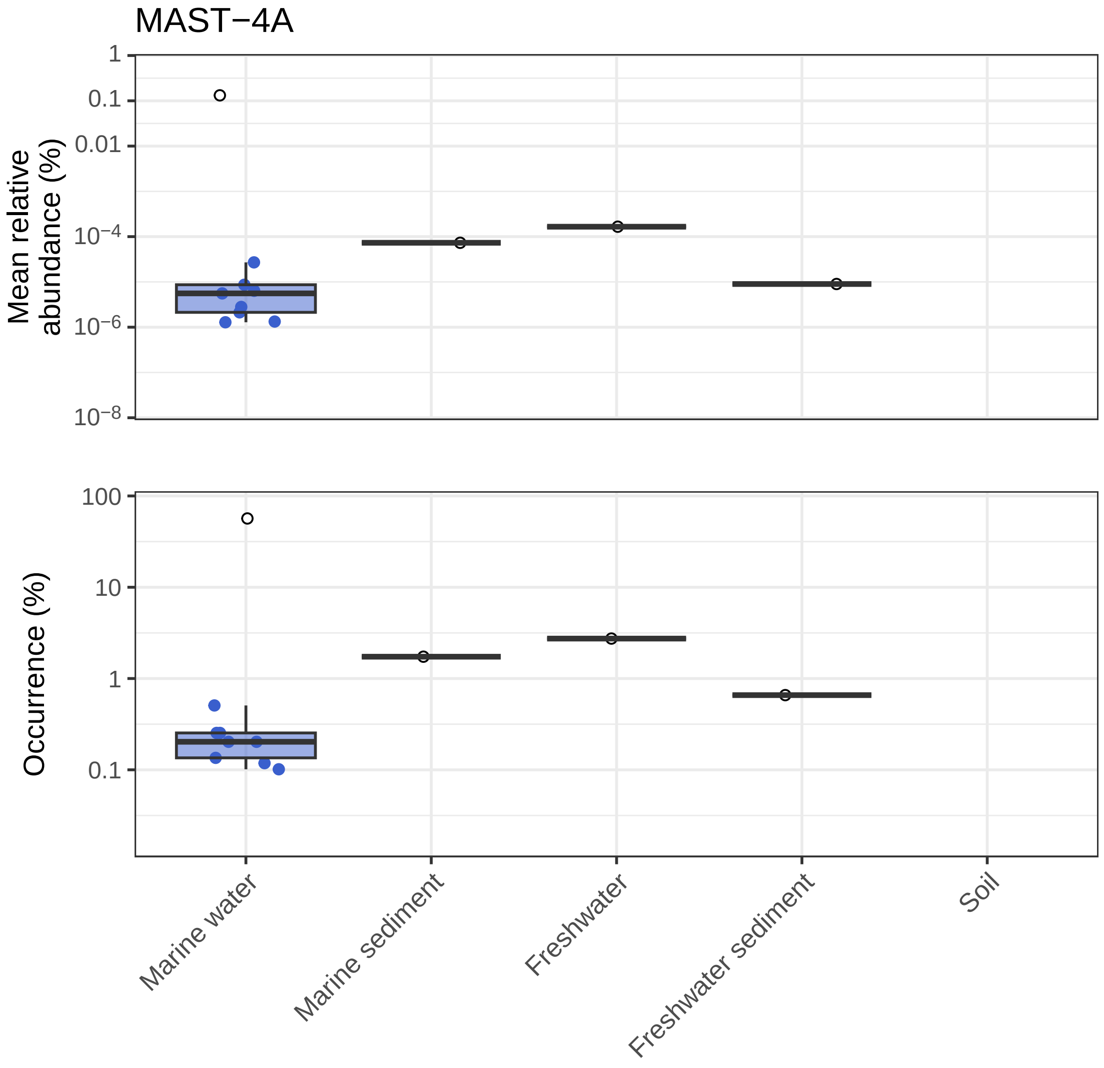
MAST−4A
Mean relative abundance (%)
1
0.1
0.01
10^{-4}
10^{-6}
10^{-8}
Occurrence (%)
100
10
1
0.1
Marine water
Marine sediment
Freshwater
Freshwater sediment
Soil

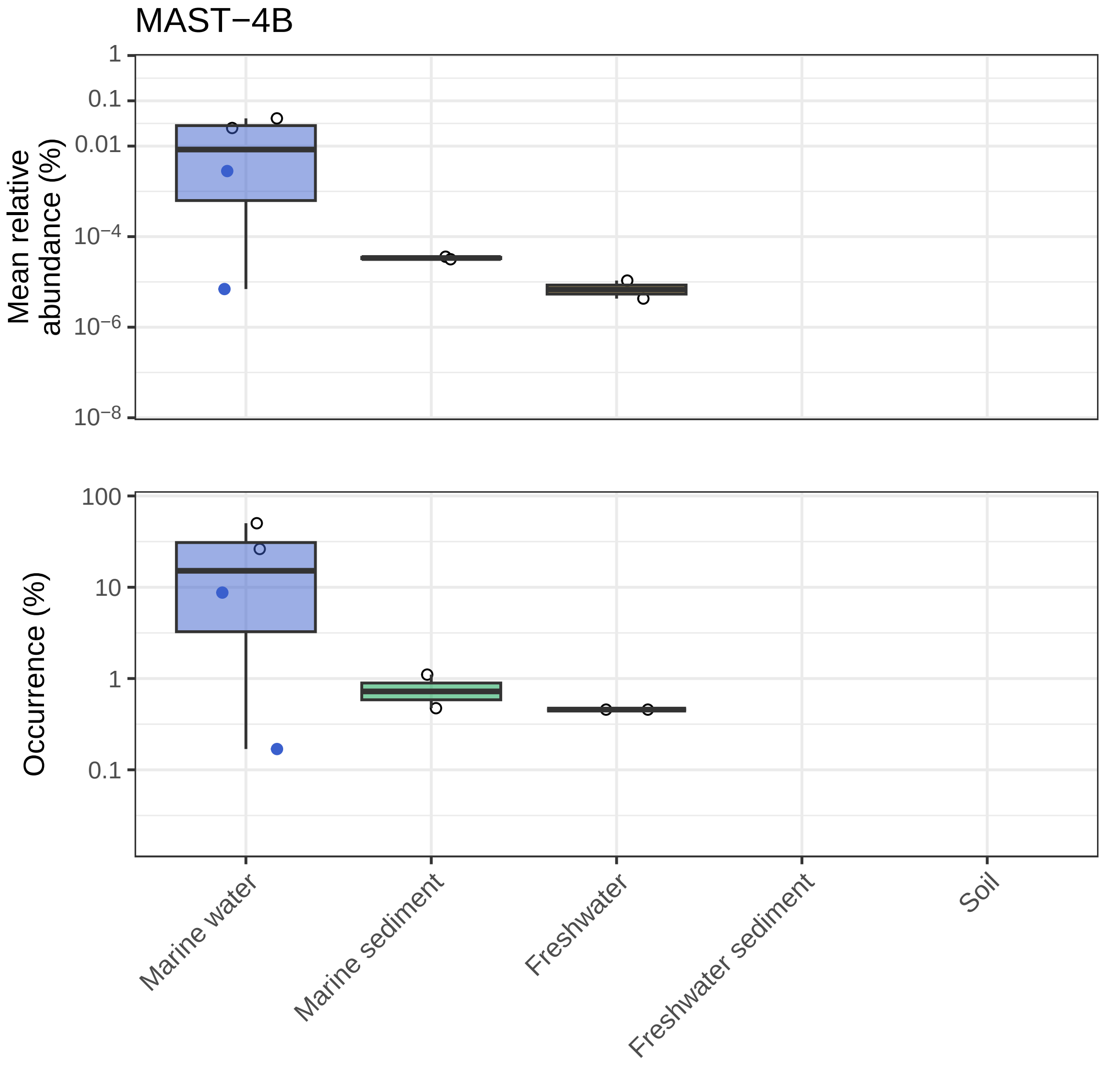

MAST−4B
Mean relative abundance (%)
1
0.1
0.01
10^{-4}
10^{-6}
10^{-8}
Occurrence (%)
100
10
1
0.1
Marine water
Marine sediment
Freshwater
Freshwater sediment
Soil

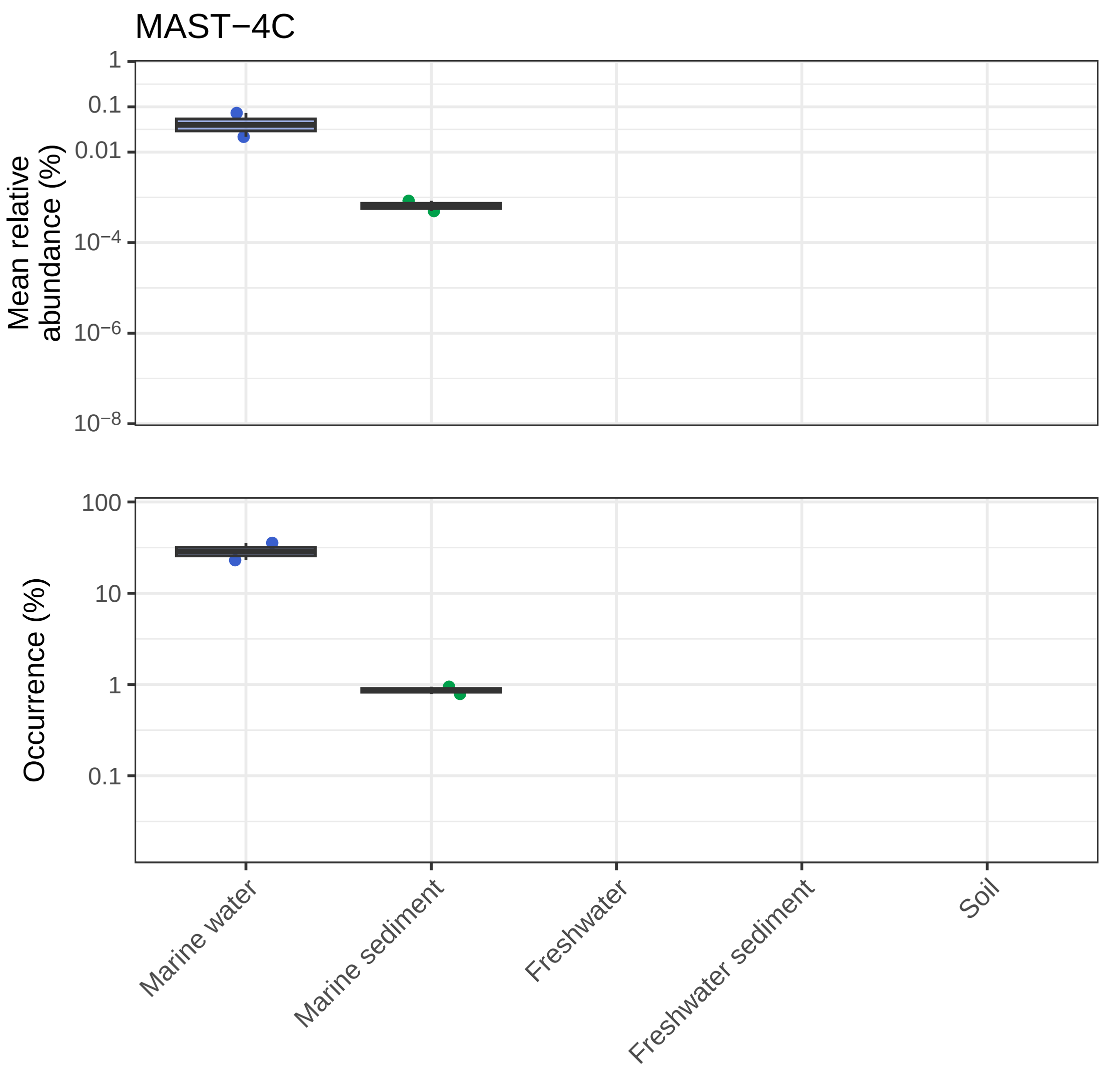
MAST−4C
Mean relative abundance (%)
1
0.1
0.01
10^{-4}
10^{-6}
10^{-8}
Occurrence (%)
100
10
1
0.1
Marine water
Marine sediment
Freshwater
Freshwater sediment
Soil

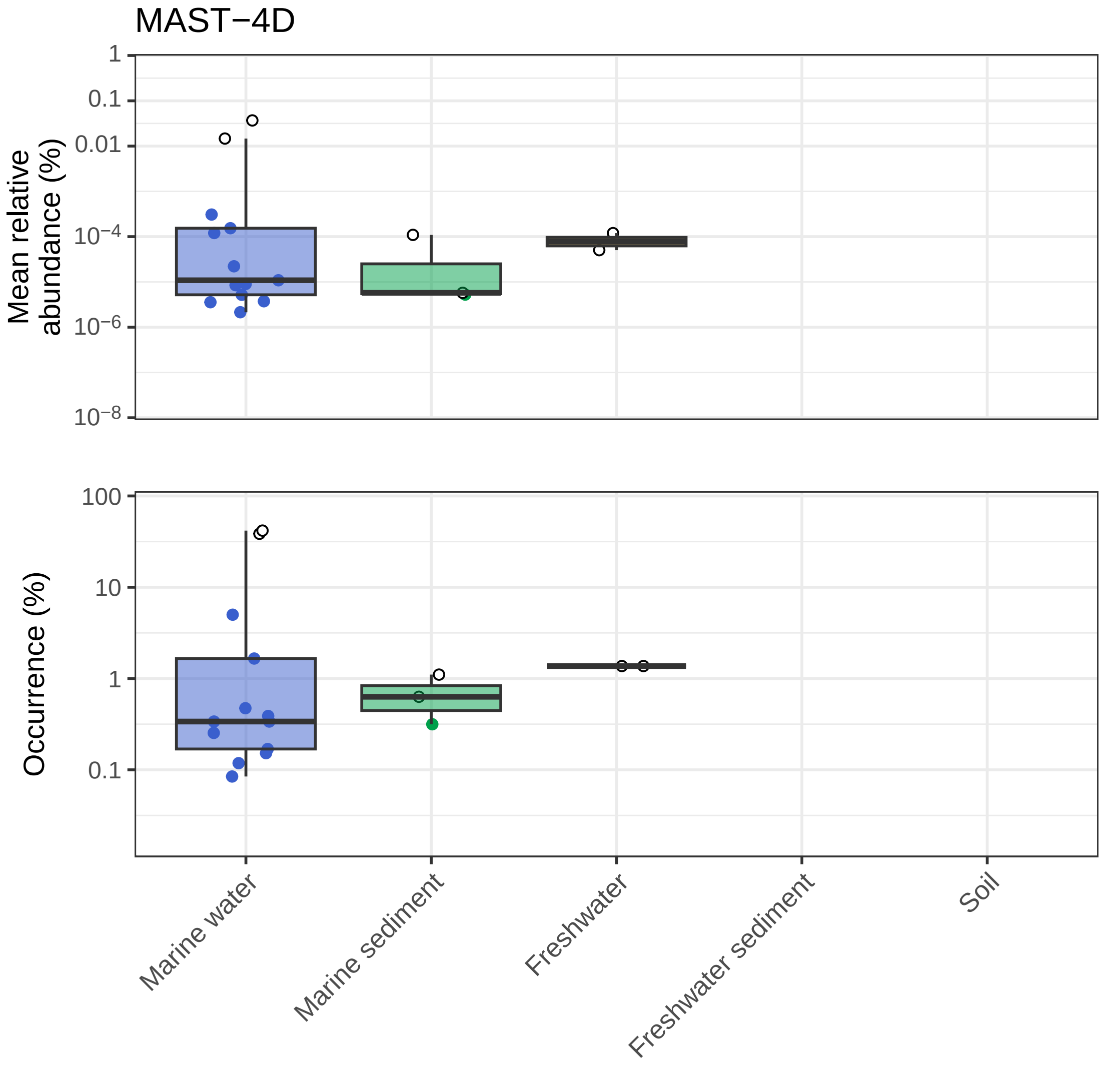

MAST−4D
Mean relative abundance (%)
1
0.1
0.01
10^{-4}
10^{-6}
10^{-8}
Occurrence (%)
100
10
1
0.1
Marine water
Marine sediment
Freshwater
Freshwater sediment
Soil

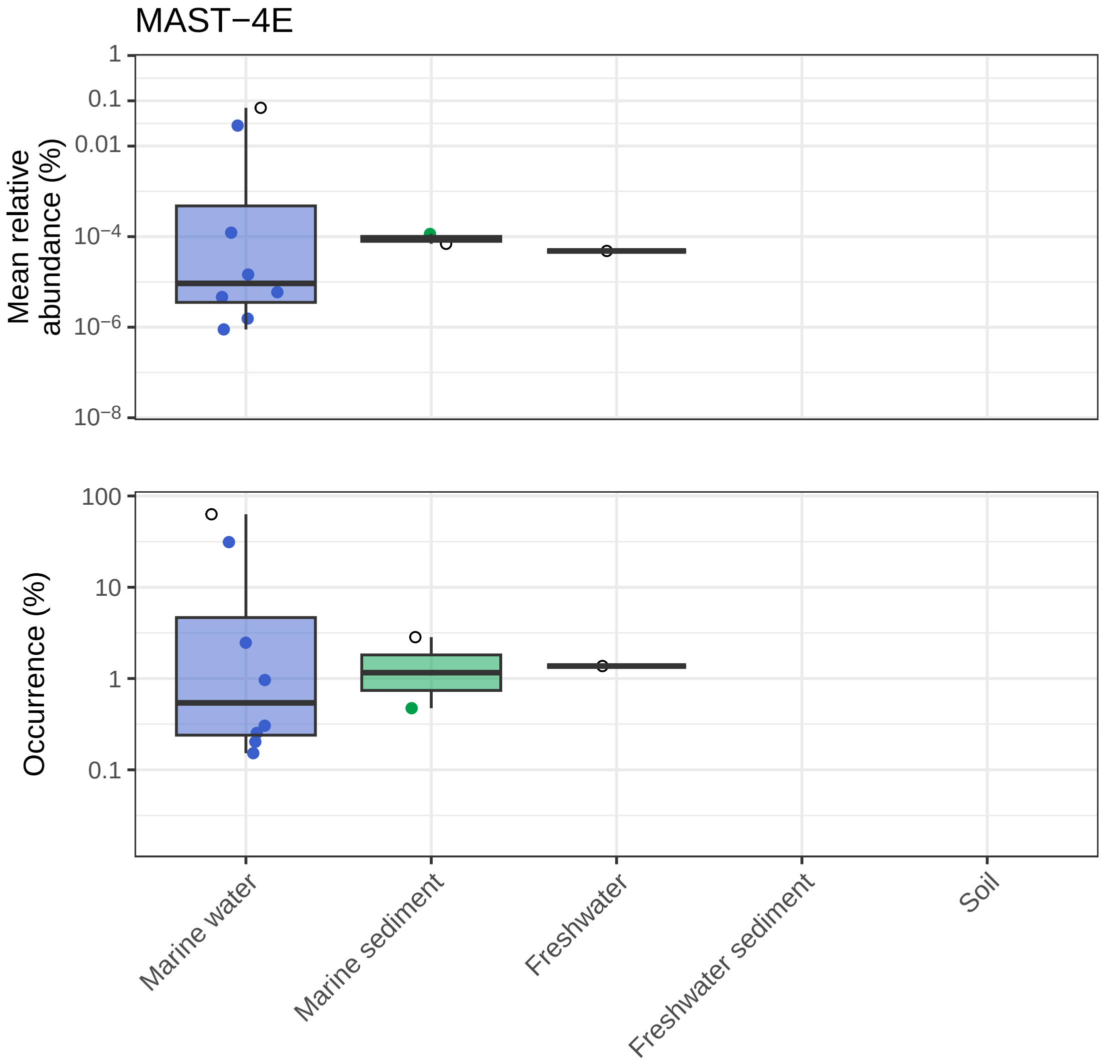
MAST−4E
Mean relative abundance (%)
1
0.1
0.01
10−4
10−6
10−8
Occurrence (%)
100
10
1
0.1
Marine water
Marine sediment
Freshwater
Freshwater sediment
Soil

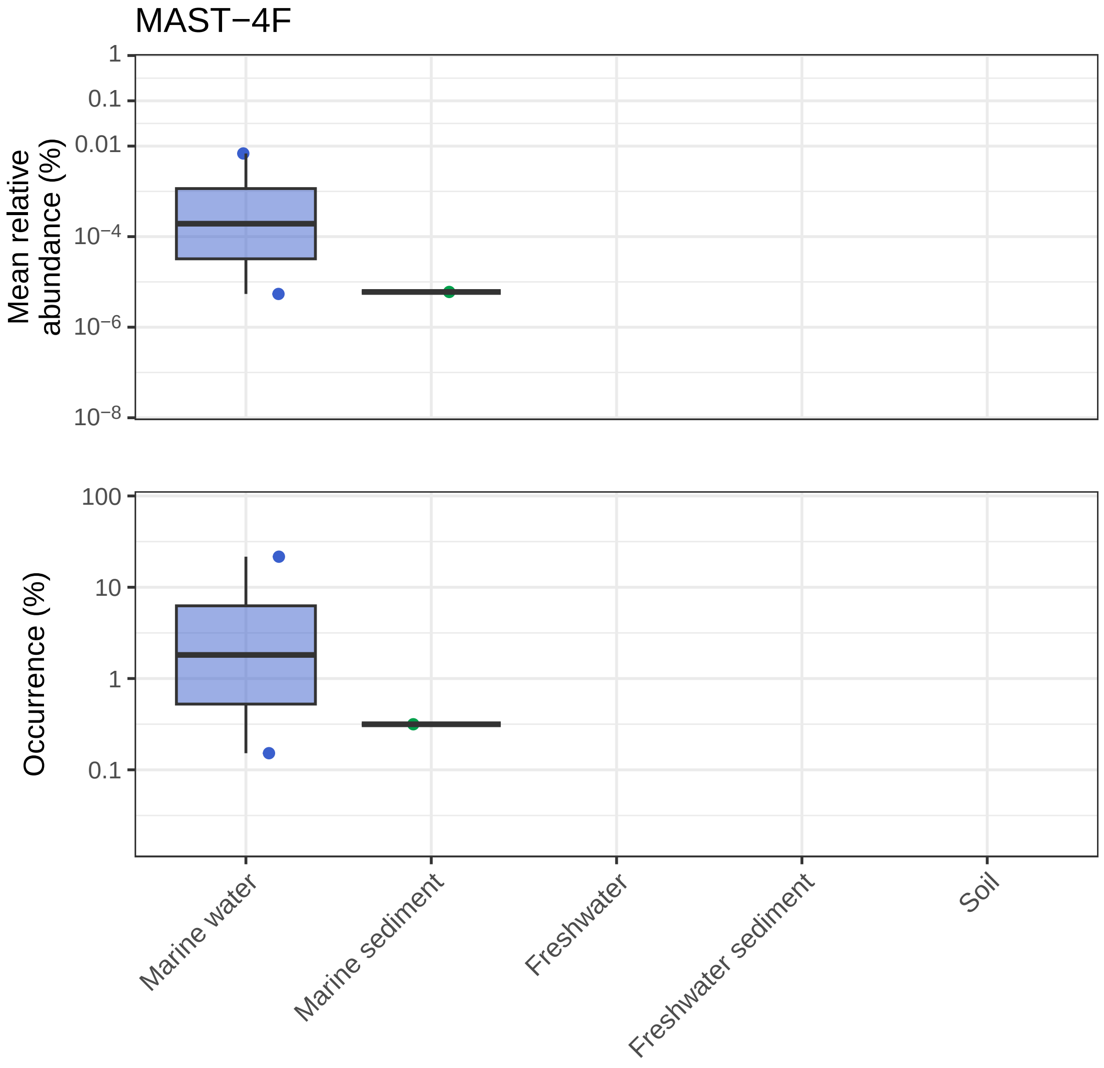

MAST−4F
Mean relative abundance (%)
1
0.1
0.01
10−4
10−6
10−8
Occurrence (%)
100
10
1
0.1
Marine water
Marine sediment
Freshwater
Freshwater sediment
Soil

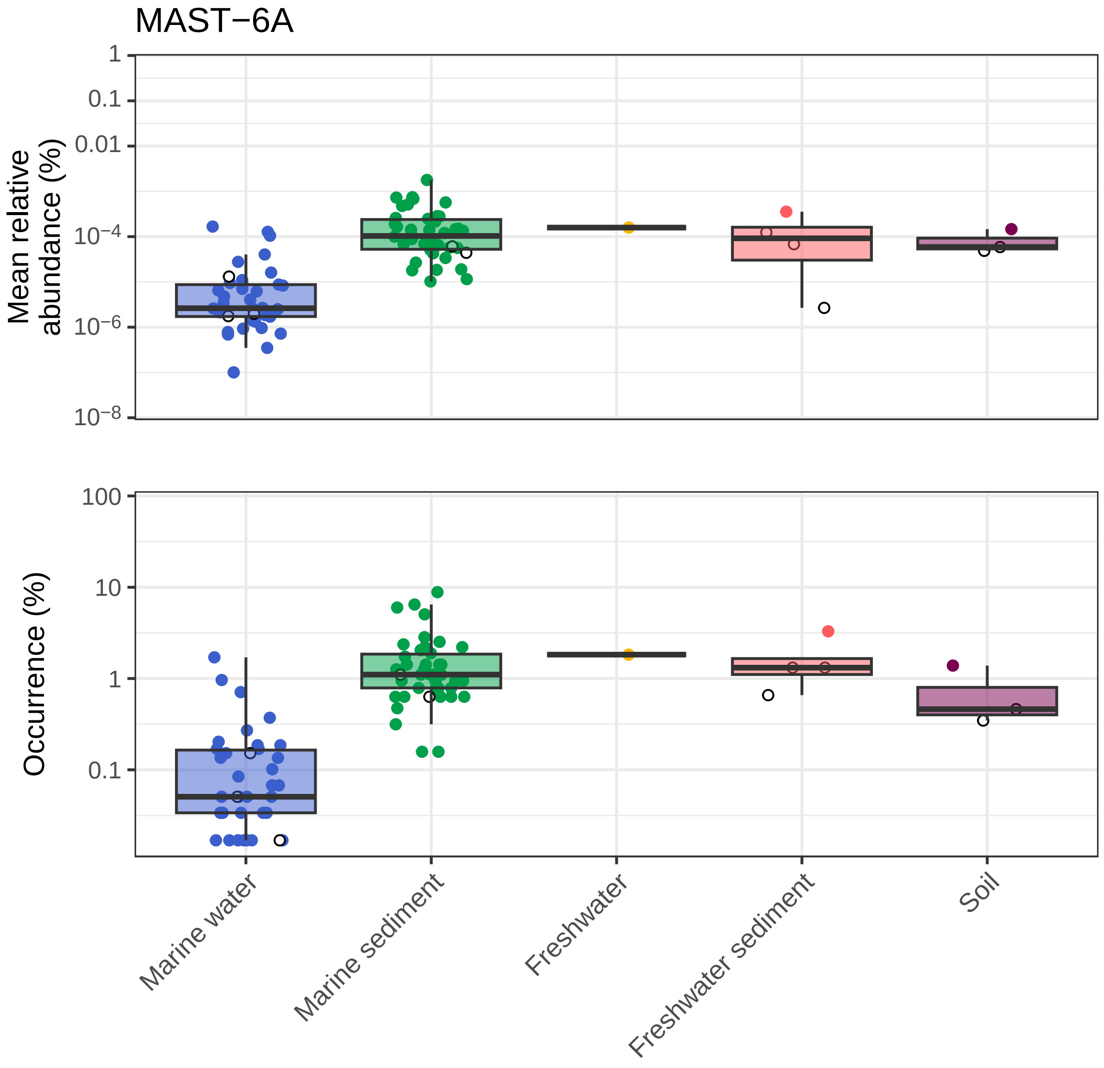

MAST−6A
Mean relative abundance (%)
1
0.1
0.01
10−4
10−6
10−8
Occurrence (%)
100
10
1
0.1
Marine water
Marine sediment
Freshwater
Freshwater sediment
Soil

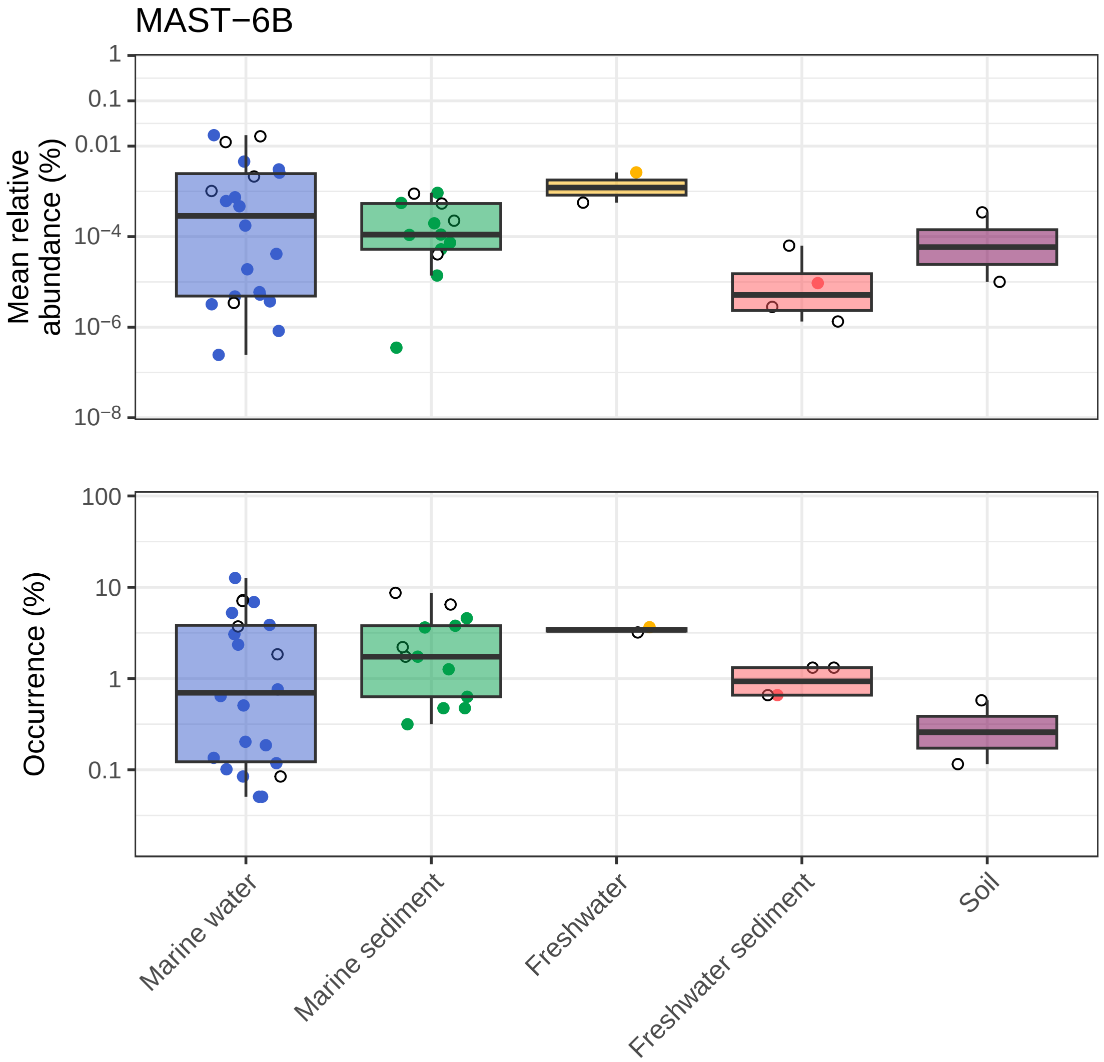
MAST−6B
Mean relative abundance (%)
1
0.1
0.01
10^{-4}
10^{-6}
10^{-8}
Occurrence (%)
100
10
1
0.1
Marine water
Marine sediment
Freshwater
Freshwater sediment
Soil

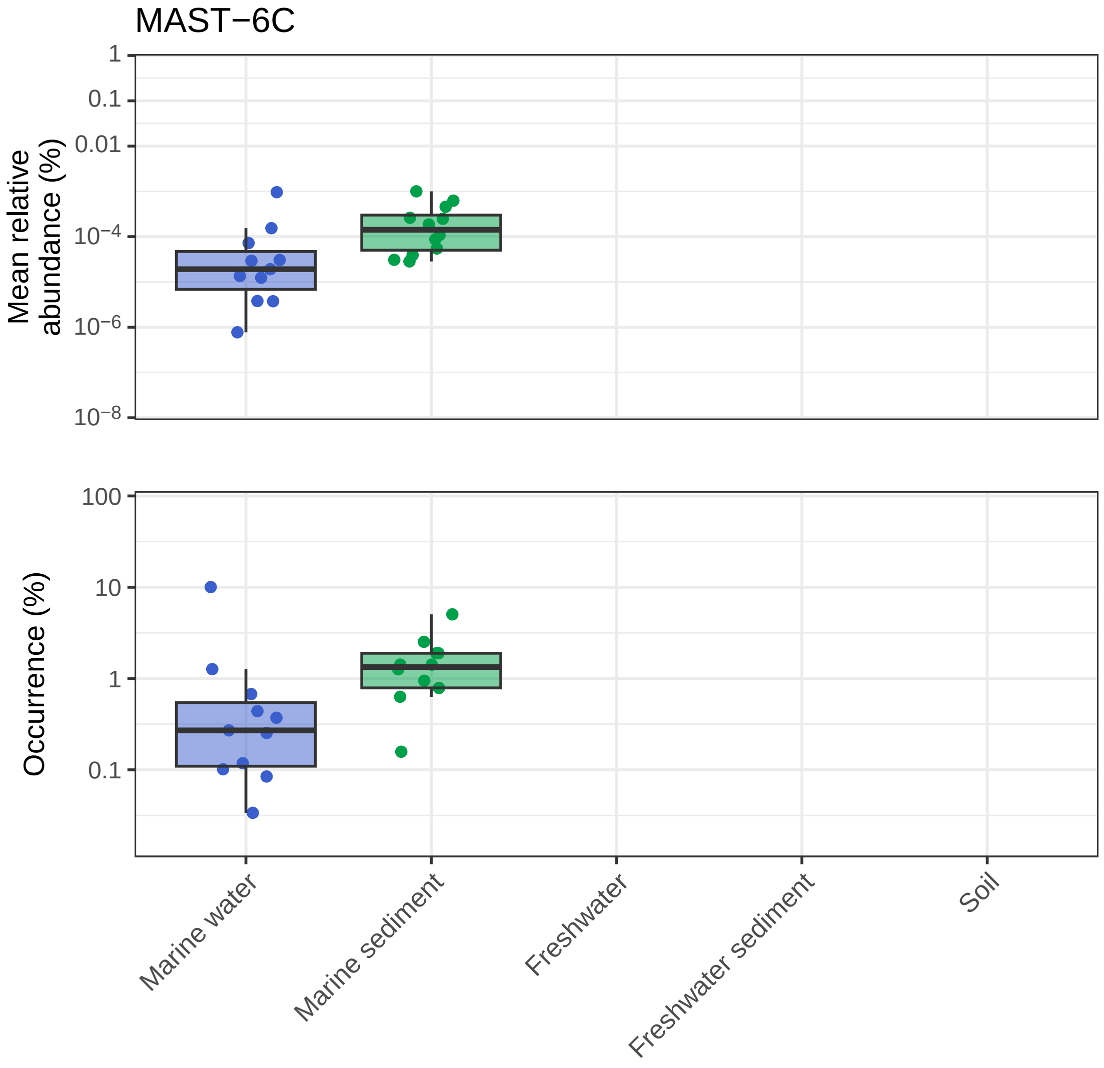

MAST−6C
Mean relative abundance (%)
1
0.1
0.01
10^{-4}
10^{-6}
10^{-8}
Occurrence (%)
100
10
1
0.1
Marine water
Marine sediment
Freshwater
Freshwater sediment
Soil

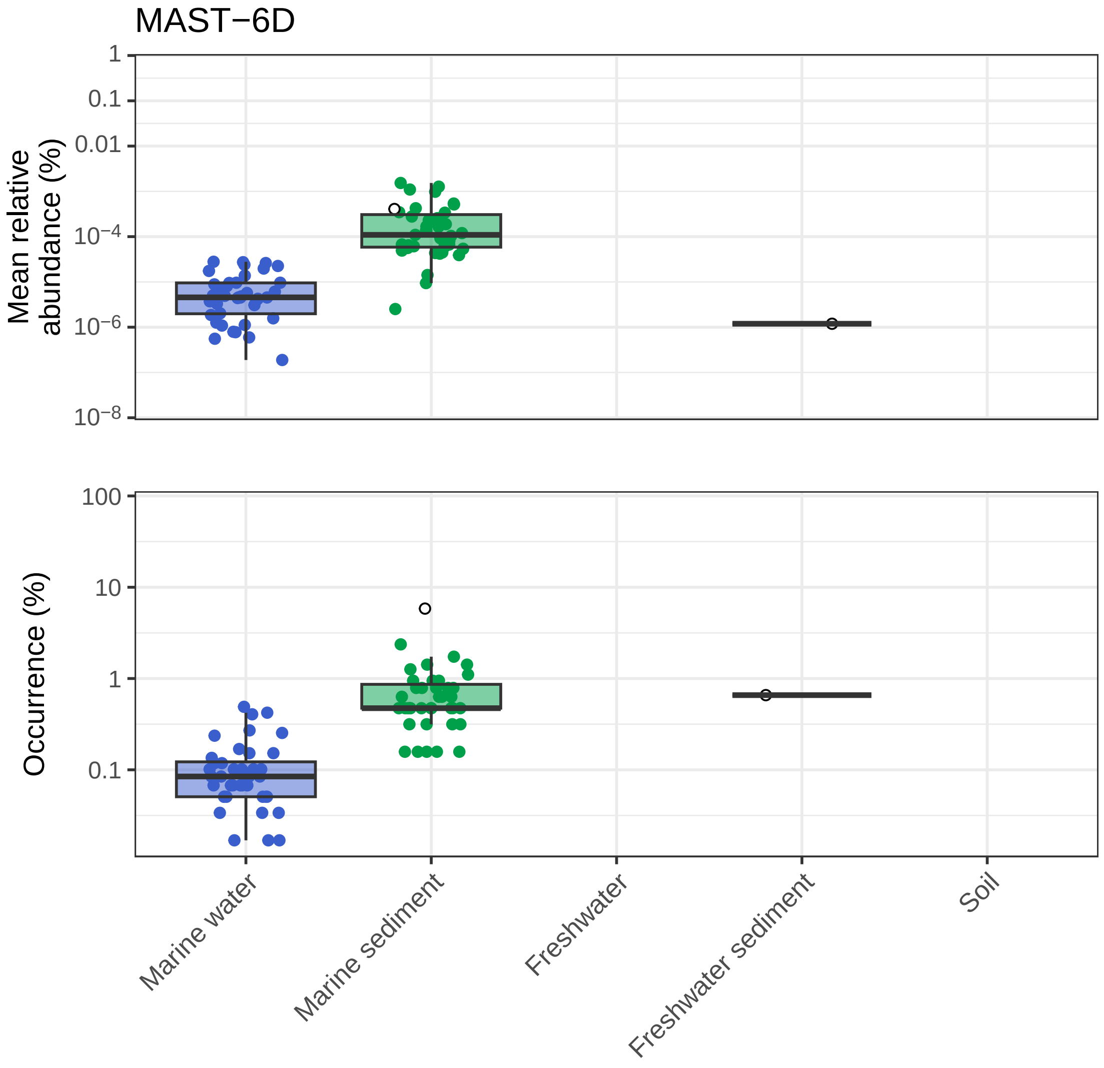
MAST−6D
Mean relative abundance (%)
1
0.1
0.01
10⁻⁴
10⁻⁶
10⁻⁸
Occurrence (%)
100
10
1
0.1
Marine water
Marine sediment
Freshwater
Freshwater sediment
Soil

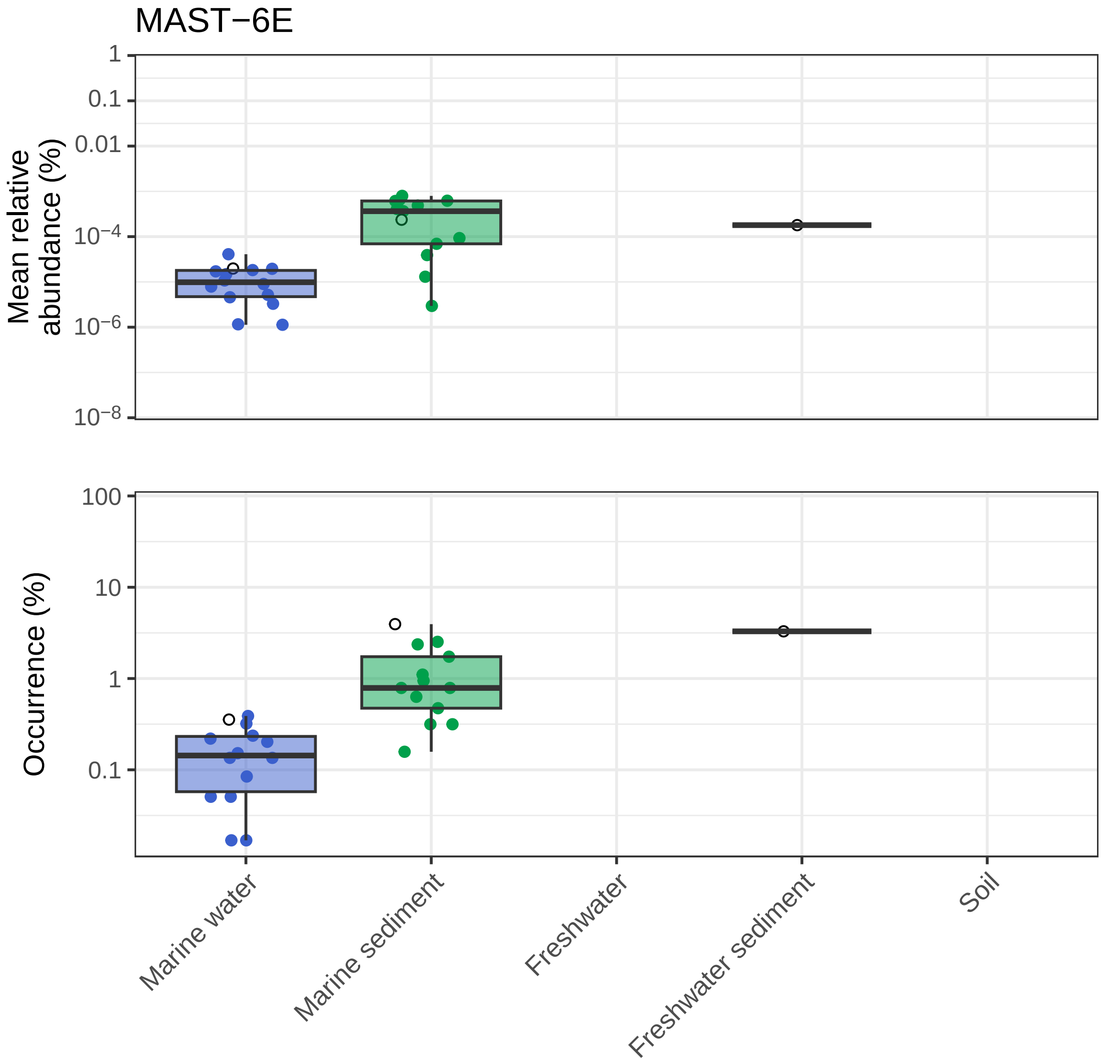

MAST−6E
Mean relative abundance (%)
1
0.1
0.01
10^{-4}
10^{-6}
10^{-8}
Occurrence (%)
100
10
1
0.1
Marine water
Marine sediment
Freshwater
Freshwater sediment
Soil

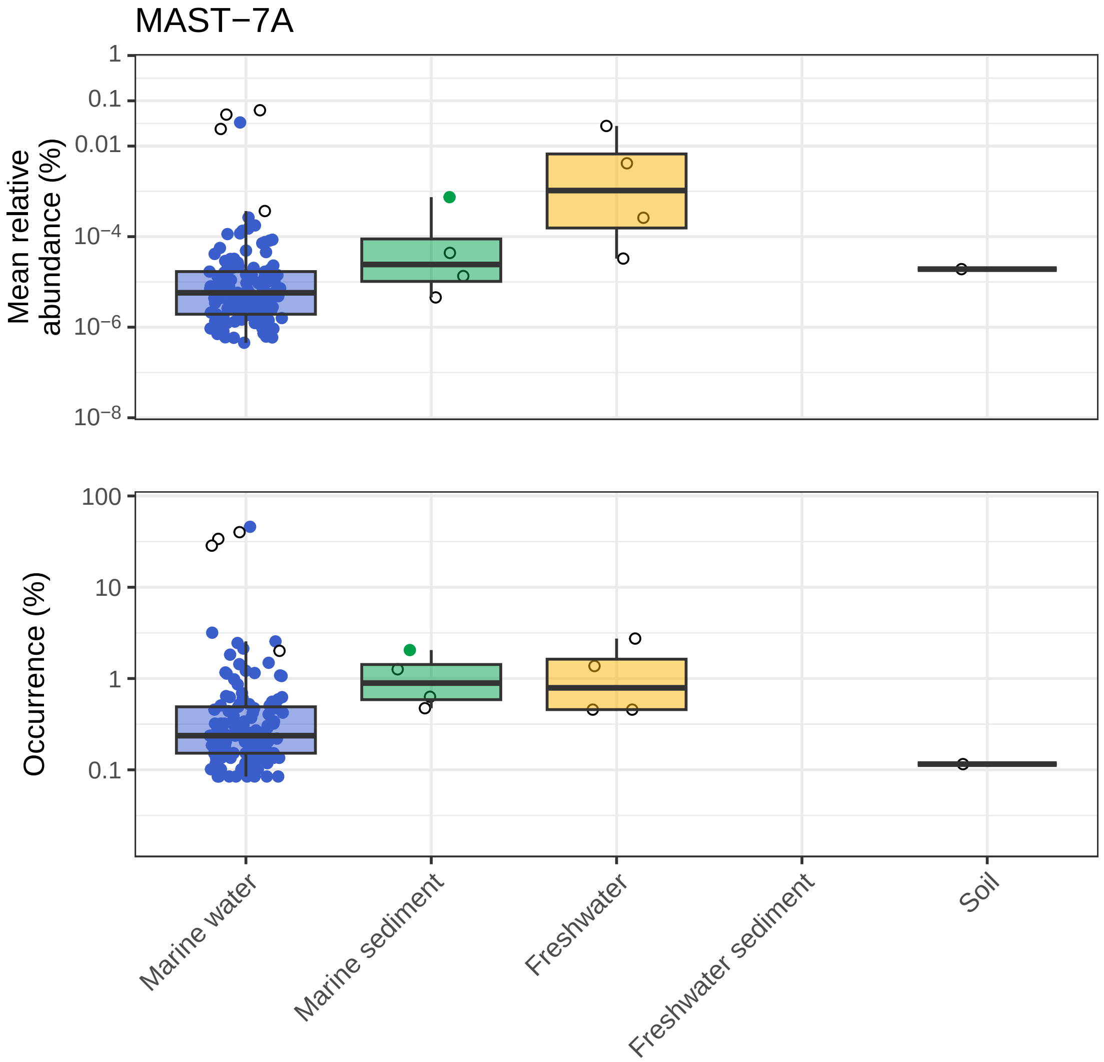
MAST−7A
Mean relative abundance (%)
1
0.1
0.01
10−4
10−6
10−8
Occurrence (%)
100
10
1
0.1
Marine water
Marine sediment
Freshwater
Freshwater sediment
Soil

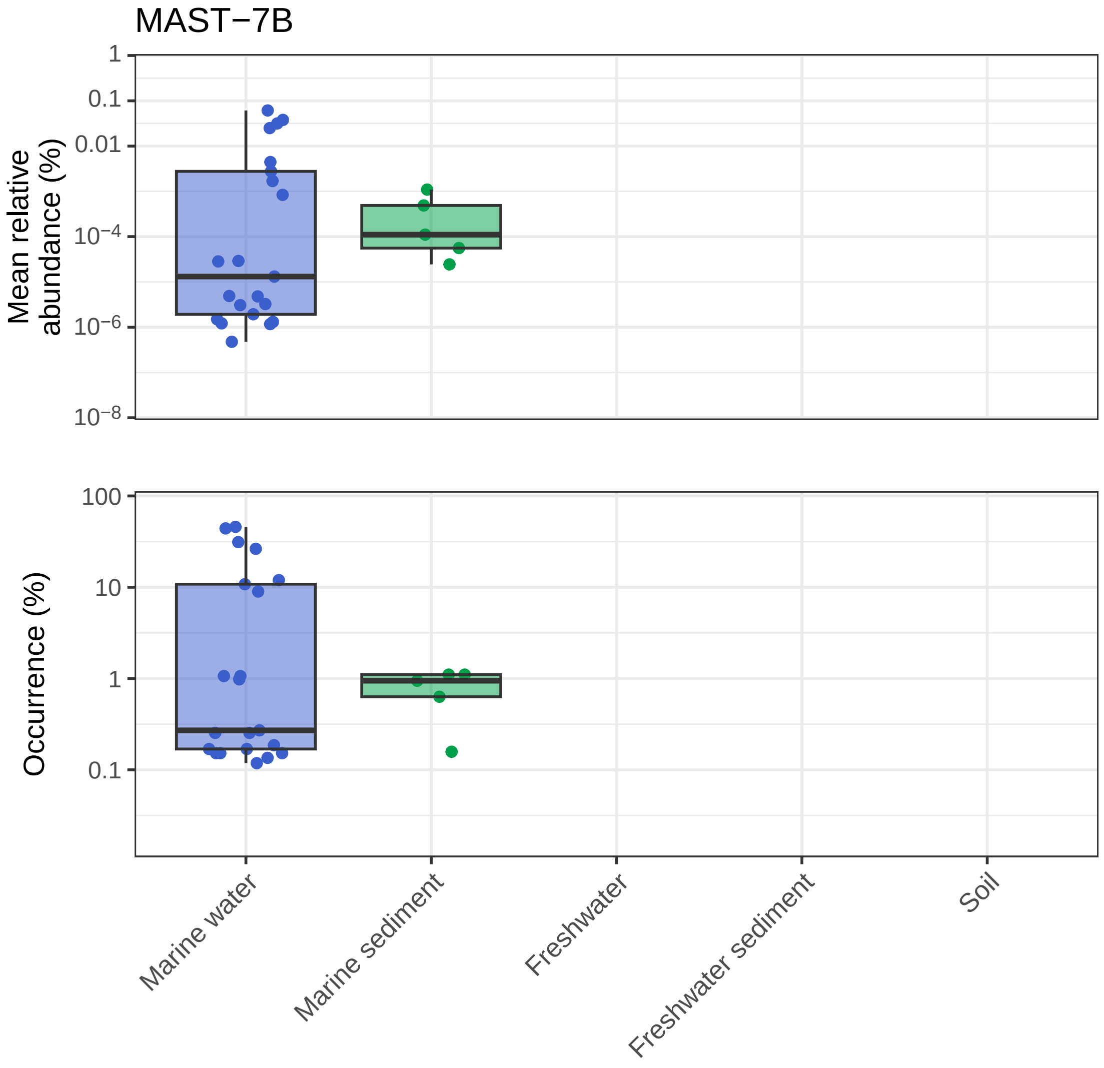
MAST−7B
Mean relative abundance (%)
1
0.1
0.01
10^{-4}
10^{-6}
10^{-8}
Occurrence (%)
100
10
1
0.1
Marine water
Marine sediment
Freshwater
Freshwater sediment
Soil

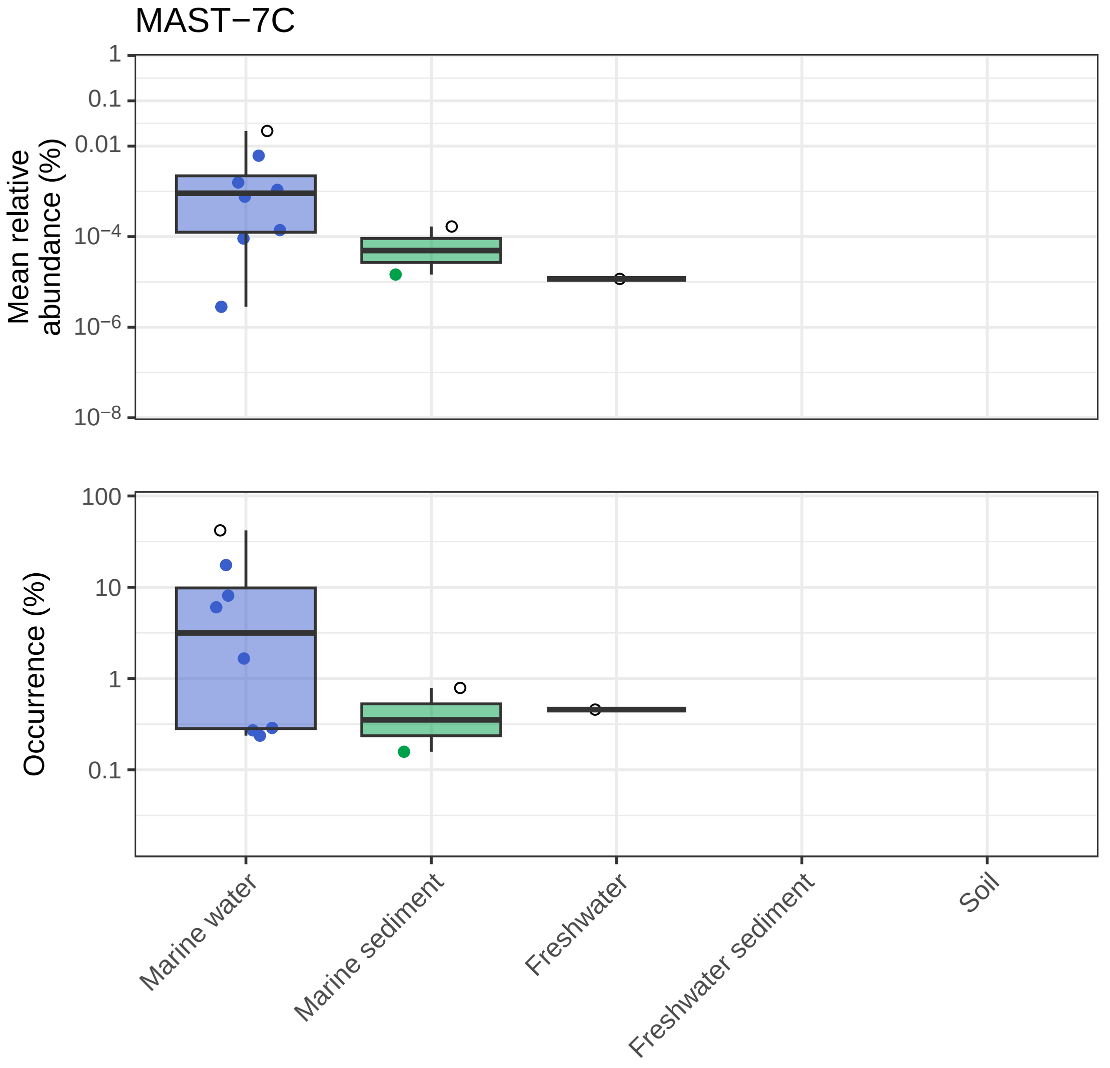

MAST−7C
Mean relative abundance (%)
1
0.1
0.01
10^{-4}
10^{-6}
10^{-8}
Occurrence (%)
100
10
1
0.1
Marine water
Marine sediment
Freshwater
Freshwater sediment
Soil

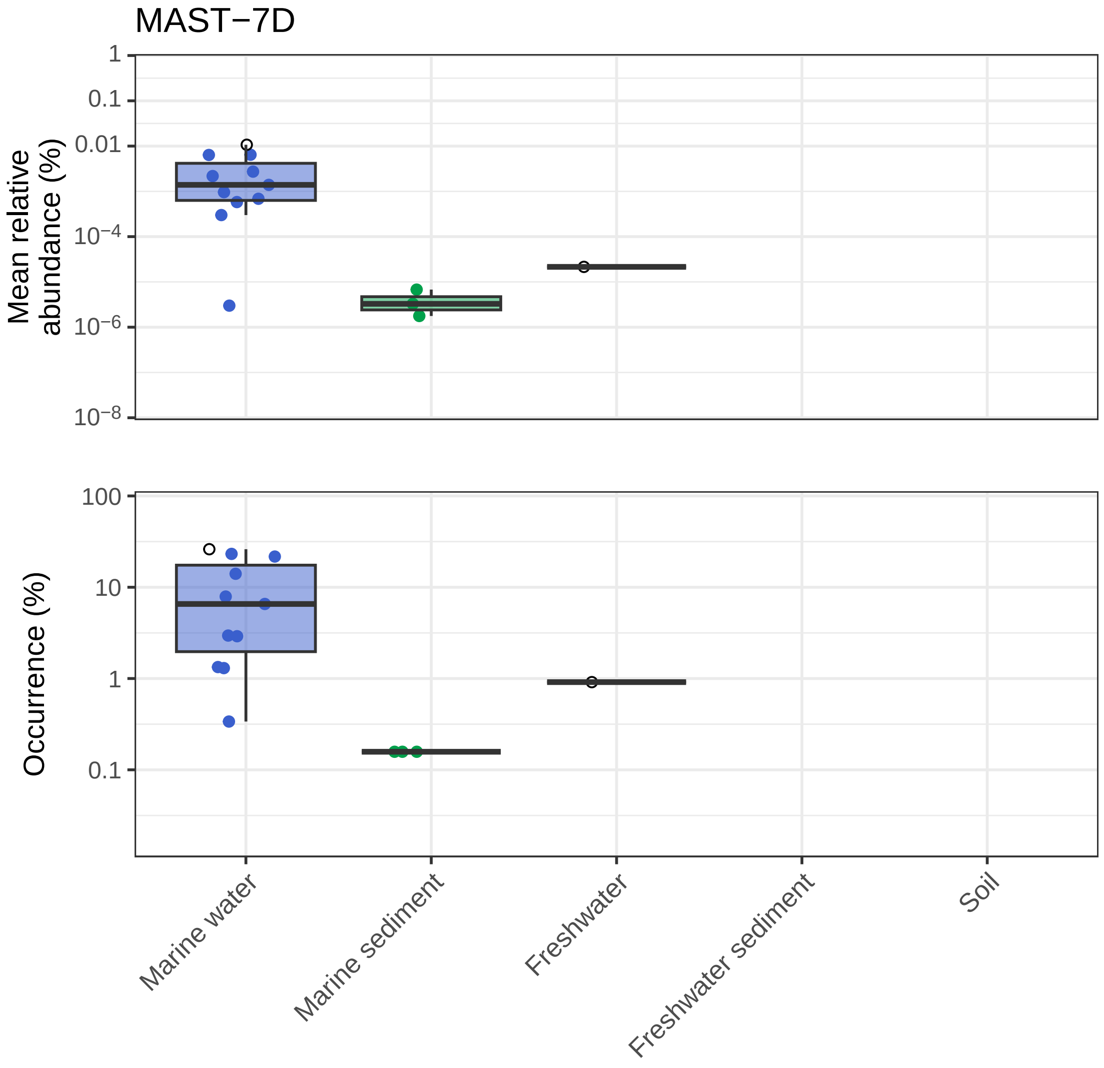

MAST−7D
Mean relative abundance (%)
1
0.1
0.01
10−4
10−6
10−8
Occurrence (%)
100
10
1
0.1
Marine water
Marine sediment
Freshwater
Freshwater sediment
Soil

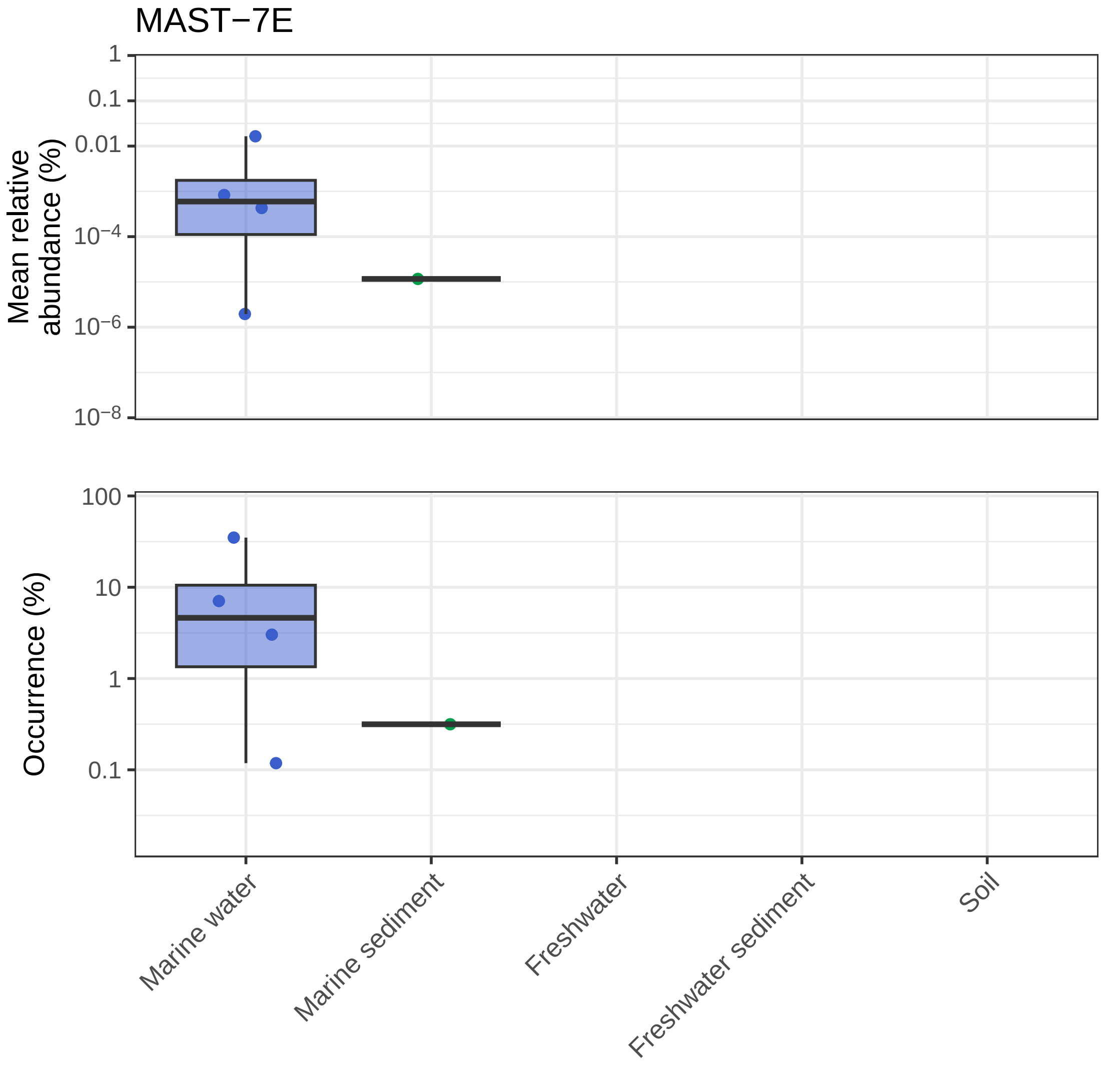

MAST−7E
Mean relative abundance (%)
1
0.1
0.01
10^{-4}
10^{-6}
10^{-8}
Occurrence (%)
100
10
1
0.1
Marine water
Marine sediment
Freshwater
Freshwater sediment
Soil

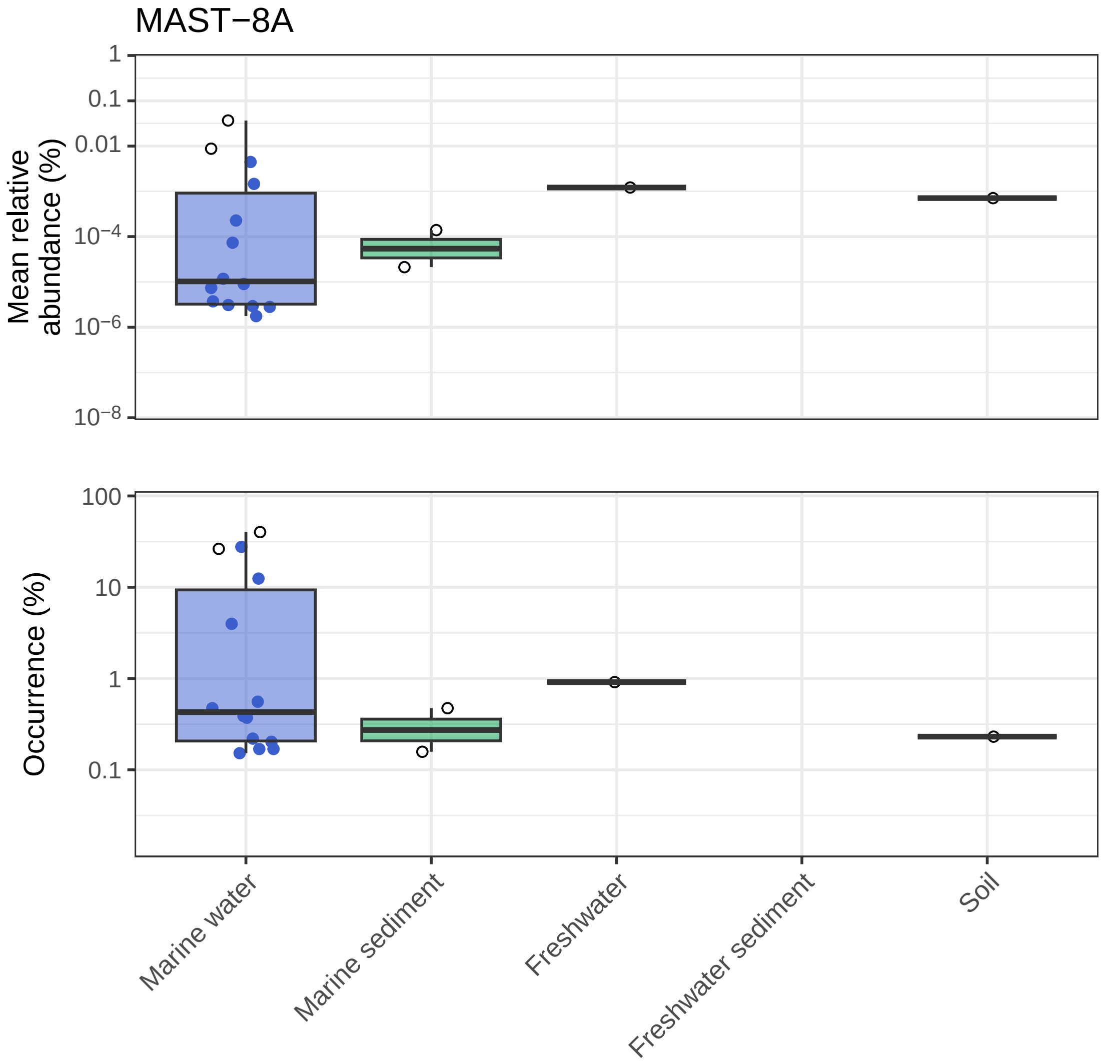

MAST−8A
1
0.1
0.01
10^{-4}
10^{-6}
10^{-8}
Mean relative abundance (%)
100
10
1
0.1
Occurrence (%)
Marine water
Marine sediment
Freshwater
Freshwater sediment
Soil

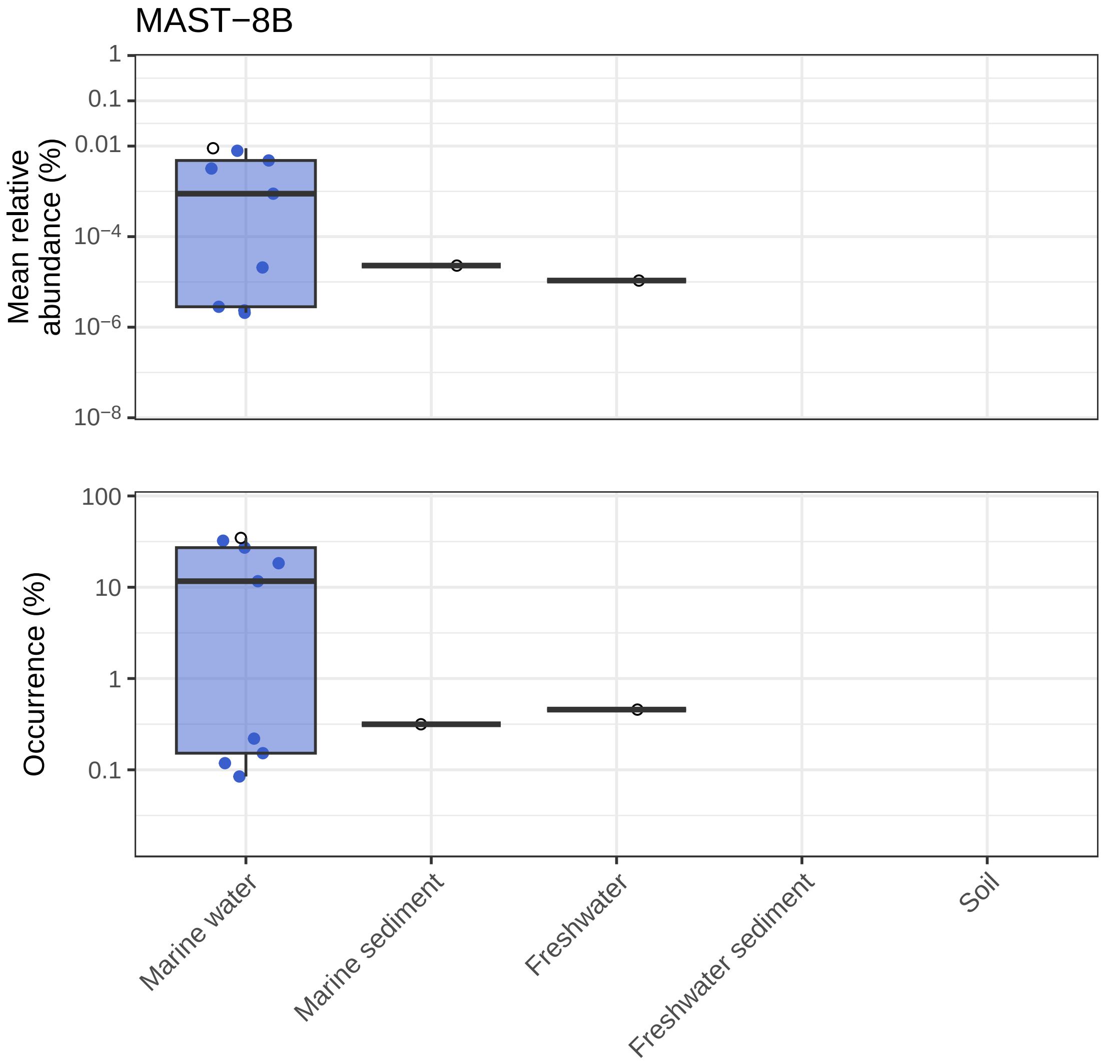
MAST−8B
Mean relative abundance (%)
1
0.1
0.01
10^{-4}
10^{-6}
10^{-8}
Occurrence (%)
100
10
1
0.1
Marine water
Marine sediment
Freshwater
Freshwater sediment
Soil

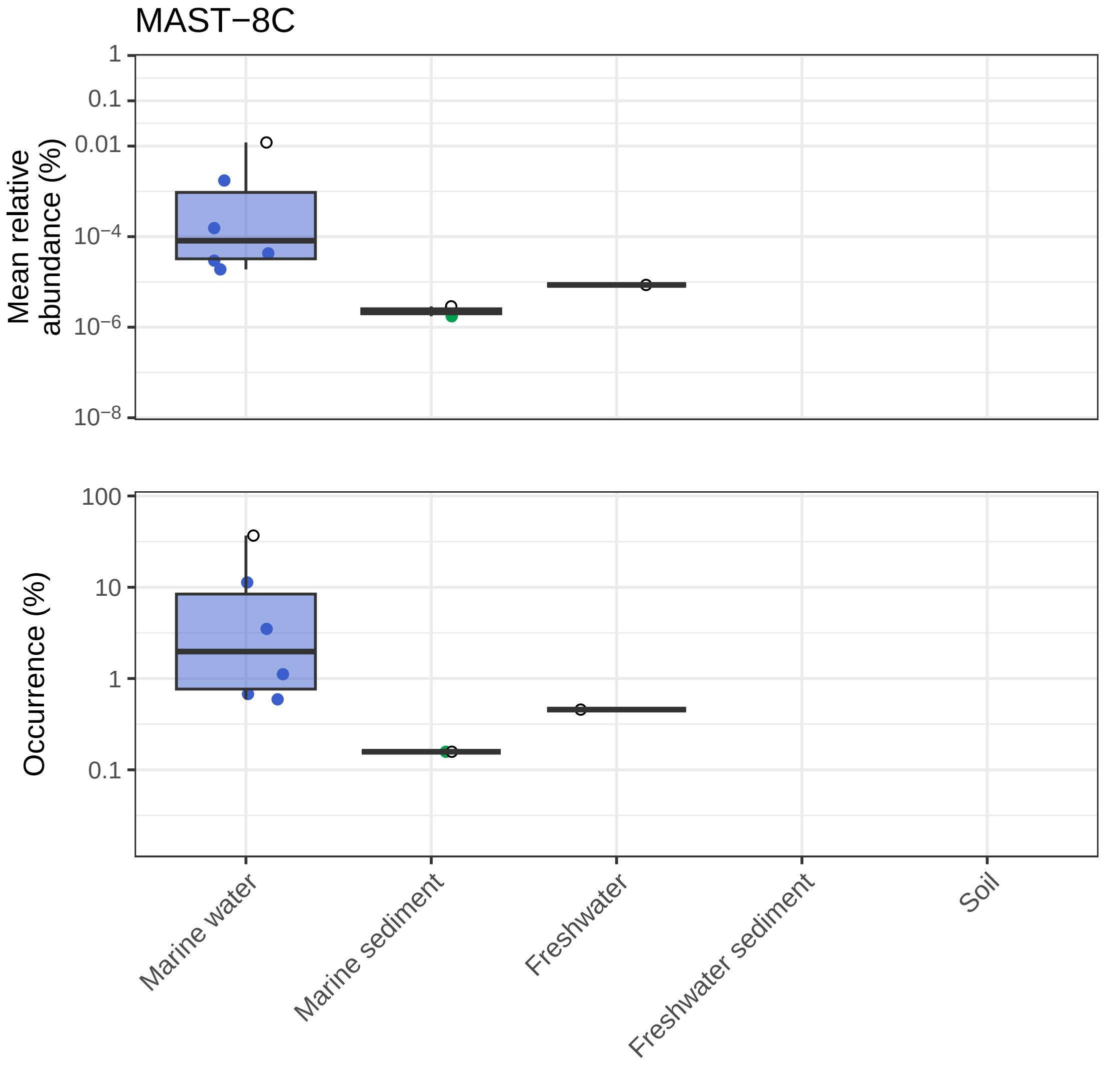
MAST−8C
Mean relative abundance (%)
1
0.1
0.01
10^{-4}
10^{-6}
10^{-8}
Occurrence (%)
100
10
1
0.1
Marine water
Marine sediment
Freshwater
Freshwater sediment
Soil

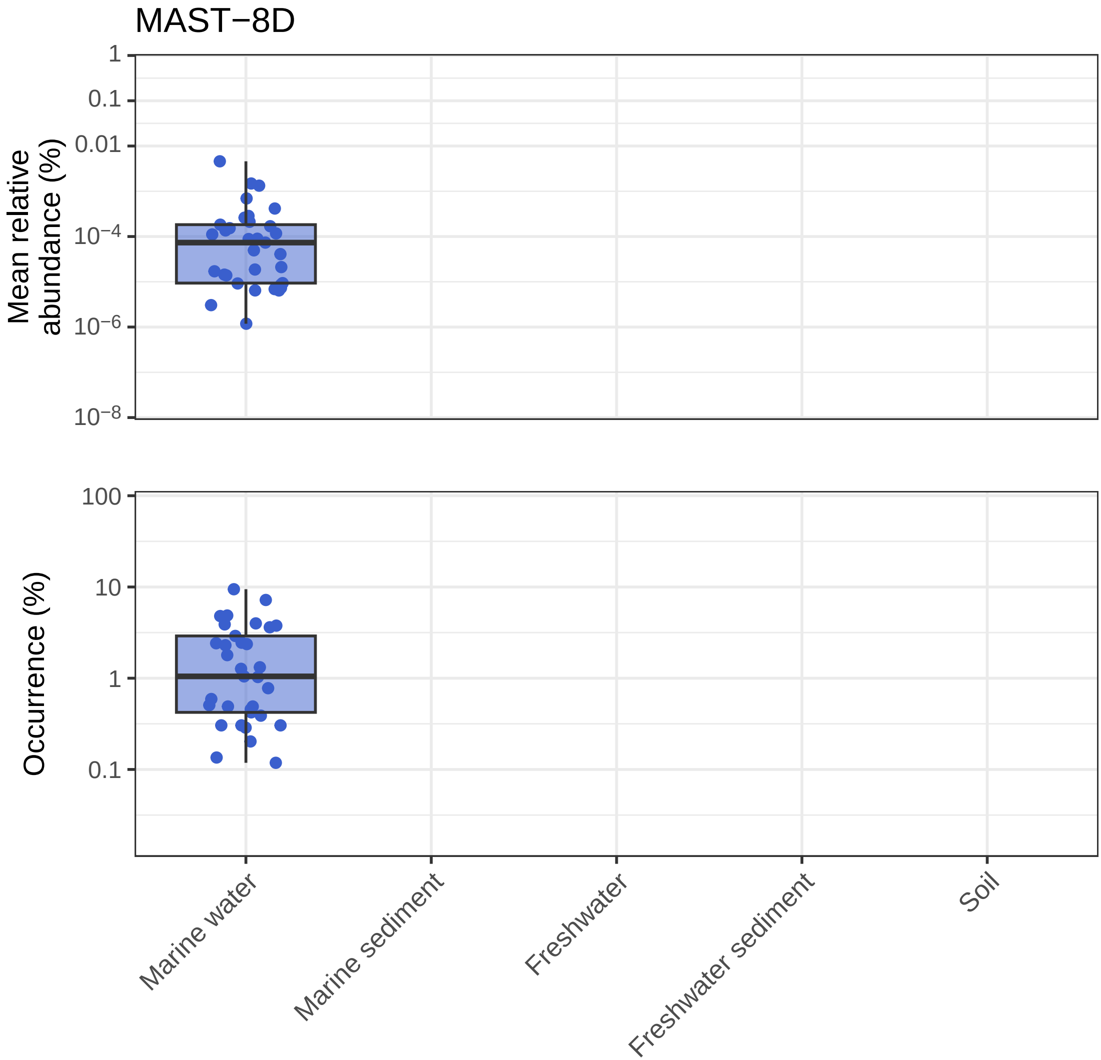
MAST−8D
Mean relative abundance (%)
1
0.1
0.01
10−4
10−6
10−8
Occurrence (%)
100
10
1
0.1
Marine water
Marine sediment
Freshwater
Freshwater sediment
Soil

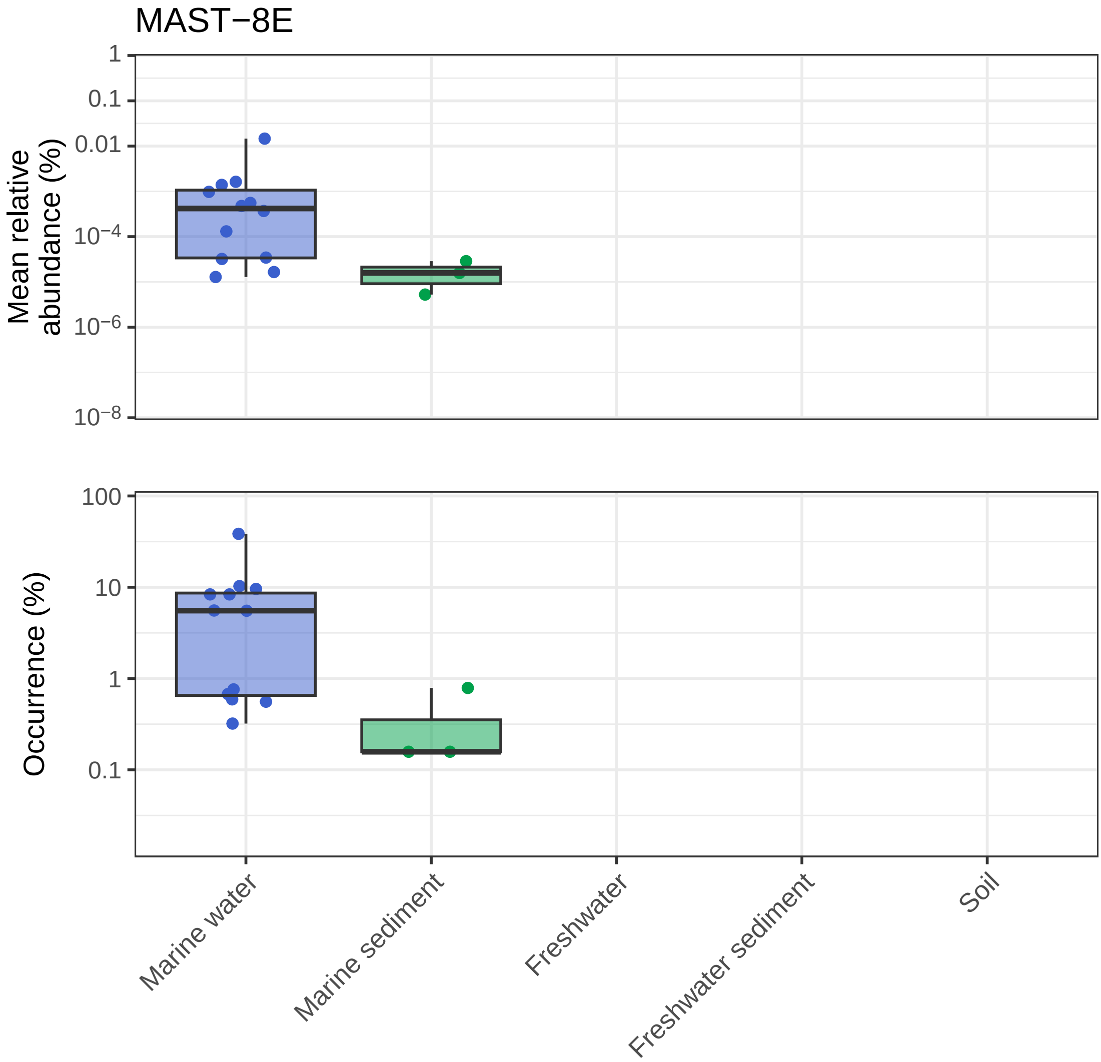

MAST−8E
Mean relative abundance (%)
1
0.1
0.01
10^{-4}
10^{-6}
10^{-8}
Occurrence (%)
100
10
1
0.1
Marine water
Marine sediment
Freshwater
Freshwater sediment
Soil

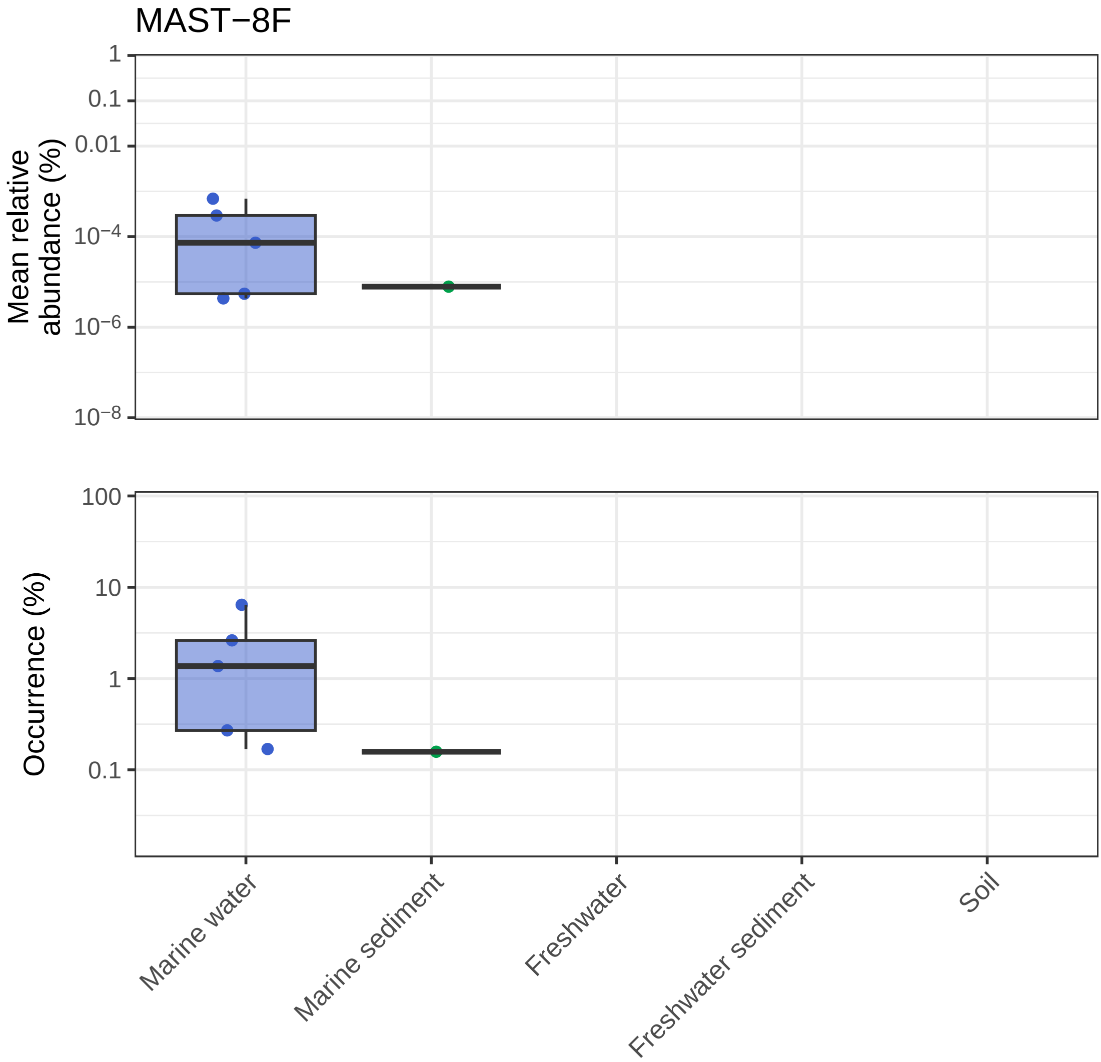
MAST−8F
Mean relative abundance (%)
1
0.1
0.01
10−4
10−6
10−8
Occurrence (%)
100
10
1
0.1
Marine water
Marine sediment
Freshwater
Freshwater sediment
Soil

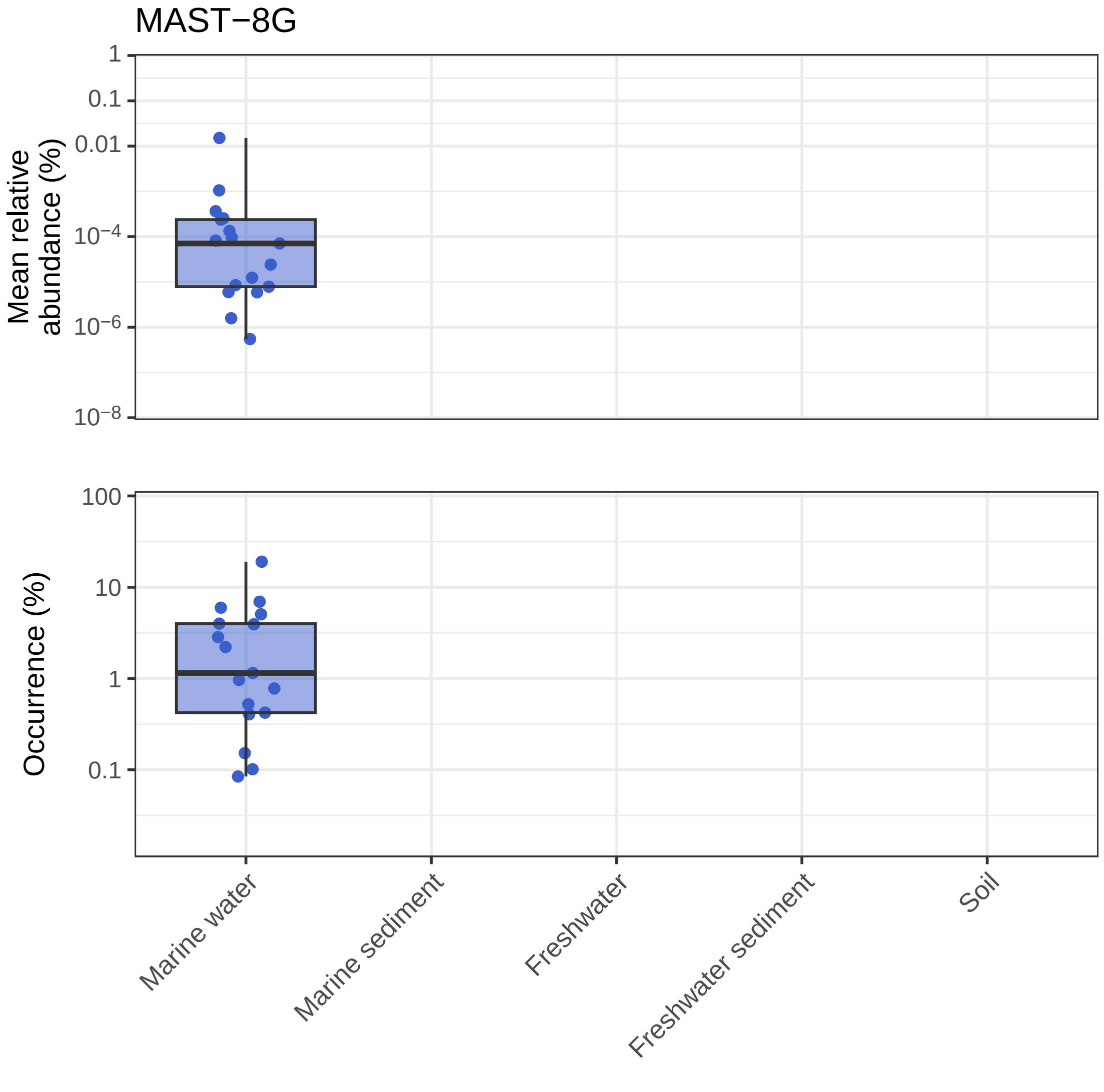
MAST−8G
Mean relative abundance (%)
1
0.1
0.01
10^{-4}
10^{-6}
10^{-8}
Occurrence (%)
100
10
1
0.1
Marine water
Marine sediment
Freshwater
Freshwater sediment
Soil

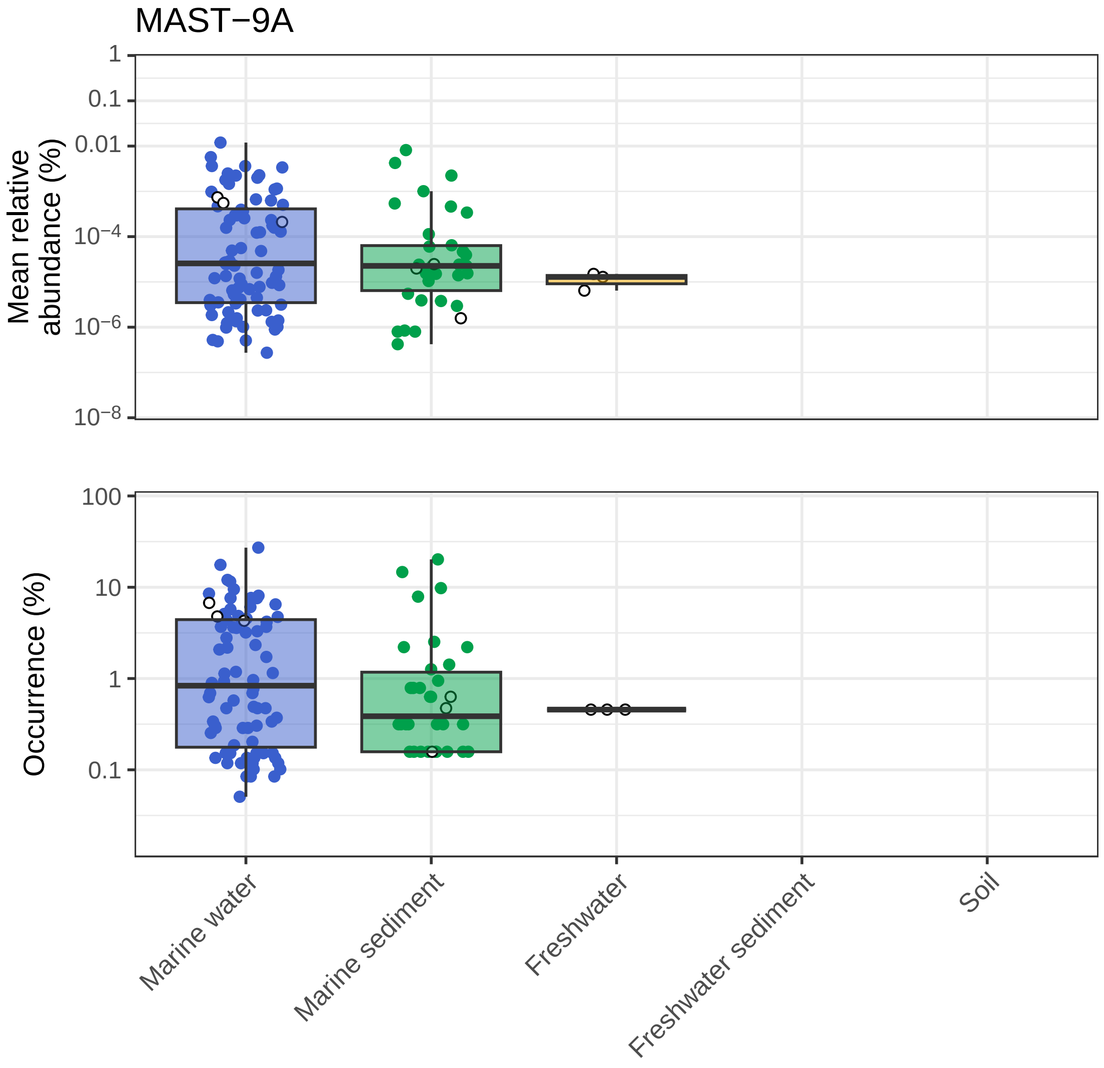

MAST−9A
Mean relative abundance (%)
1
0.1
0.01
10−4
10−6
10−8
Occurrence (%)
100
10
1
0.1
Marine water
Marine sediment
Freshwater
Freshwater sediment
Soil

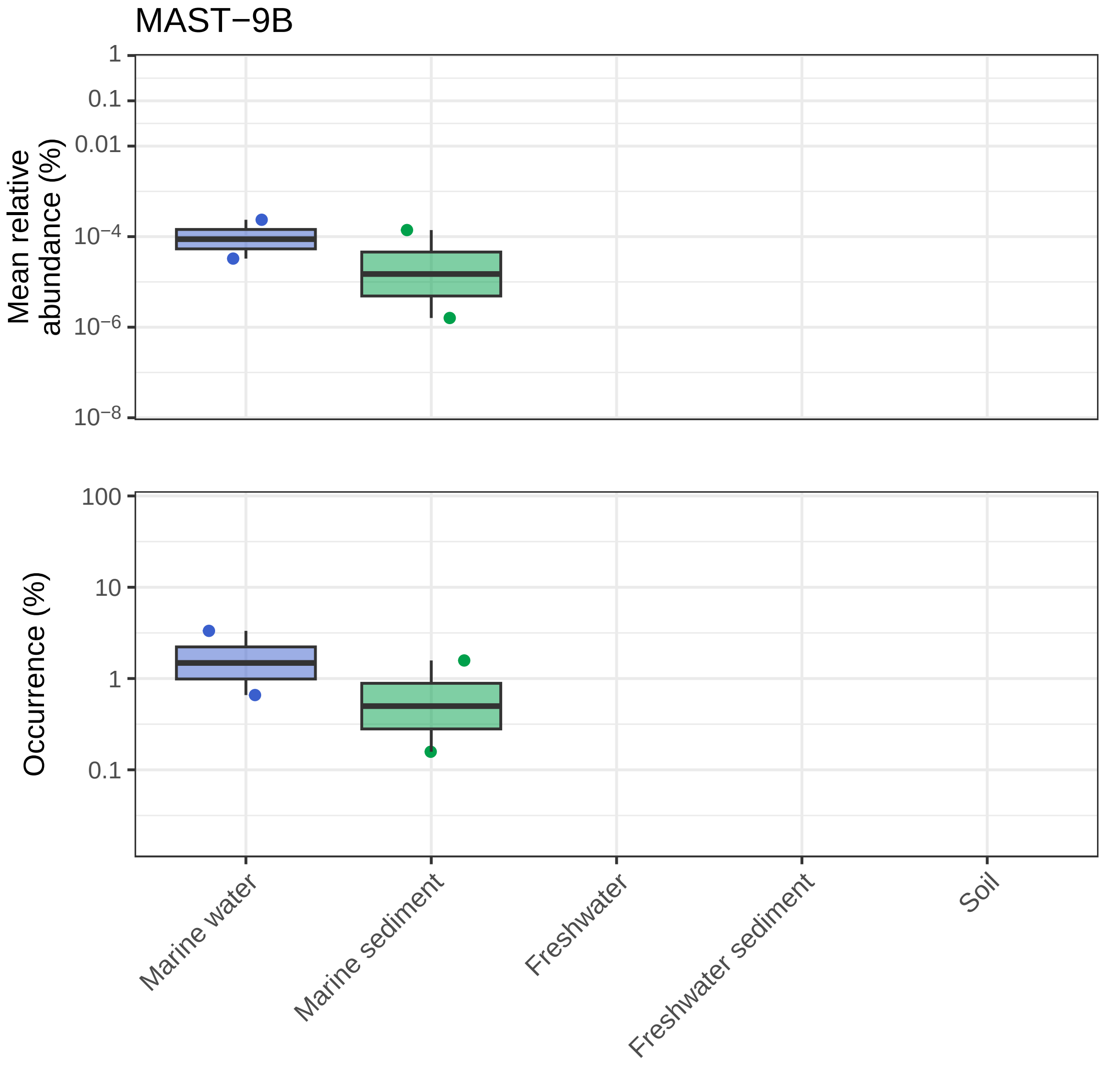
MAST−9B
Mean relative abundance (%)
1
0.1
0.01
10^{-4}
10^{-6}
10^{-8}
Occurrence (%)
100
10
1
0.1
Marine water
Marine sediment
Freshwater
Freshwater sediment
Soil

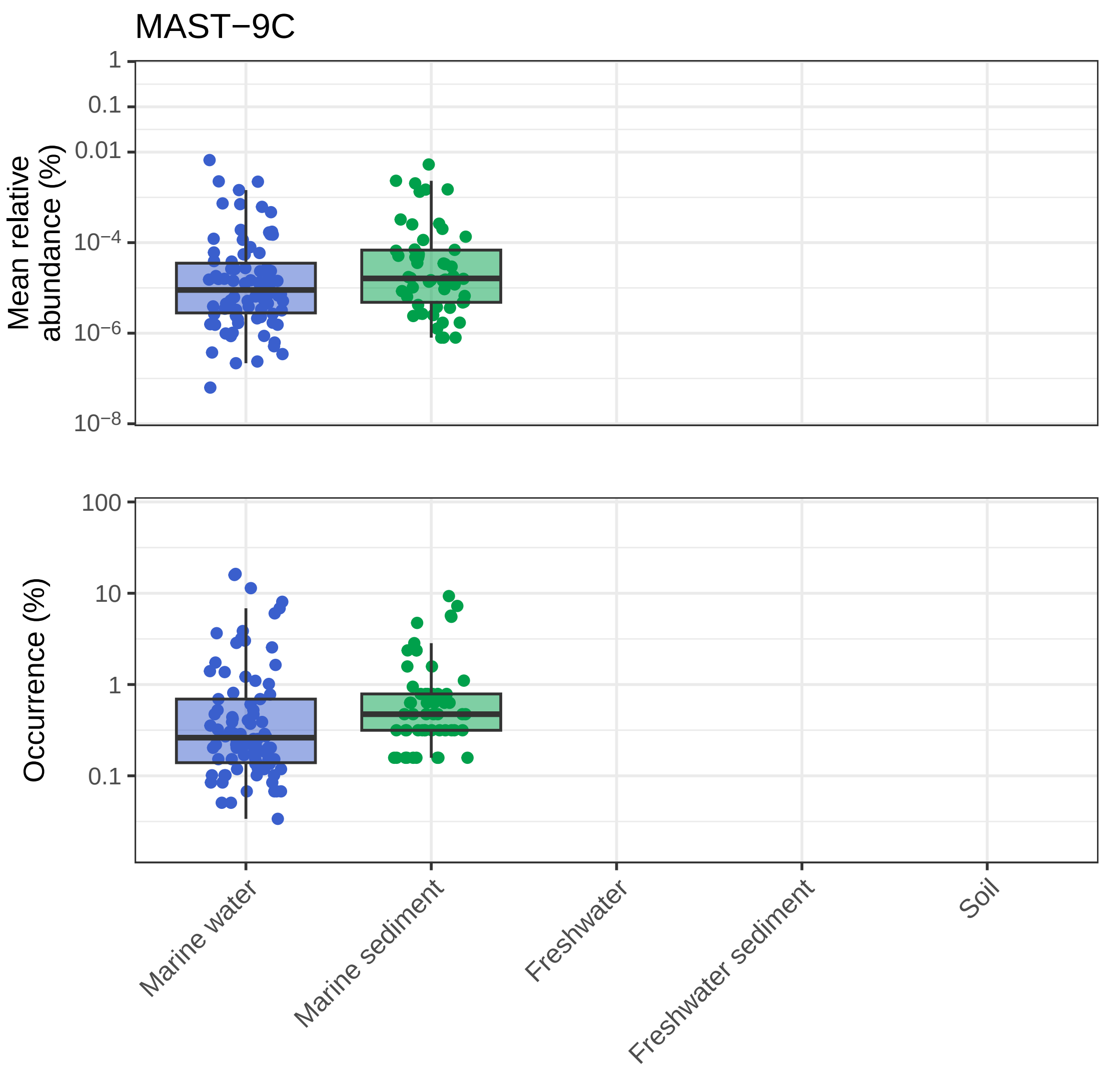

MAST−9C
Mean relative abundance (%)
1
0.1
0.01
10−4
10−6
10−8
Occurrence (%)
100
10
1
0.1
Marine water
Marine sediment
Freshwater
Freshwater sediment
Soil

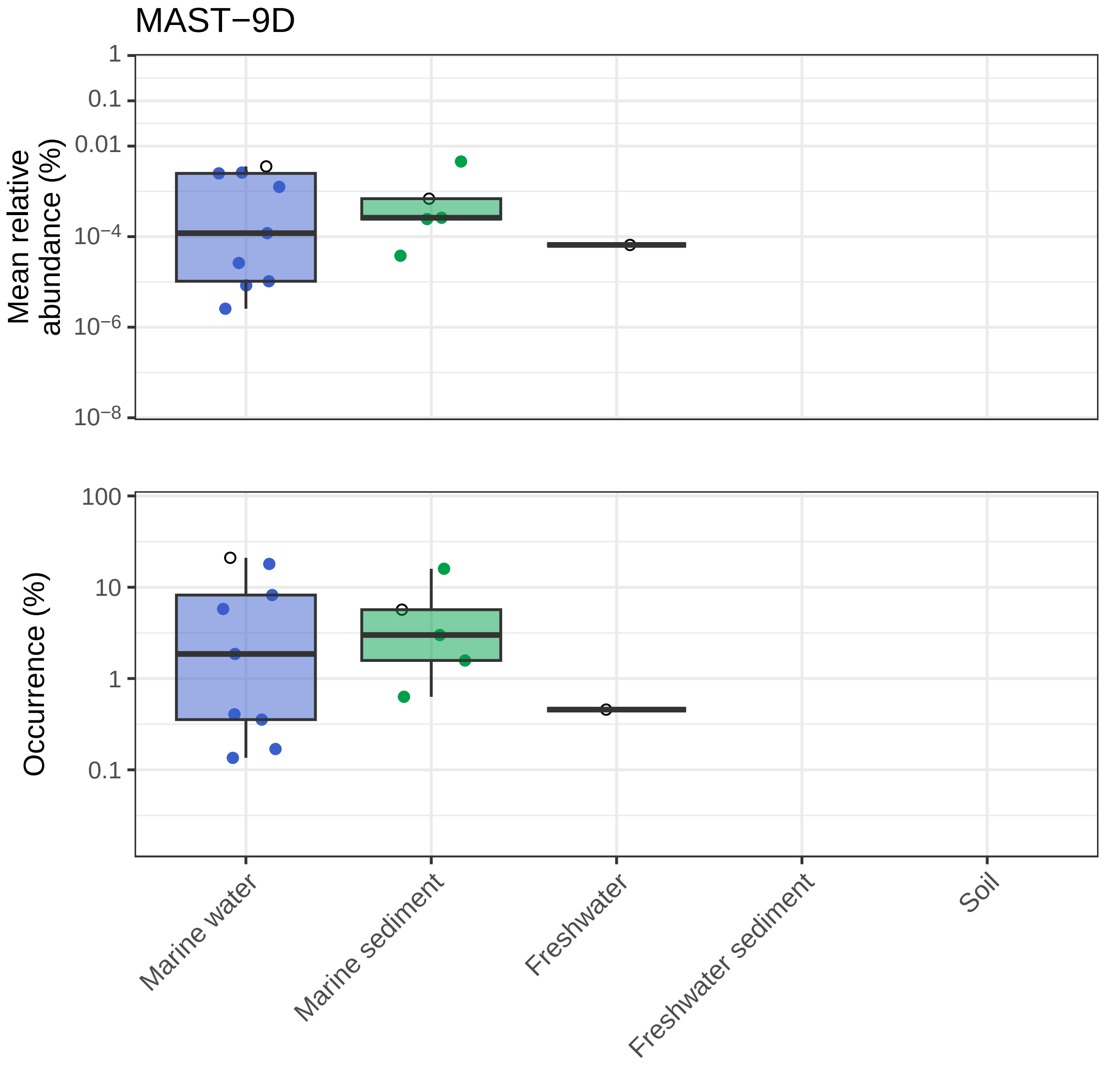
MAST−9D
Mean relative abundance (%)
1
0.1
0.01
10−4
10−6
10−8
Occurrence (%)
100
10
1
0.1
Marine water
Marine sediment
Freshwater
Freshwater sediment
Soil

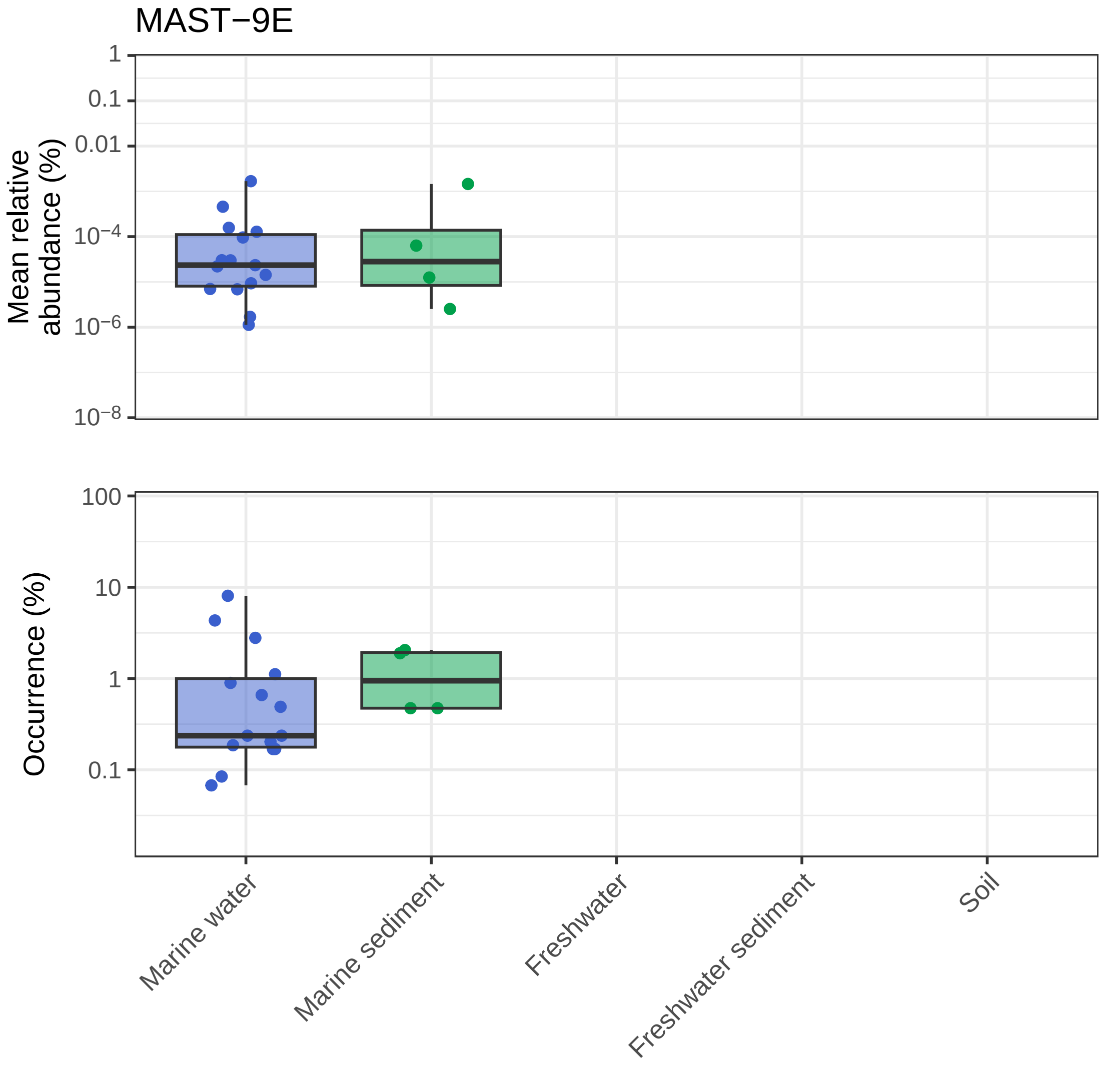

MAST−9E
Mean relative abundance (%)
1
0.1
0.01
10^{-4}
10^{-6}
10^{-8}
Occurrence (%)
100
10
1
0.1
Marine water
Marine sediment
Freshwater
Freshwater sediment
Soil

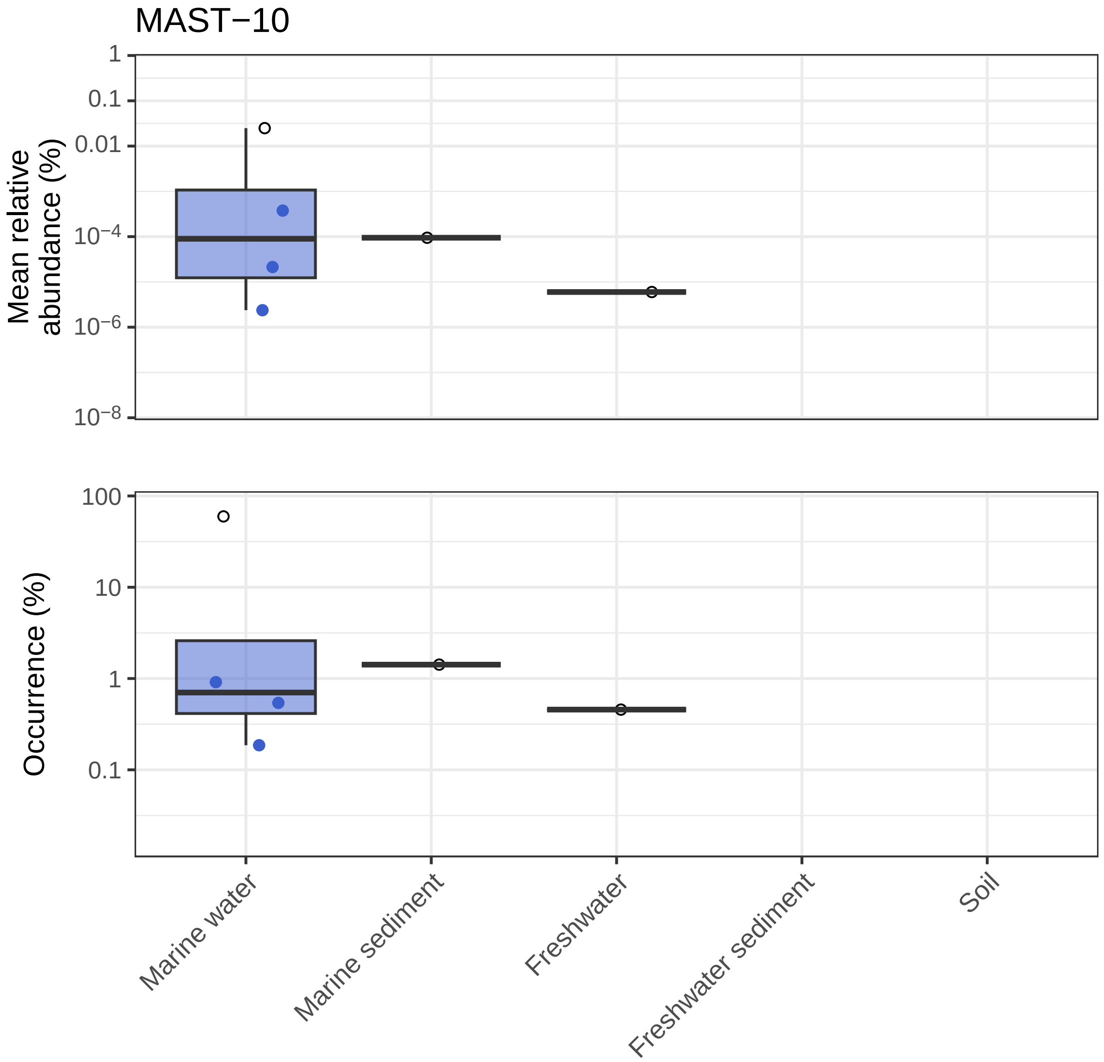
MAST−10
Mean relative abundance (%)
1
0.1
0.01
10^{-4}
10^{-6}
10^{-8}
Occurrence (%)
100
10
1
0.1
Marine water
Marine sediment
Freshwater
Freshwater sediment
Soil

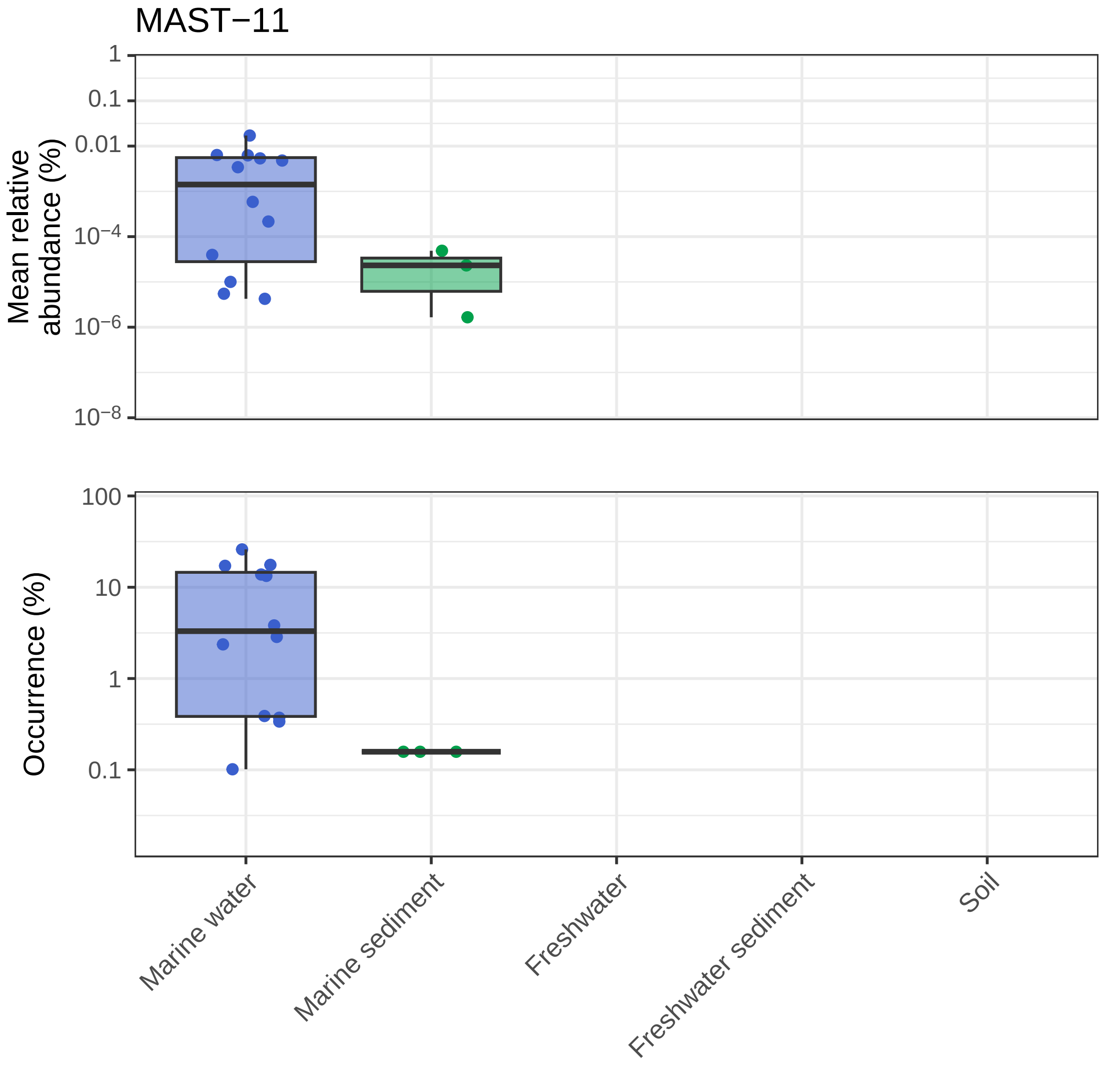
MAST−11
Mean relative abundance (%)
1
0.1
0.01
10^{-4}
10^{-6}
10^{-8}
Occurrence (%)
100
10
1
0.1
Marine water
Marine sediment
Freshwater
Freshwater sediment
Soil

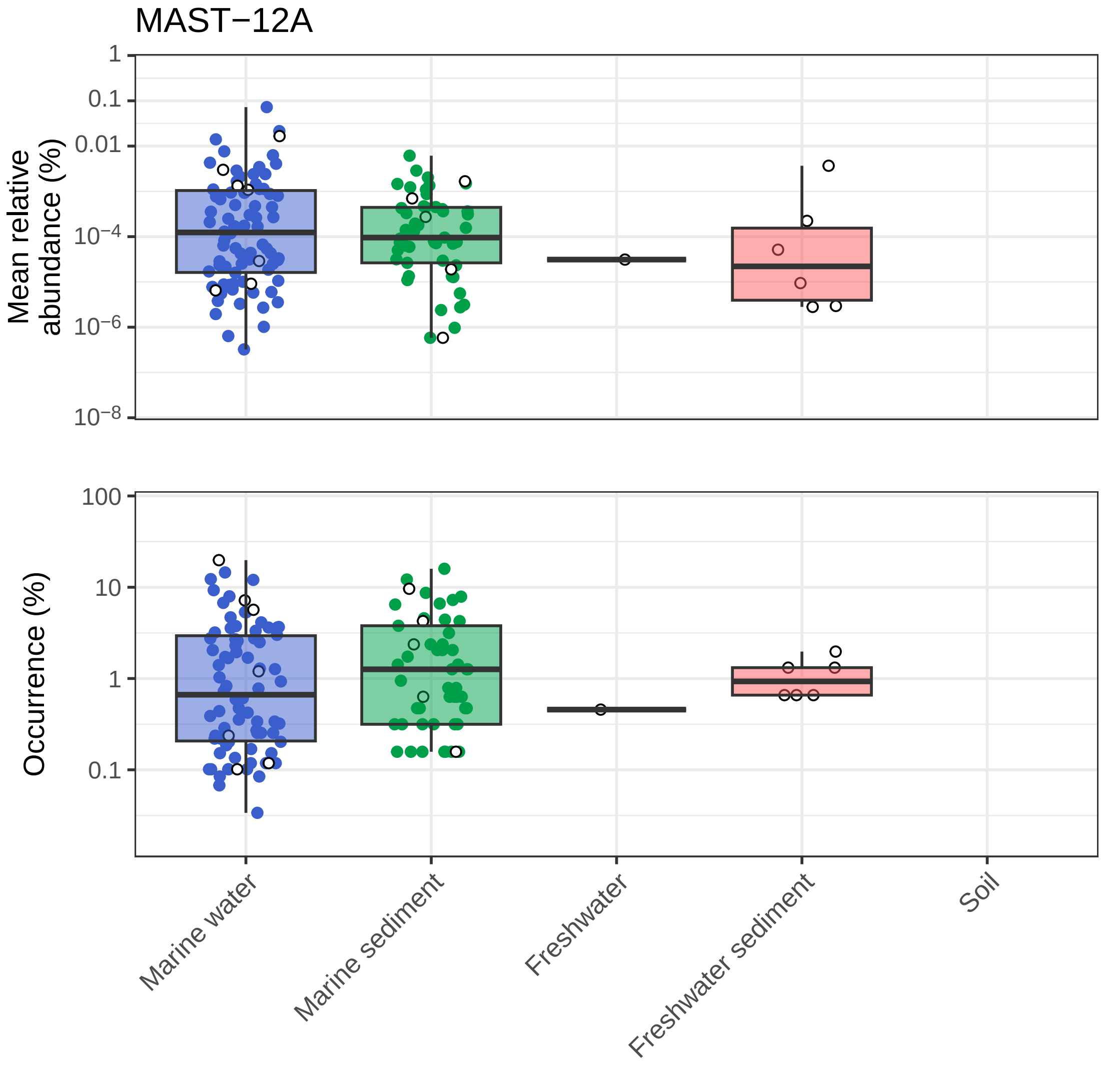
MAST−12A
Mean relative abundance (%)
1
0.1
0.01
10^{-4}
10^{-6}
10^{-8}
Occurrence (%)
100
10
1
0.1
Marine water
Marine sediment
Freshwater
Freshwater sediment
Soil

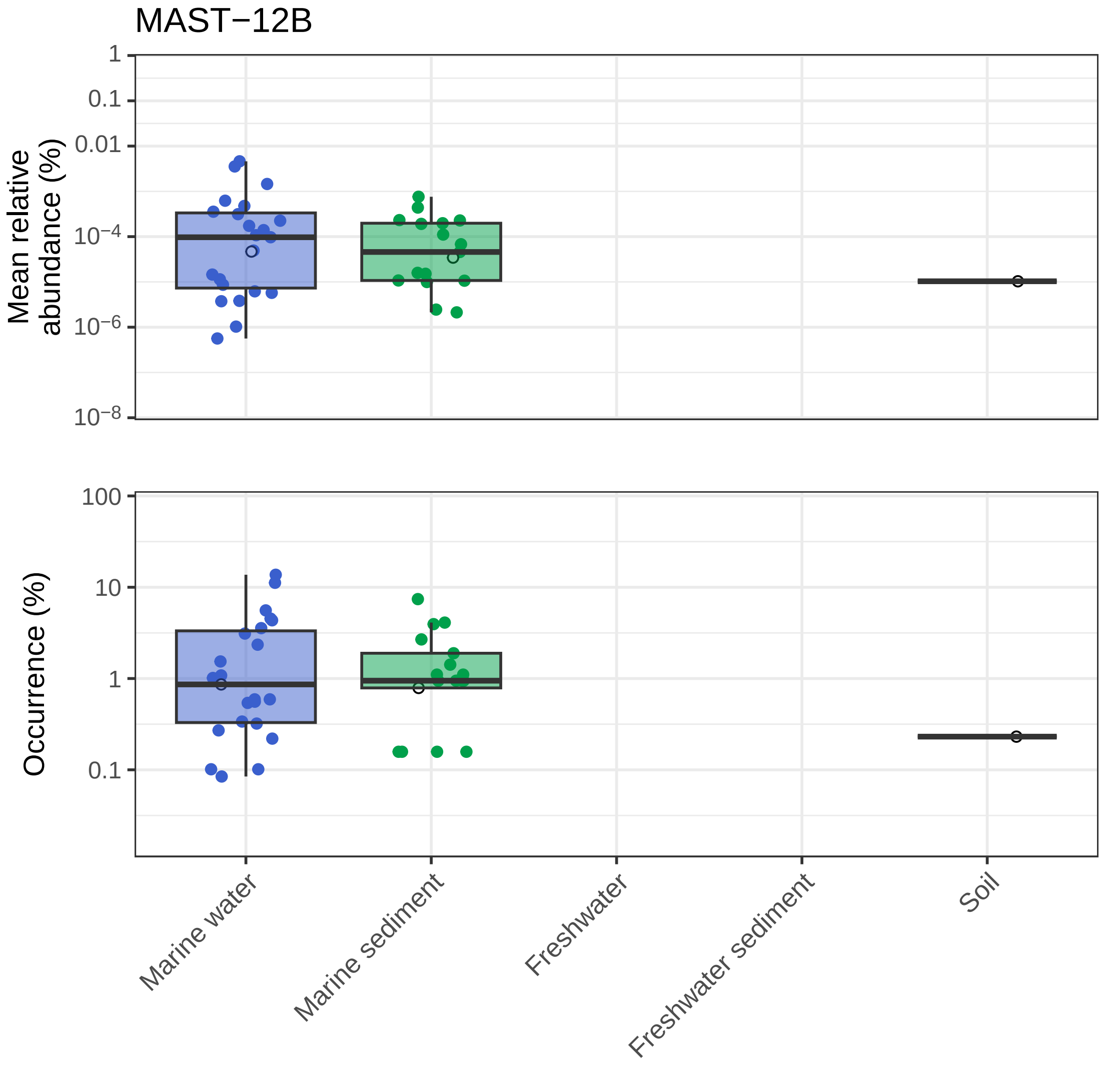

MAST−12B
Mean relative abundance (%)
1
0.1
0.01
10^{-4}
10^{-6}
10^{-8}
Occurrence (%)
100
10
1
0.1
Marine water
Marine sediment
Freshwater
Freshwater sediment
Soil

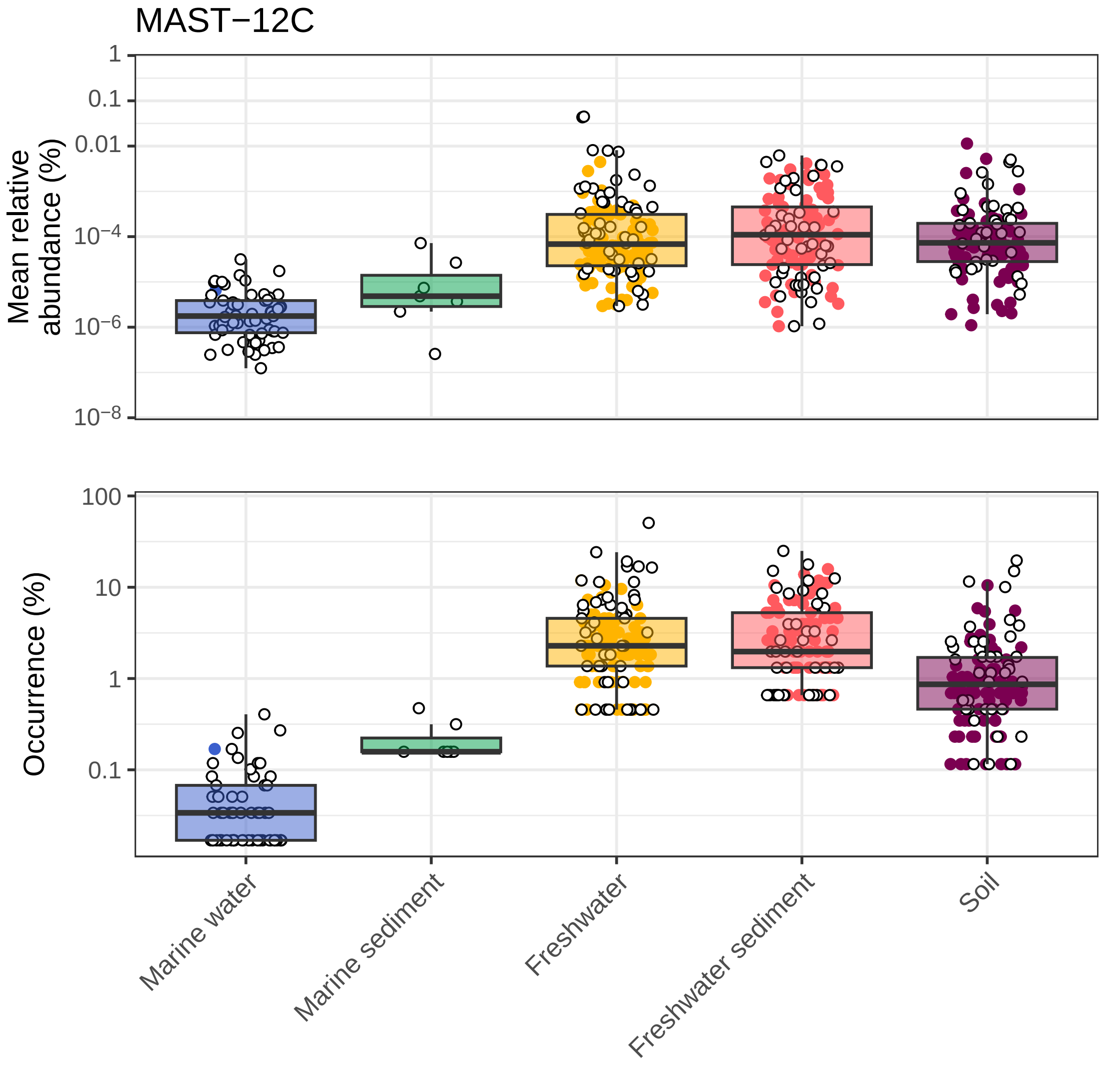
MAST−12C
Mean relative abundance (%)
1
0.1
0.01
10⁻⁴
10⁻⁶
10⁻⁸
Occurrence (%)
100
10
1
0.1
Marine water
Marine sediment
Freshwater
Freshwater sediment
Soil

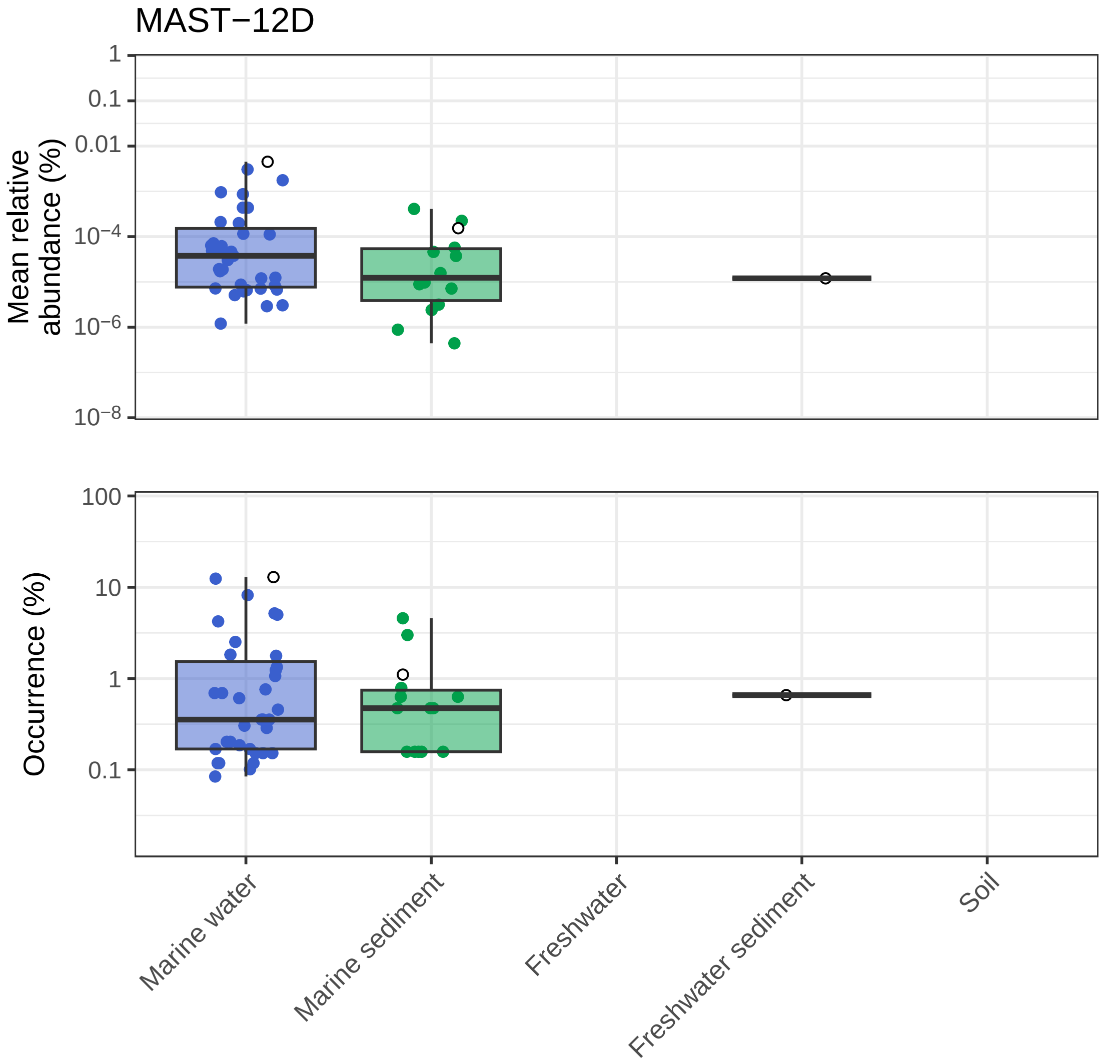

MAST−12D
Mean relative abundance (%)
1
0.1
0.01
10−4
10−6
10−8
Occurrence (%)
100
10
1
0.1
Marine water
Marine sediment
Freshwater
Freshwater sediment
Soil

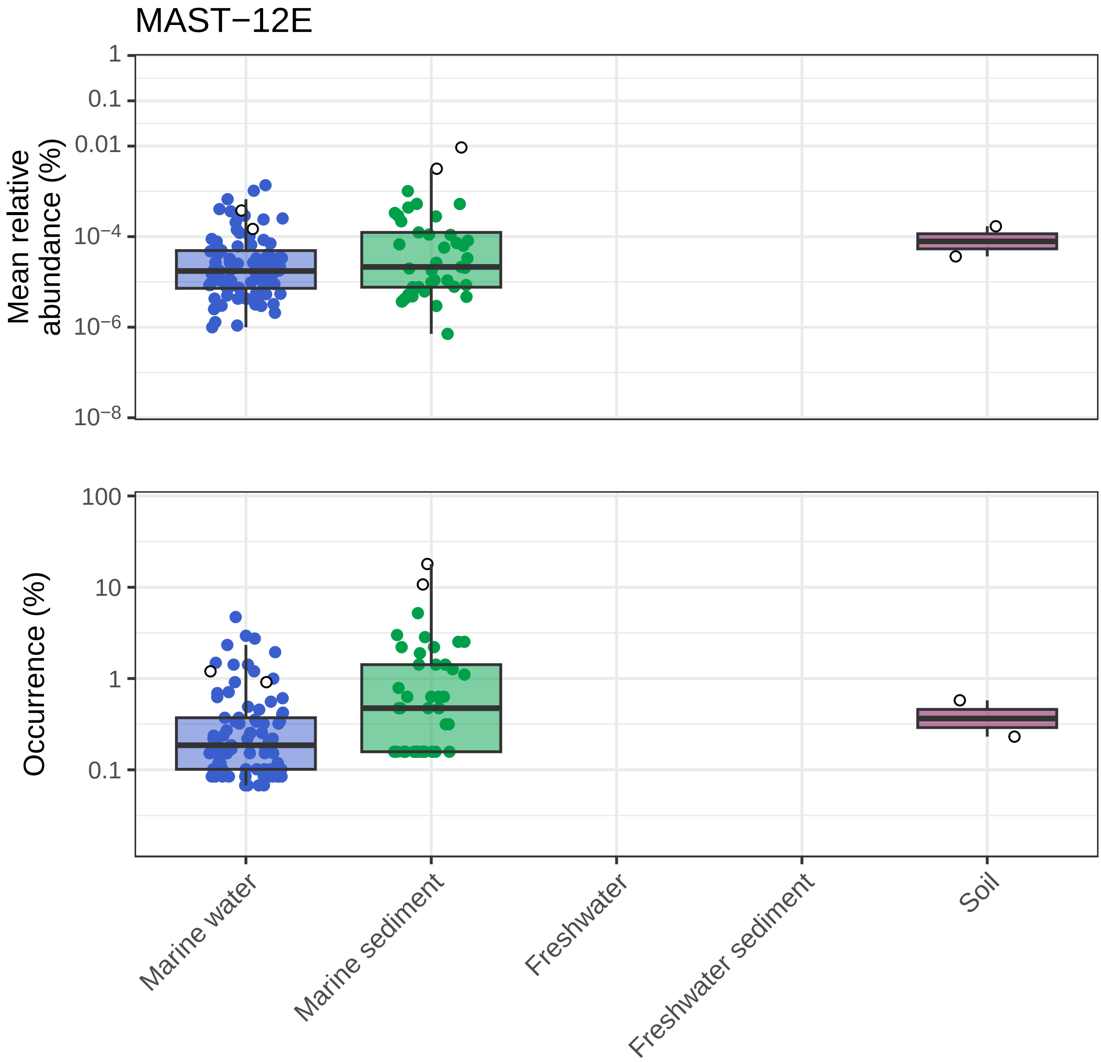

MAST−12E
Mean relative abundance (%)
1
0.1
0.01
10^{-4}
10^{-6}
10^{-8}
Occurrence (%)
100
10
1
0.1
Marine water
Marine sediment
Freshwater
Freshwater sediment
Soil

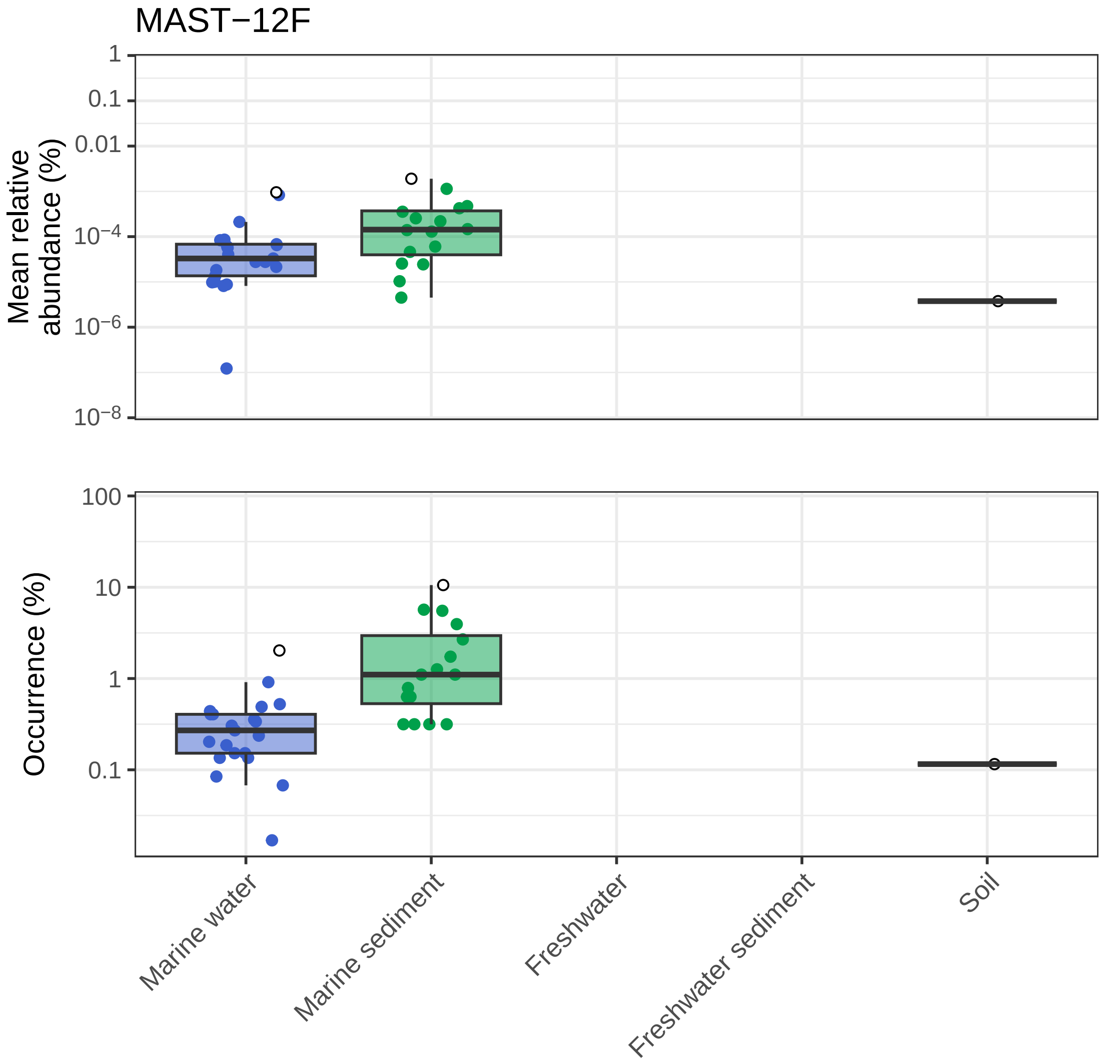

MAST−12F
Mean relative abundance (%)
1
0.1
0.01
10−4
10−6
10−8
Occurrence (%)
100
10
1
0.1
Marine water
Marine sediment
Freshwater
Freshwater sediment
Soil

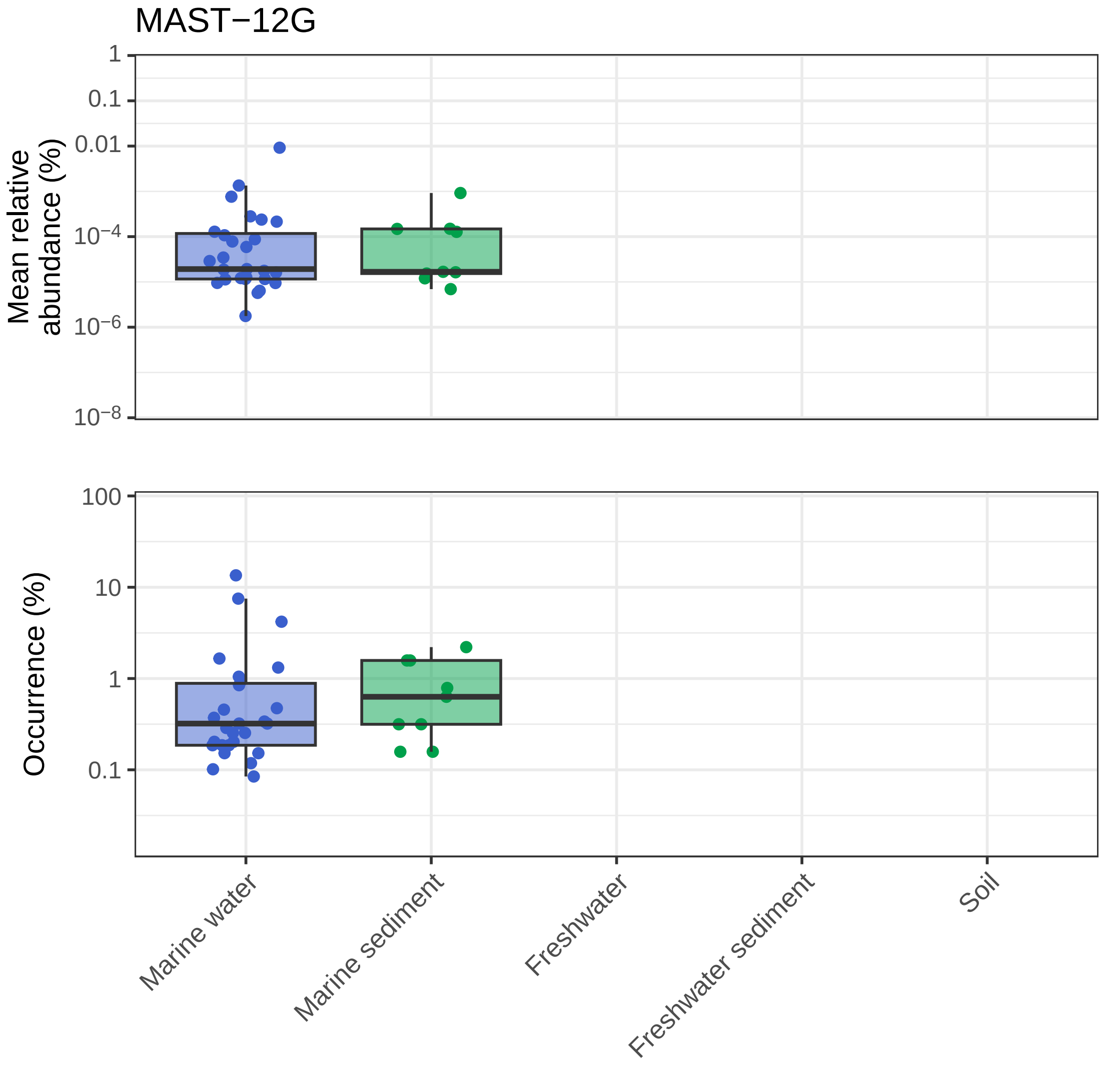
MAST−12G
Mean relative abundance (%)
1
0.1
0.01
10^{-4}
10^{-6}
10^{-8}
Occurrence (%)
100
10
1
0.1
Marine water
Marine sediment
Freshwater
Freshwater sediment
Soil

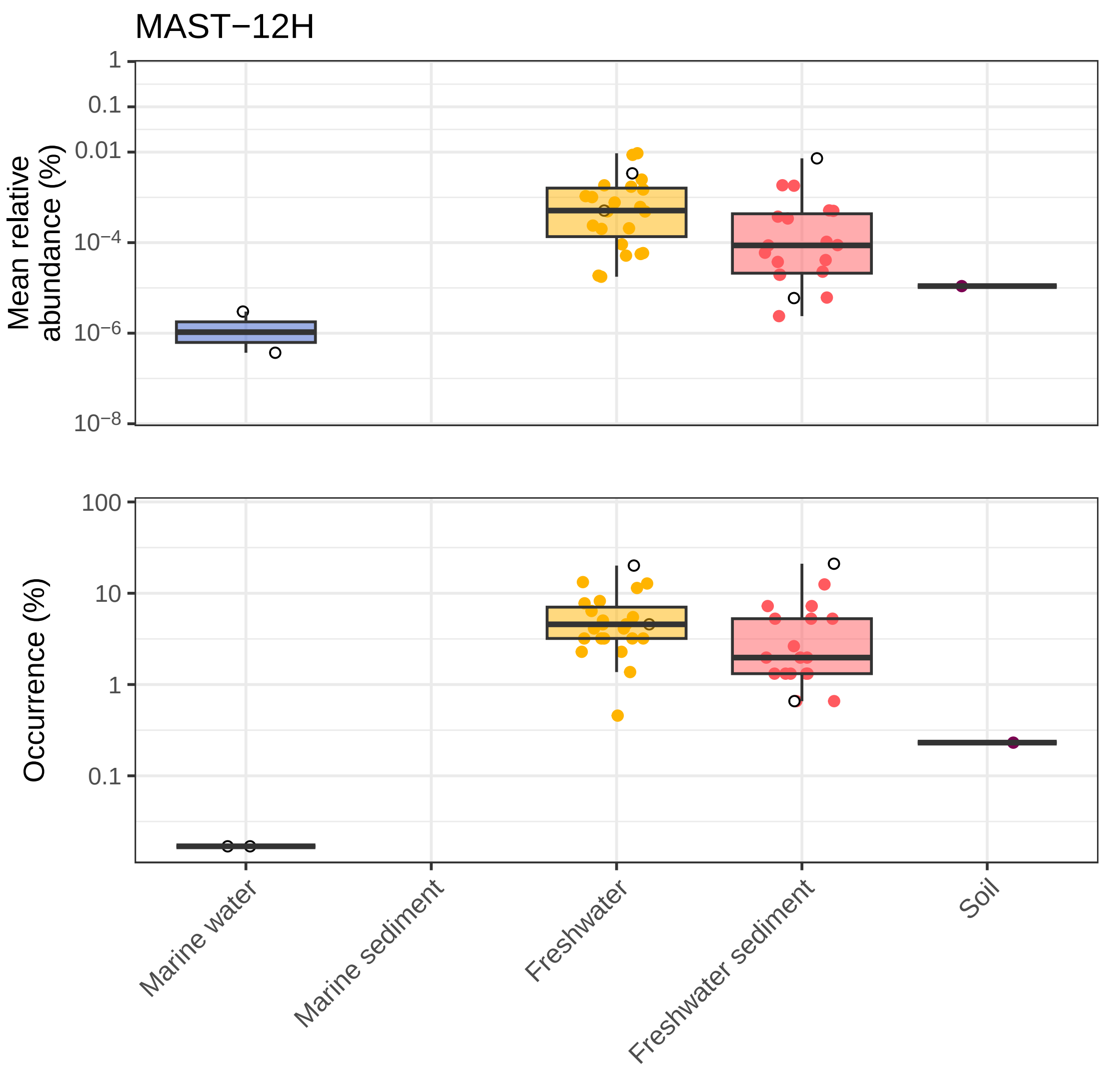
MAST−12H
Mean relative abundance (%)
1
0.1
0.01
10^{-4}
10^{-6}
10^{-8}
Occurrence (%)
100
10
1
0.1
Marine water
Marine sediment
Freshwater
Freshwater sediment
Soil

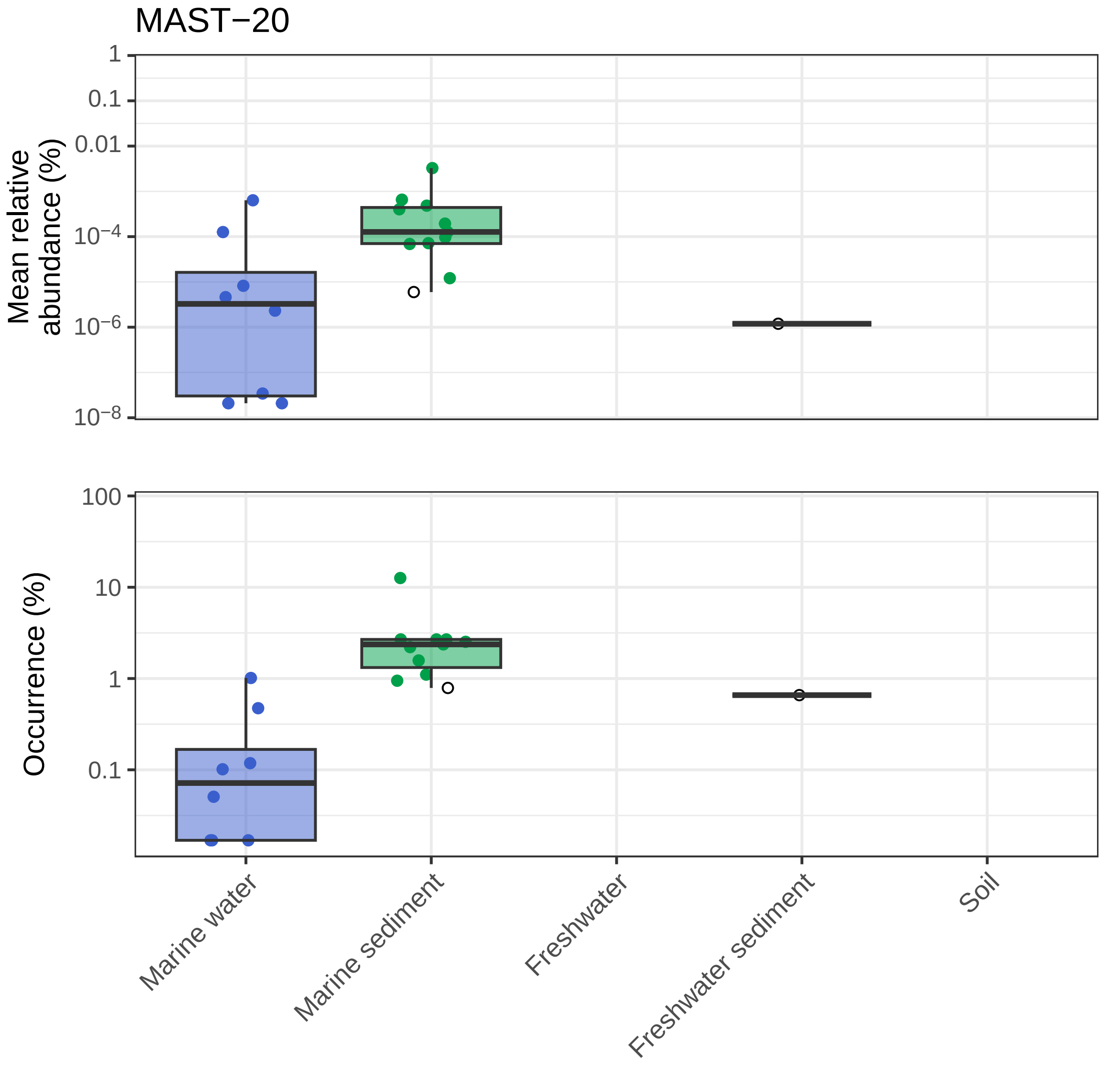
MAST−20
Mean relative abundance (%)
1
0.1
0.01
10−4
10−6
10−8
Occurrence (%)
100
10
1
0.1
Marine water
Marine sediment
Freshwater
Freshwater sediment
Soil

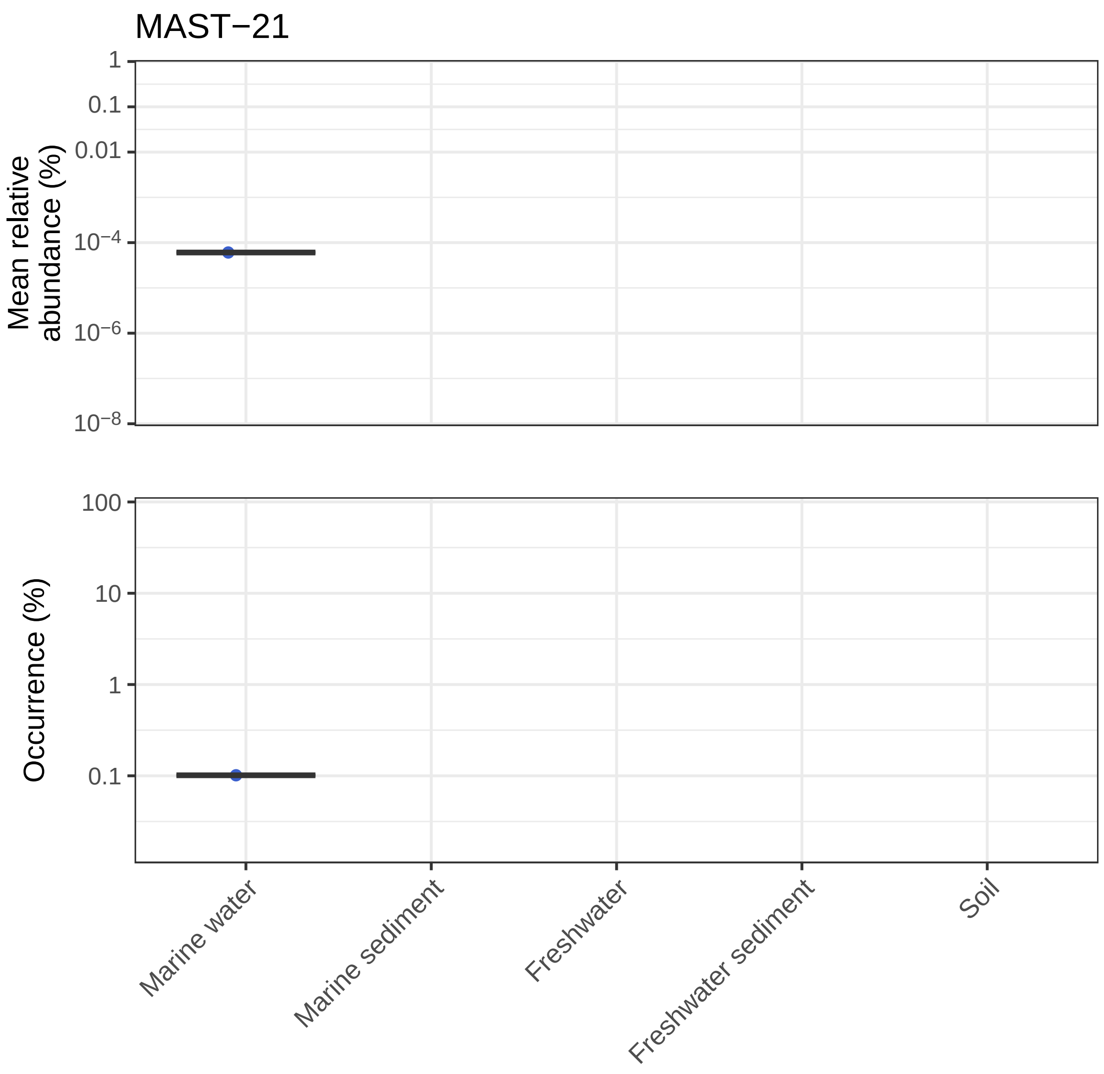
MAST−21
Mean relative abundance (%)
1
0.1
0.01
10−4
10−6
10−8
Occurrence (%)
100
10
1
0.1
Marine water
Marine sediment
Freshwater
Freshwater sediment
Soil

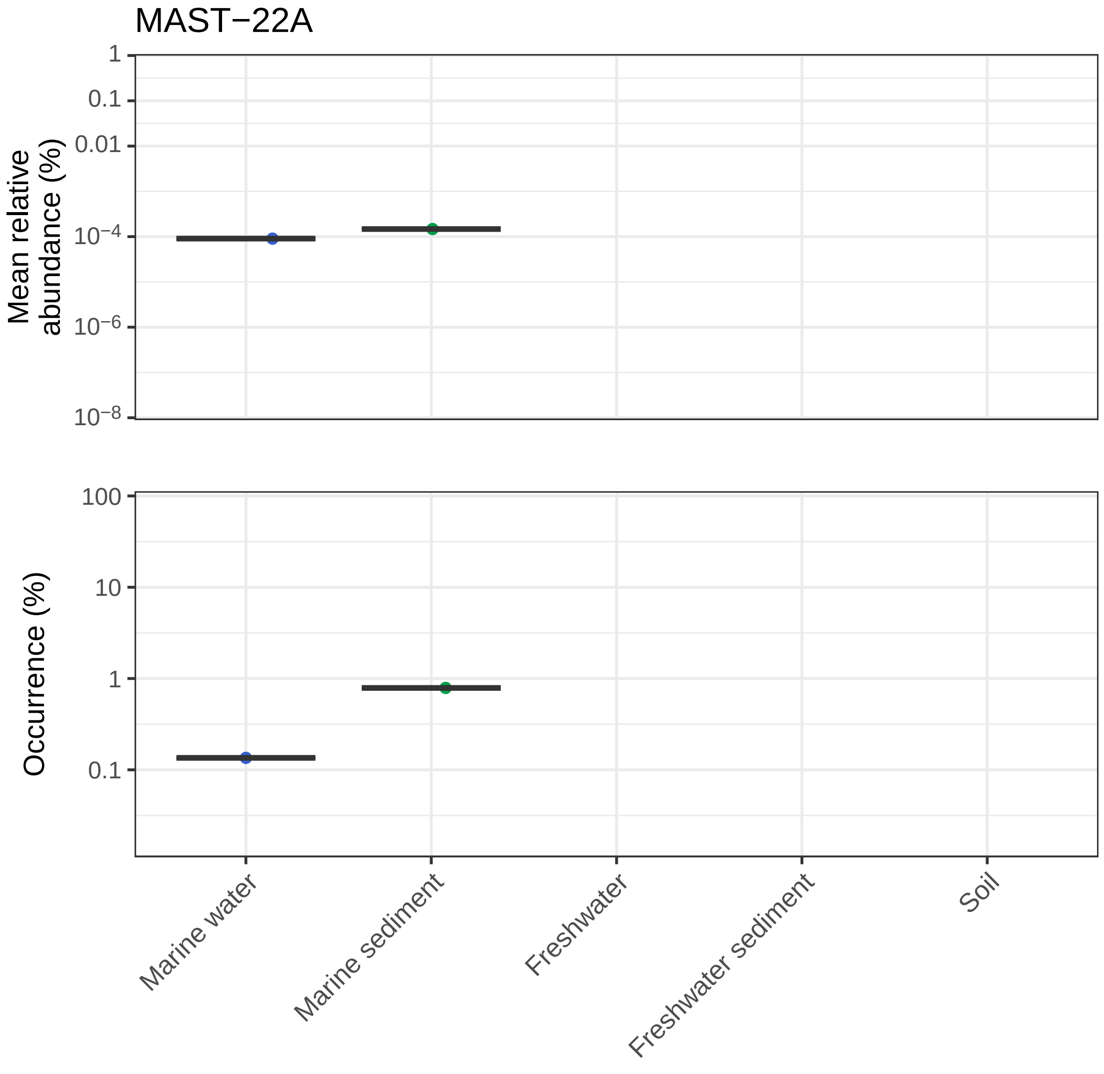
MAST−22A
Mean relative abundance (%)
1
0.1
0.01
10^{-4}
10^{-6}
10^{-8}
Occurrence (%)
100
10
1
0.1
Marine water
Marine sediment
Freshwater
Freshwater sediment
Soil

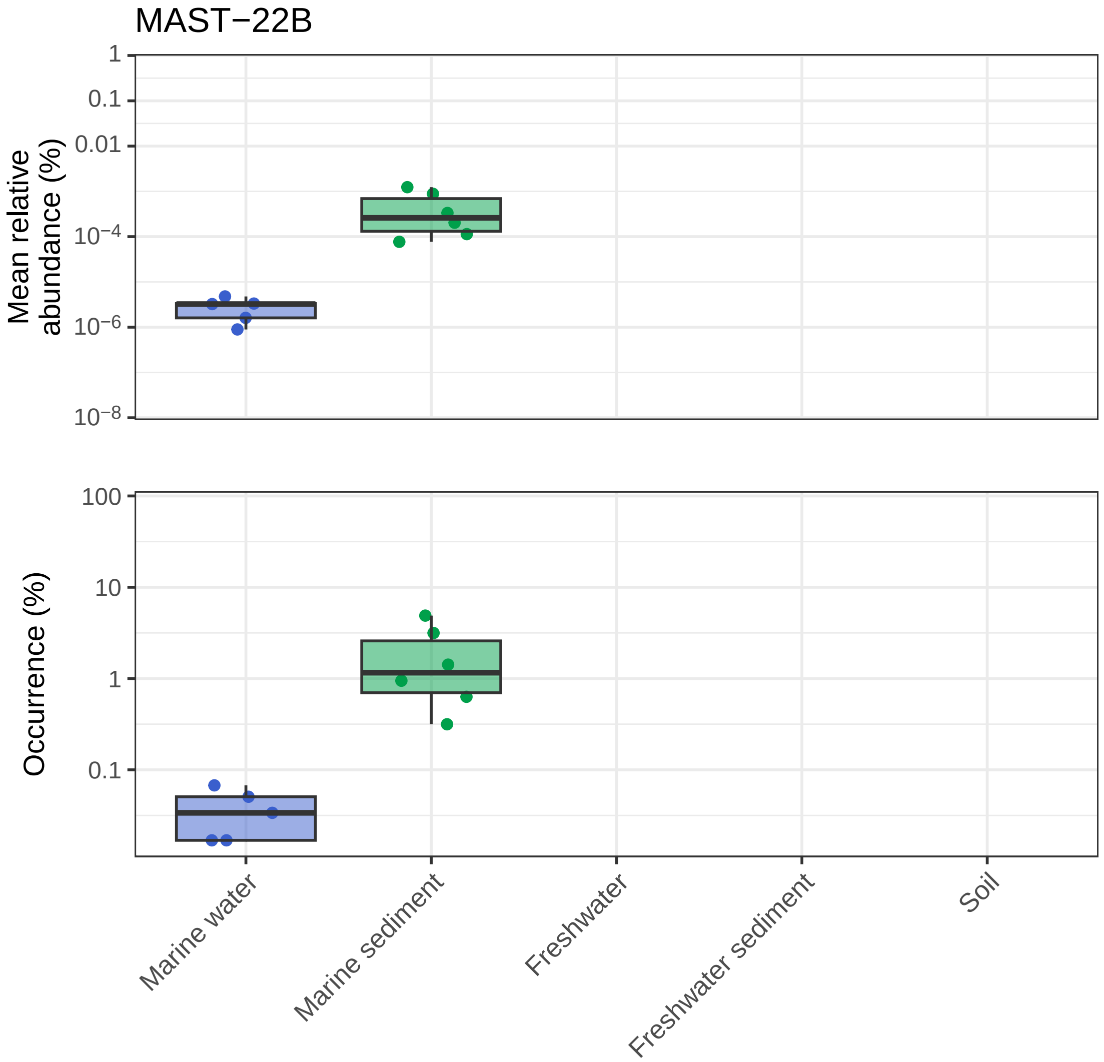
MAST−22B
Mean relative abundance (%)
1
0.1
0.01
10^{-4}
10^{-6}
10^{-8}
Occurrence (%)
100
10
1
0.1
Marine water
Marine sediment
Freshwater
Freshwater sediment
Soil

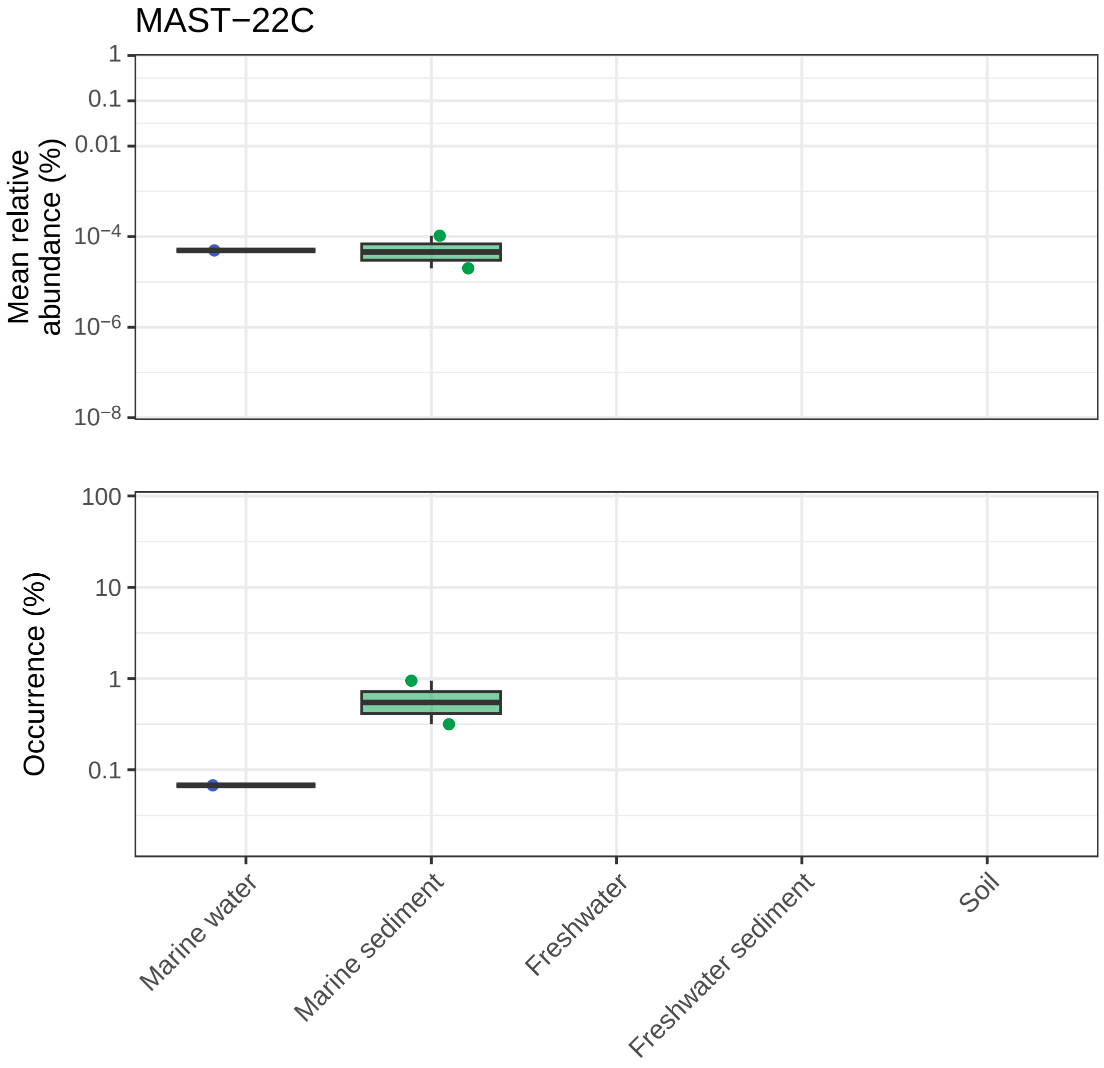
MAST−22C
Mean relative abundance (%)
1
0.1
0.01
10^{-4}
10^{-6}
10^{-8}
Occurrence (%)
100
10
1
0.1
Marine water
Marine sediment
Freshwater
Freshwater sediment
Soil

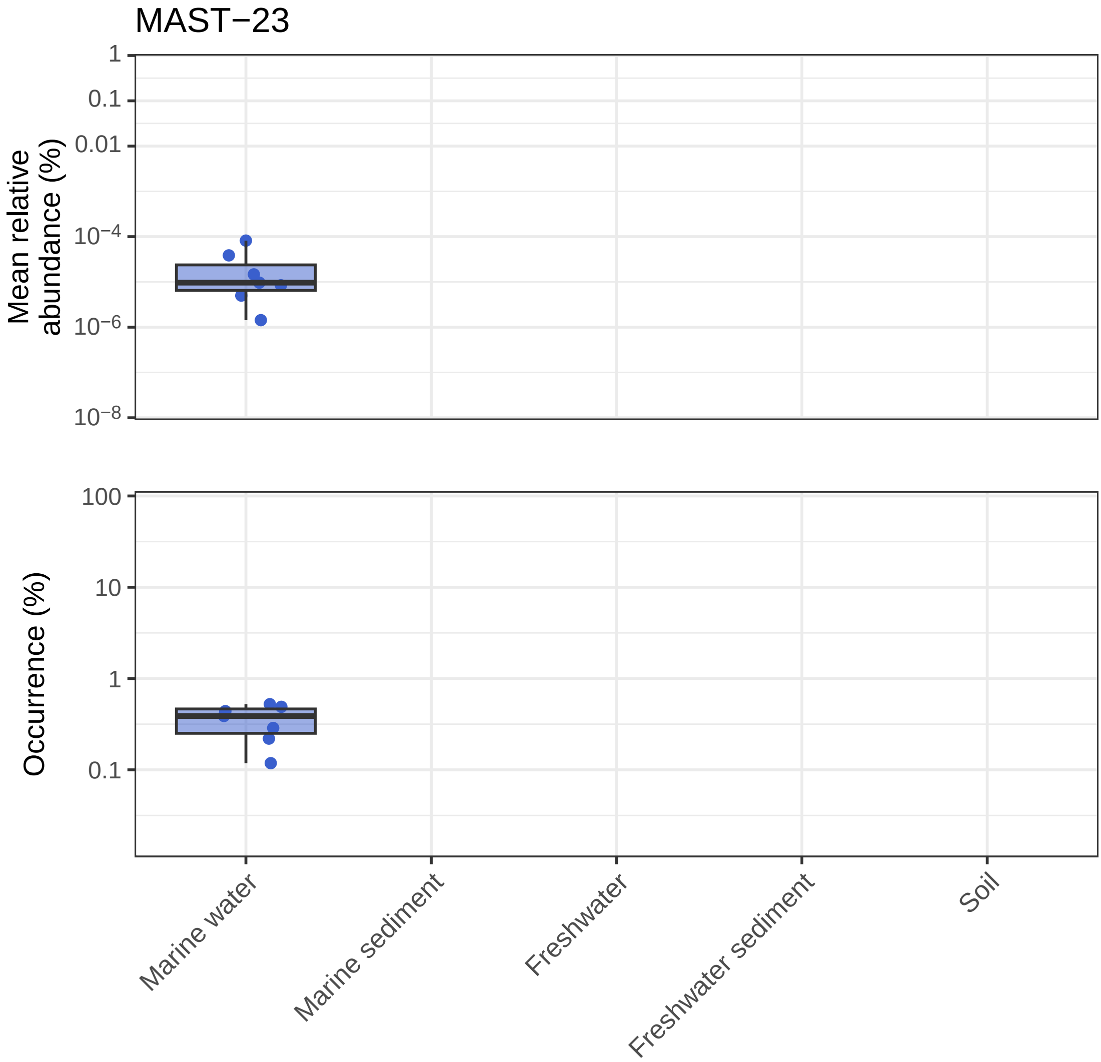

MAST−23
Mean relative abundance (%)
1
0.1
0.01
10−4
10−6
10−8
Occurrence (%)
100
10
1
0.1
Marine water
Marine sediment
Freshwater
Freshwater sediment
Soil

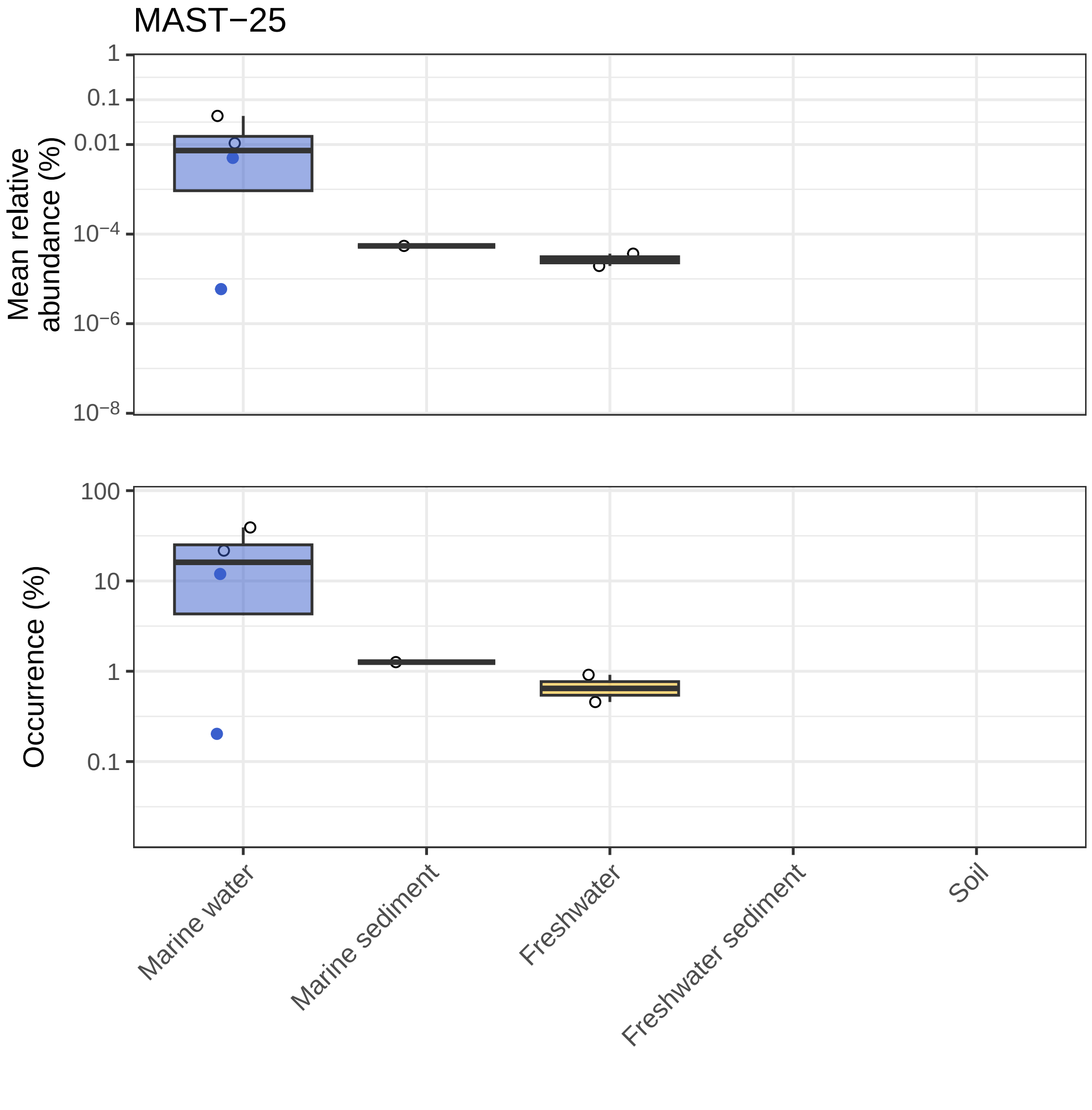

MAST−25
Mean relative abundance (%)
1
0.1
0.01
10^{-4}
10^{-6}
10^{-8}
Occurrence (%)
100
10
1
0.1
Marine water
Marine sediment
Freshwater
Freshwater sediment
Soil

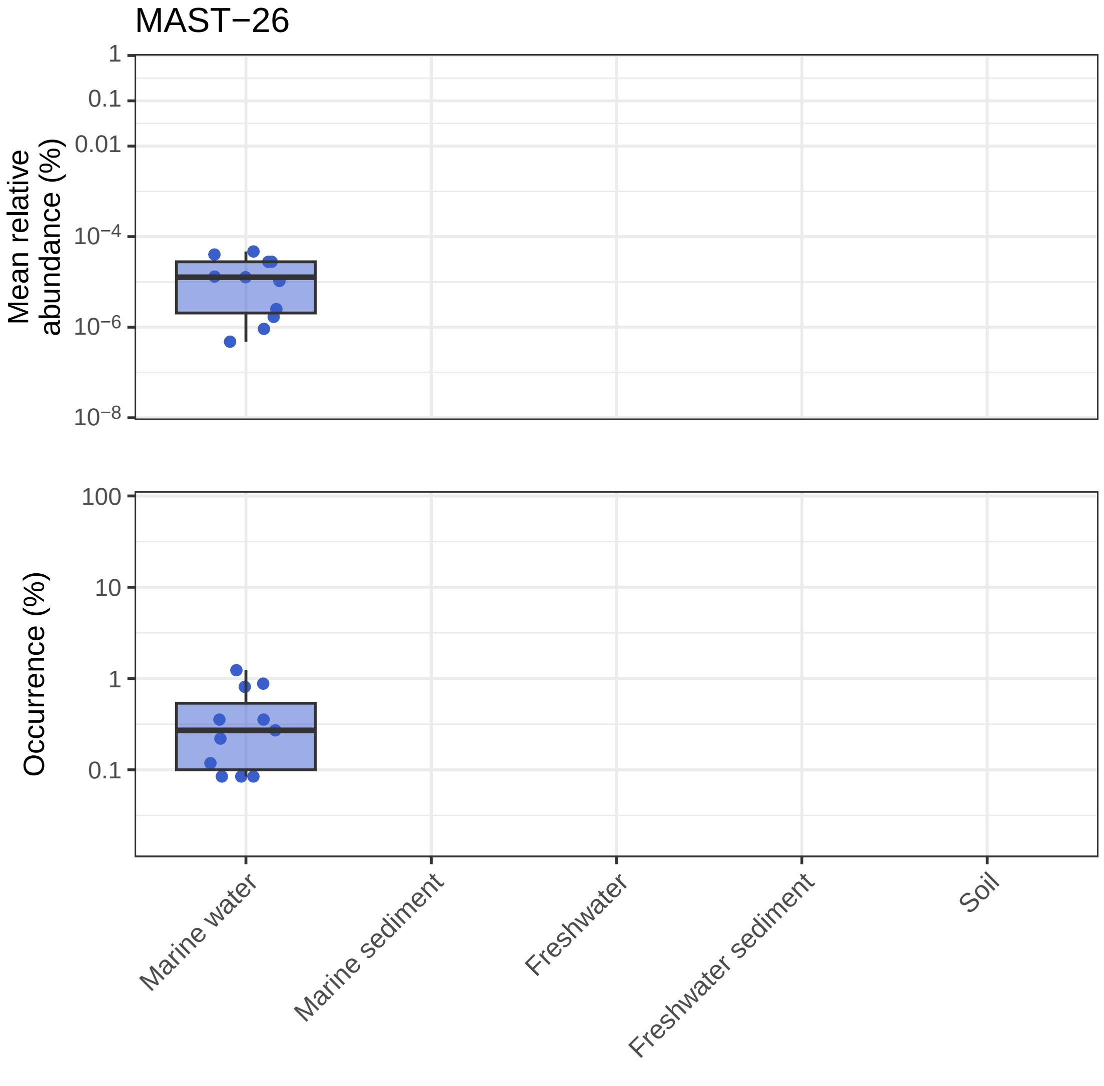

MAST−26
Mean relative abundance (%)
1
0.1
0.01
10^{-4}
10^{-6}
10^{-8}
Occurrence (%)
100
10
1
0.1
Marine water
Marine sediment
Freshwater
Freshwater sediment
Soil

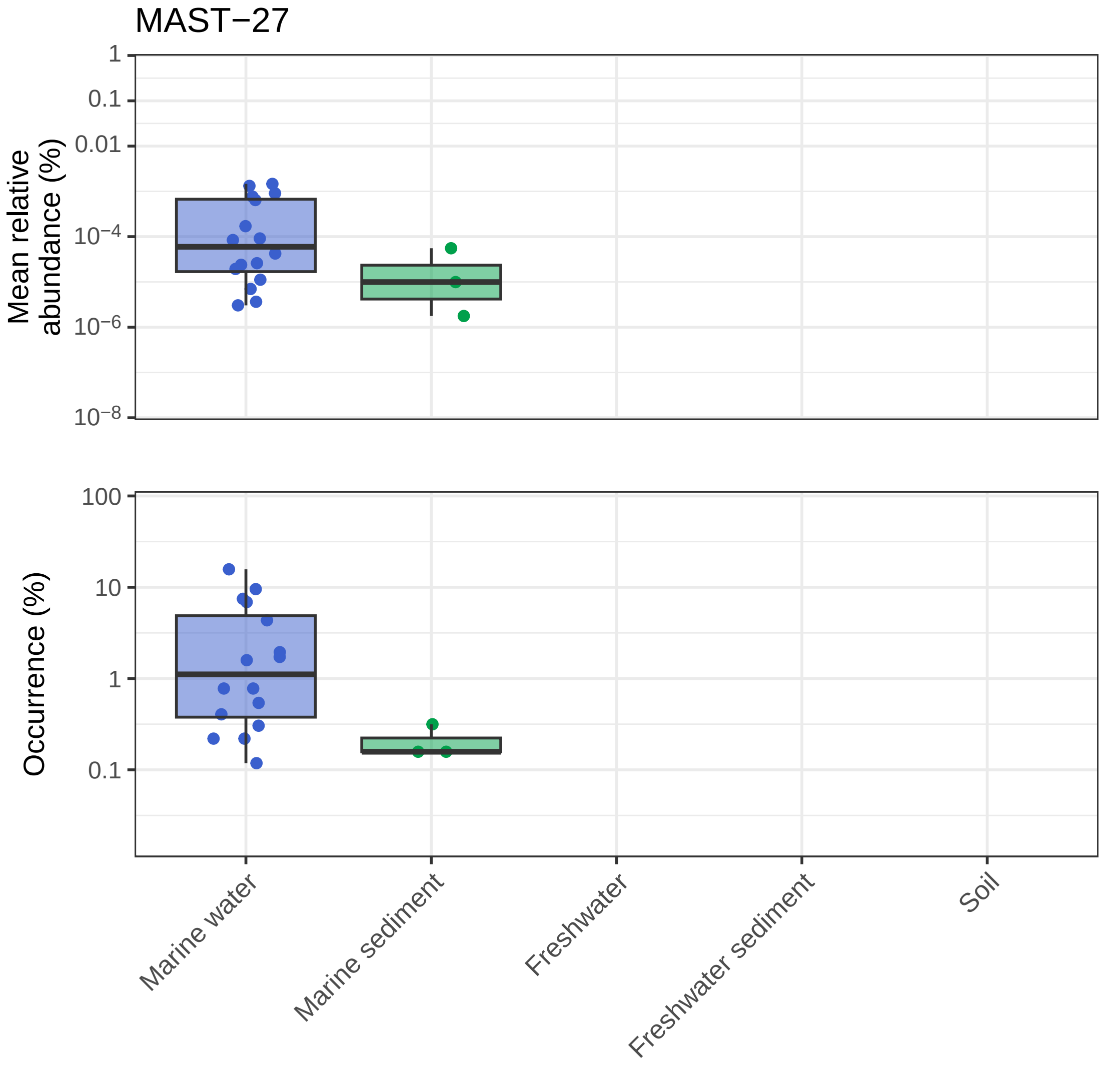
MAST−27
Mean relative abundance (%)
1
0.1
0.01
10−4
10−6
10−8
Occurrence (%)
100
10
1
0.1
Marine water
Marine sediment
Freshwater
Freshwater sediment
Soil

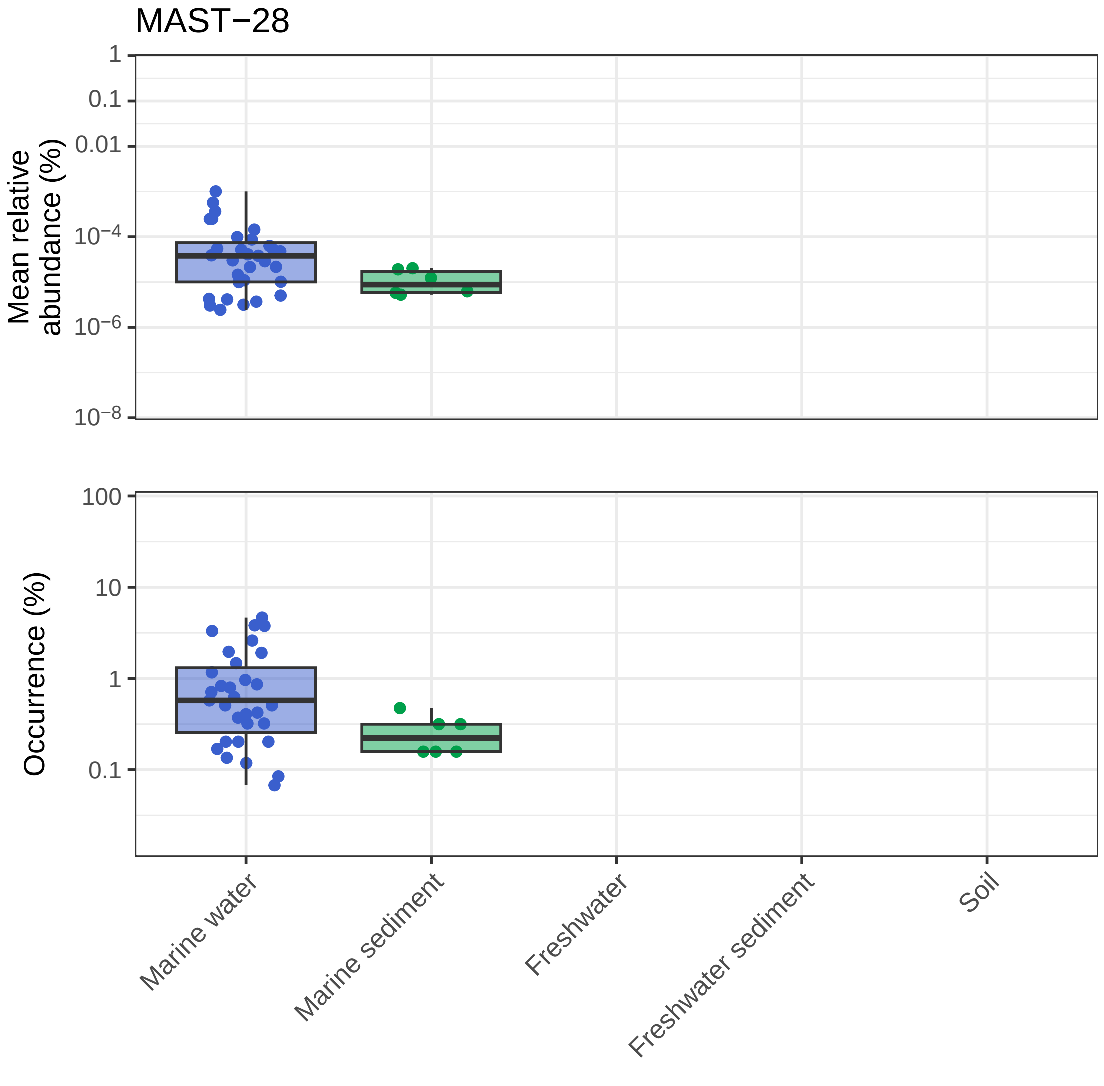

MAST−28
Mean relative abundance (%)
1
0.1
0.01
10^{-4}
10^{-6}
10^{-8}
Occurrence (%)
100
10
1
0.1
Marine water
Marine sediment
Freshwater
Freshwater sediment
Soil

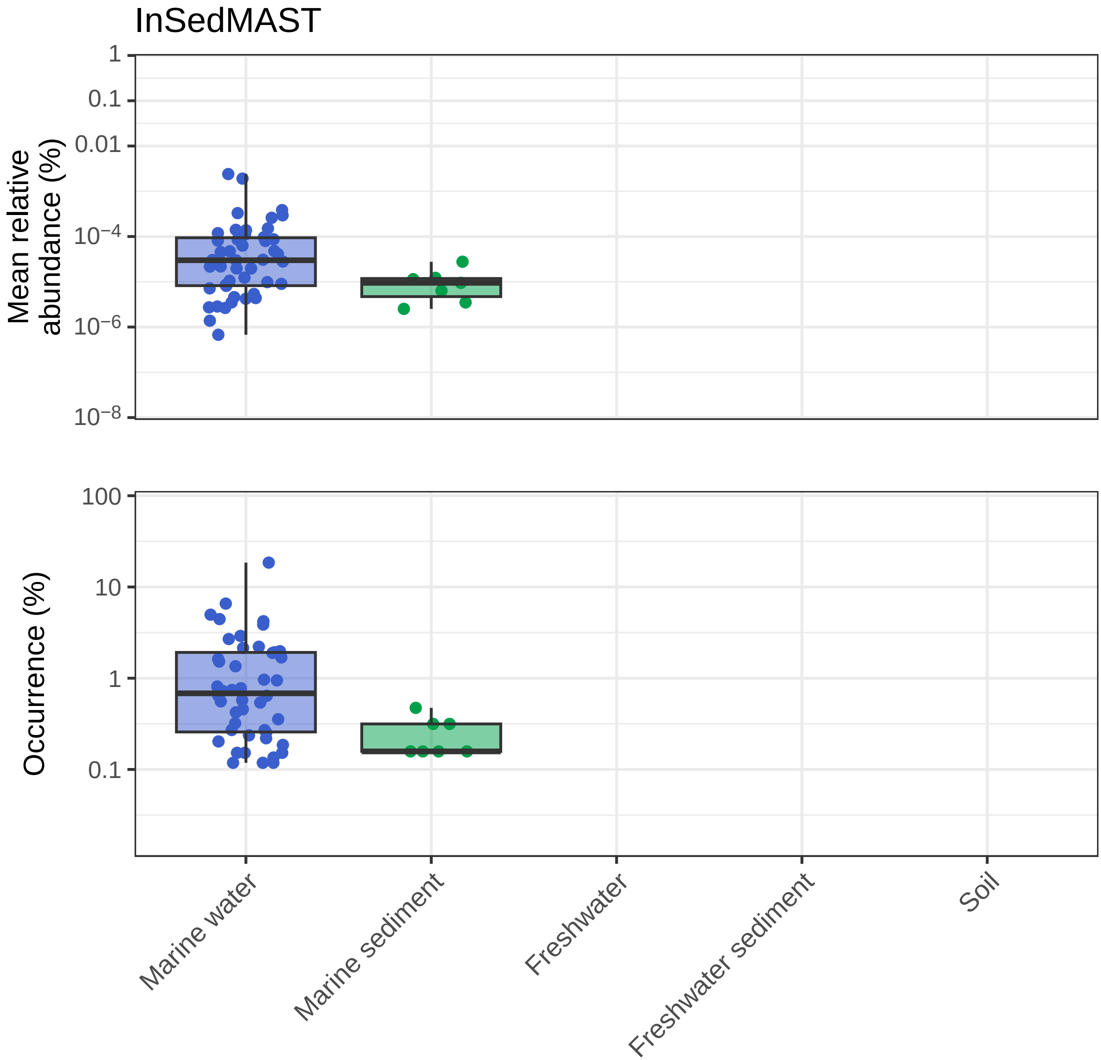

InSedMAST
Mean relative abundance (%)
1
0.1
0.01
10^{-4}
10^{-6}
10^{-8}
Occurrence (%)
100
10
1
0.1
Marine water
Marine sediment
Freshwater
Freshwater sediment
Soil
