# Supplementary figures and images for: How marine are Marine Stramenopiles (MAST)? A cross-system evaluation
Source: FEMS Microbiol Ecol. 2024 Oct 7;100(11):fiae130. doi: 10.1093/femsec/fiae130 (PMC11523054; doi:10.1093/femsec/fiae130)

**B**

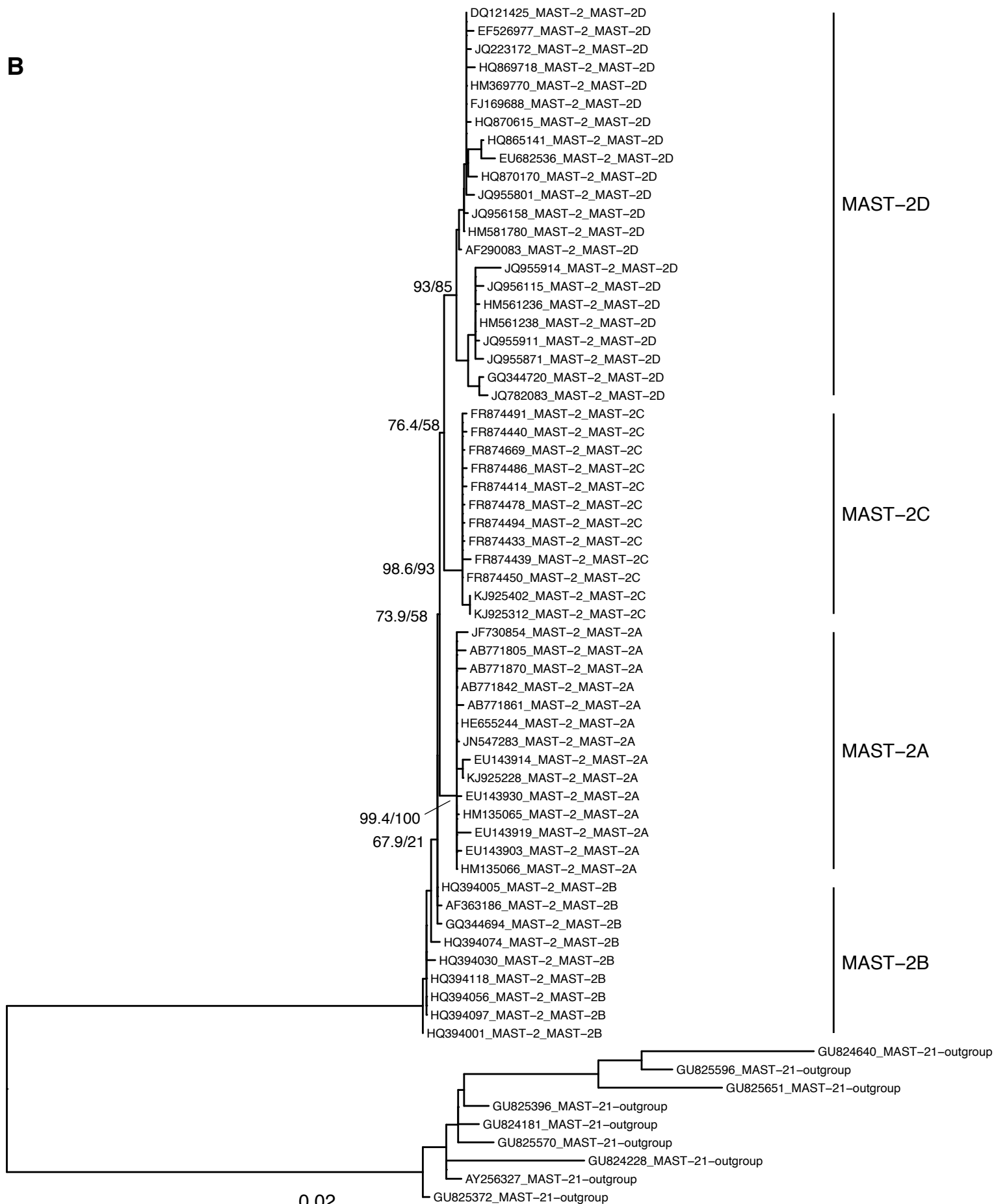

C

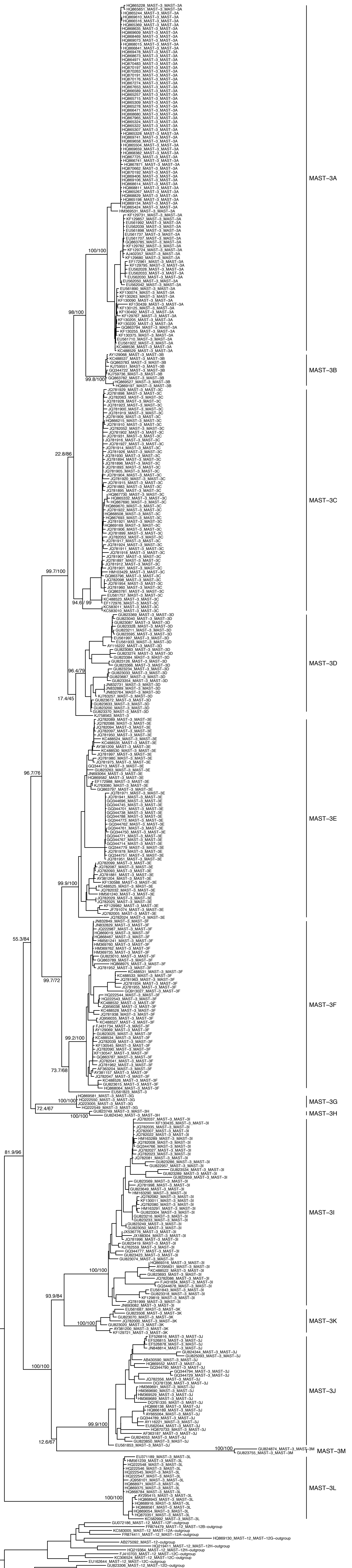



E

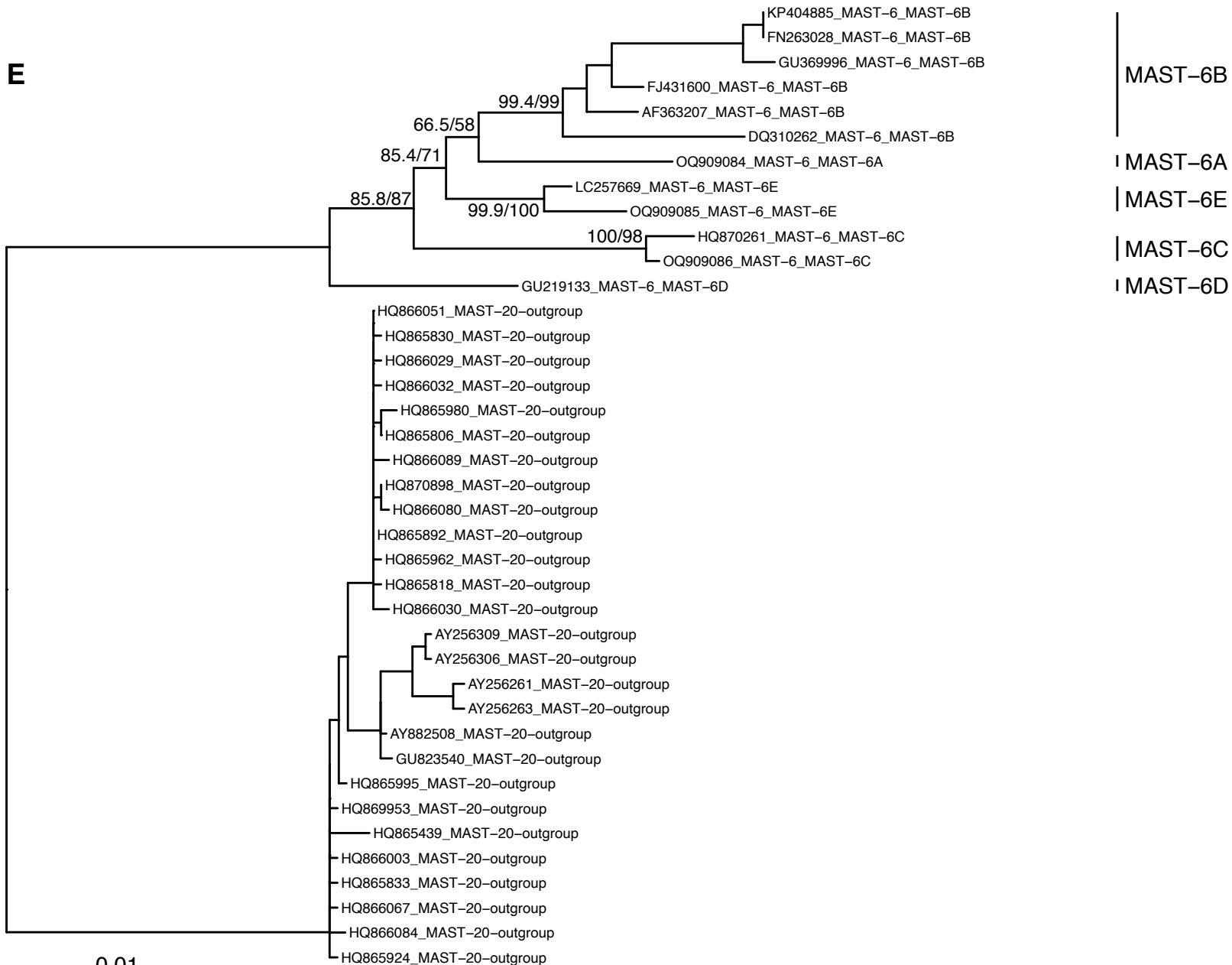

F

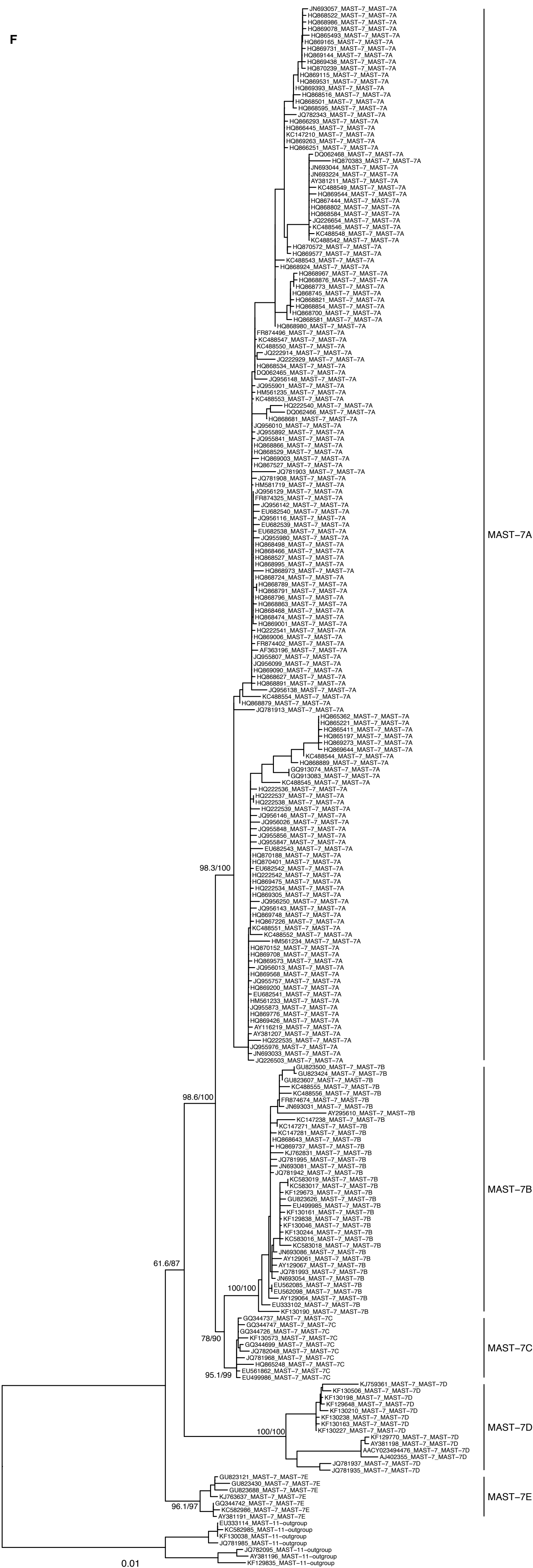

G

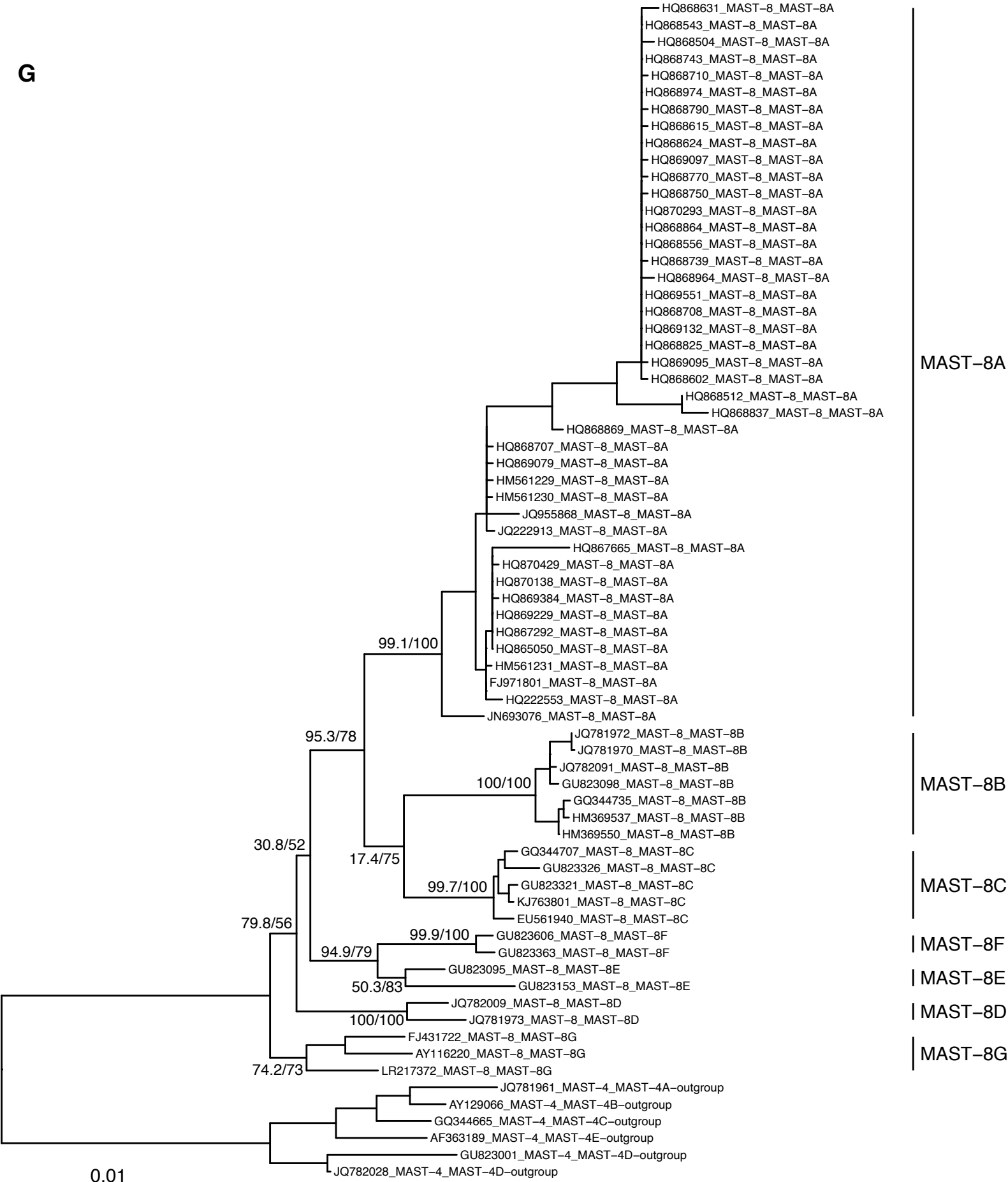

H

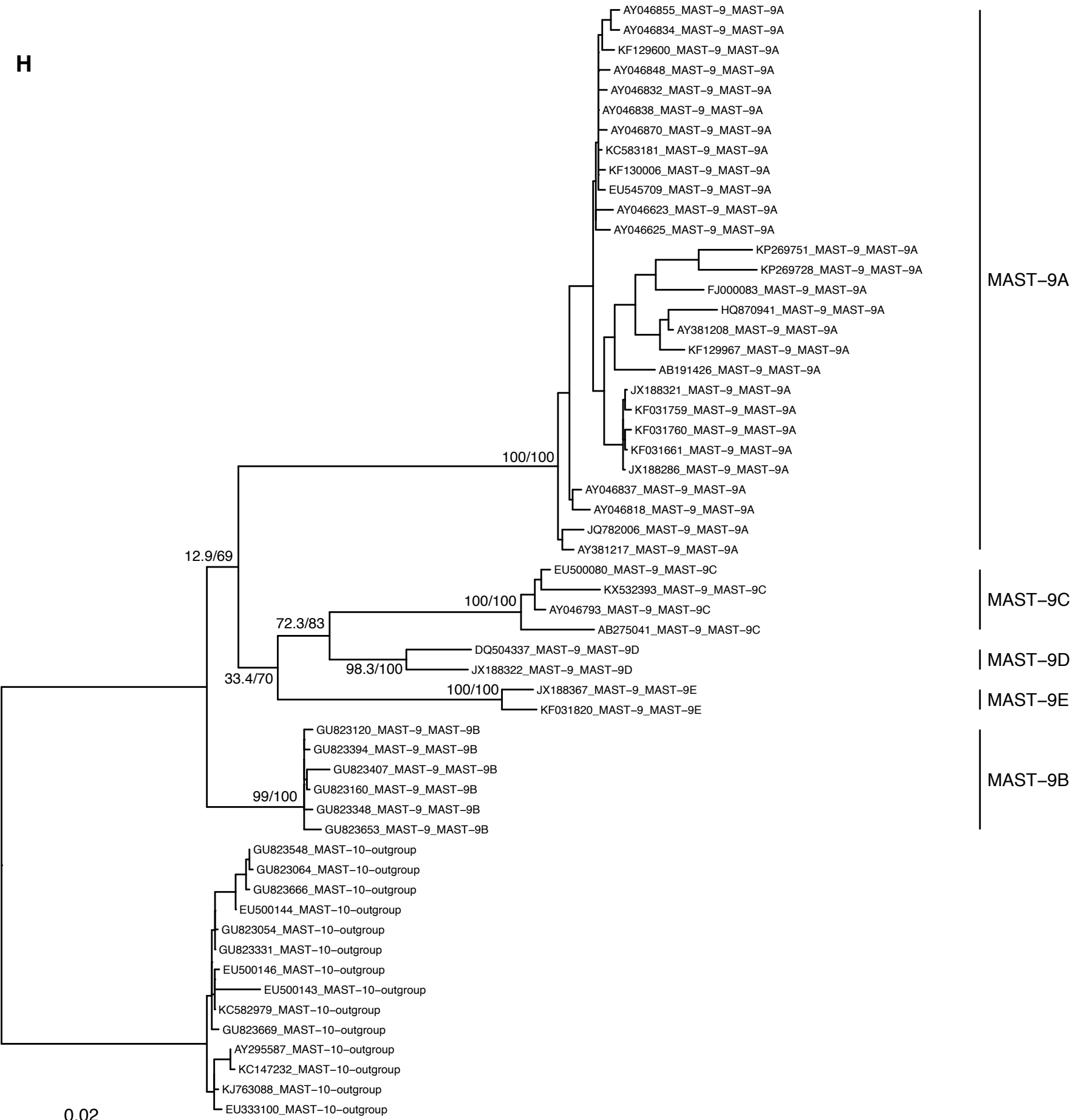

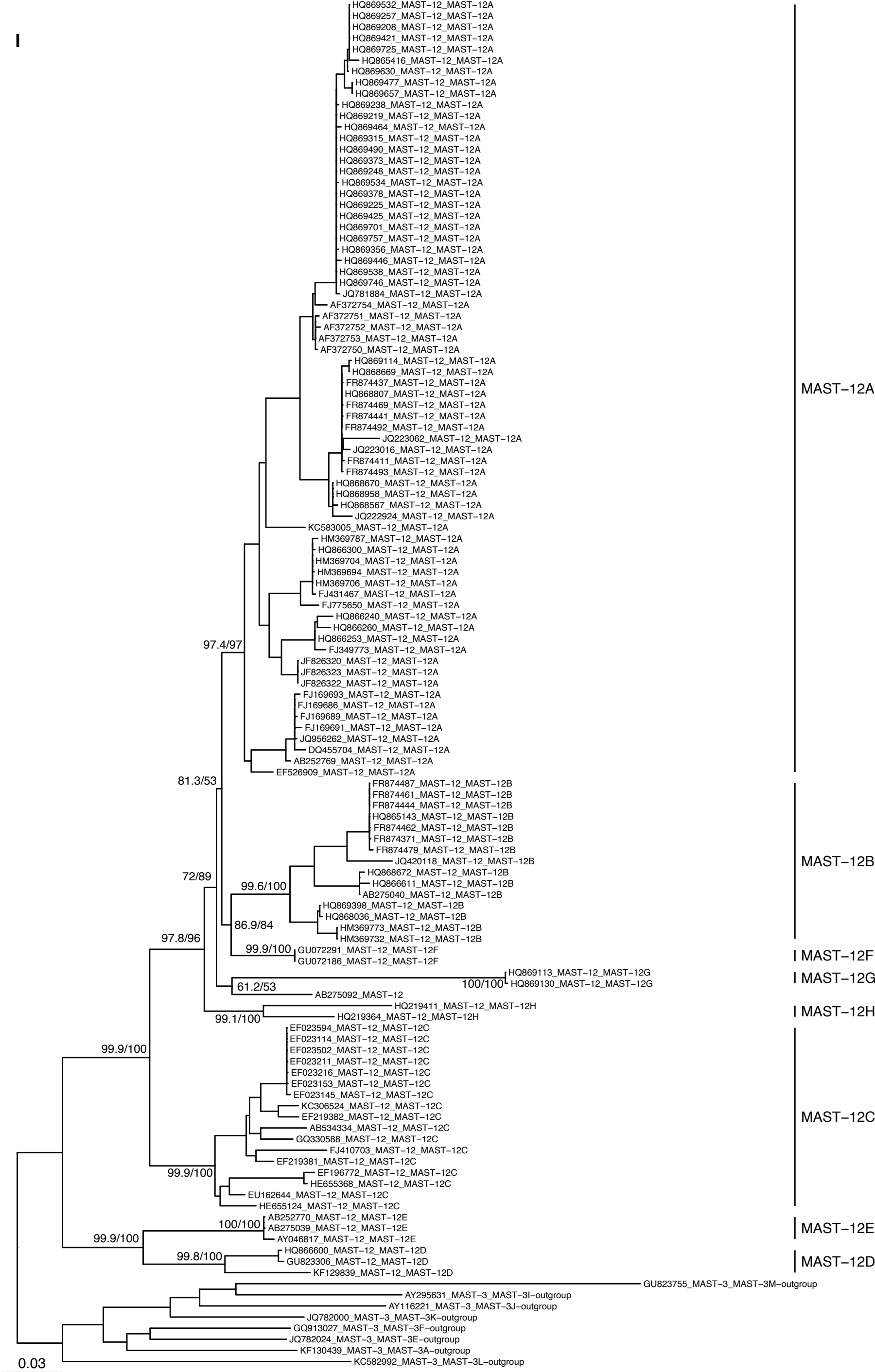

J

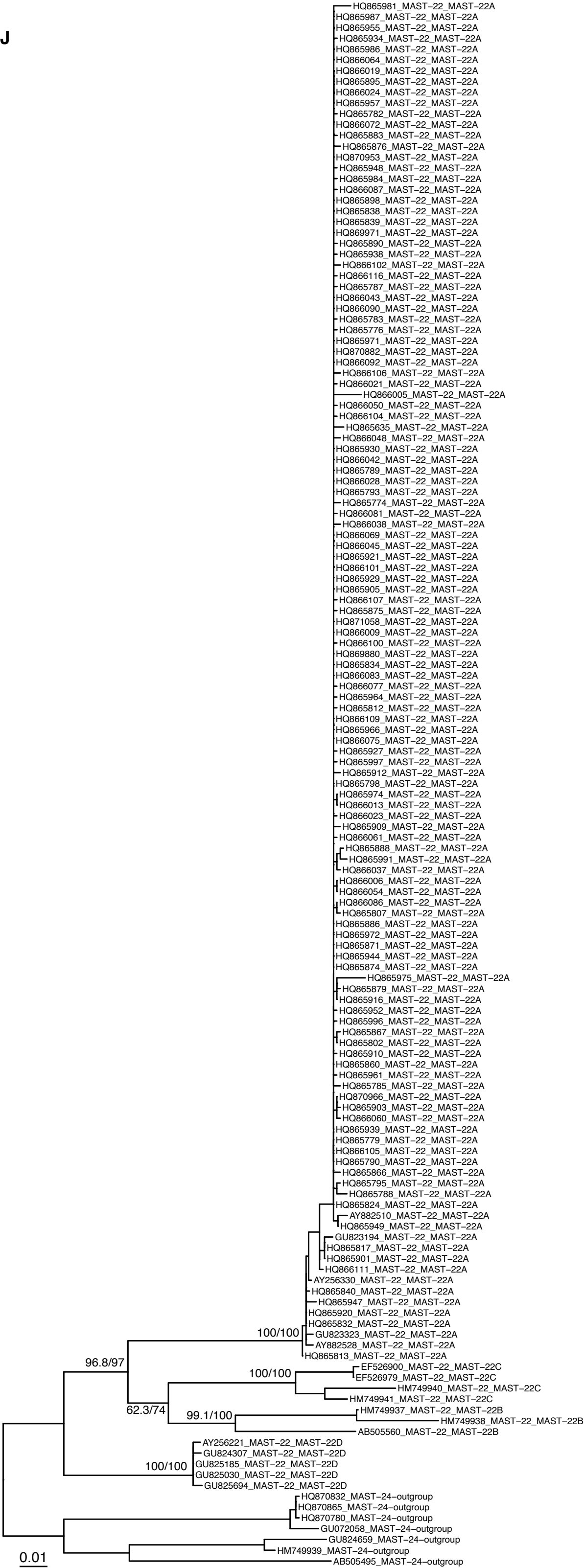

MAST-22A

MAST-22C

MAST-22B

MAST-22D

Supplement: fiae130_Supplemental_Files [file fiae130_supplemental_files.zip › figureS1.pdf]

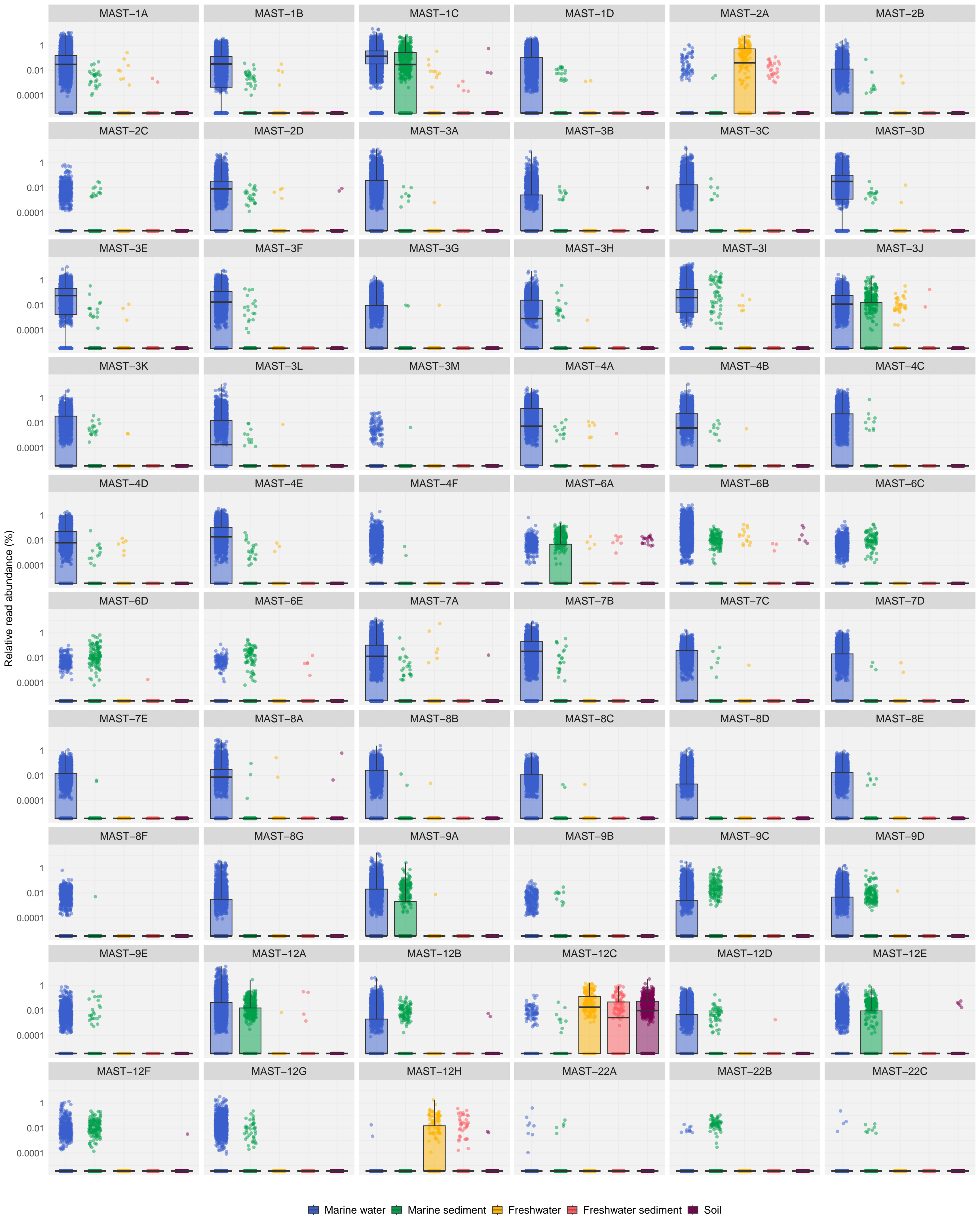

Supplement: fiae130_Supplemental_Files [file fiae130_supplemental_files.zip › figureS2.pdf]

Relative read abundance (%)

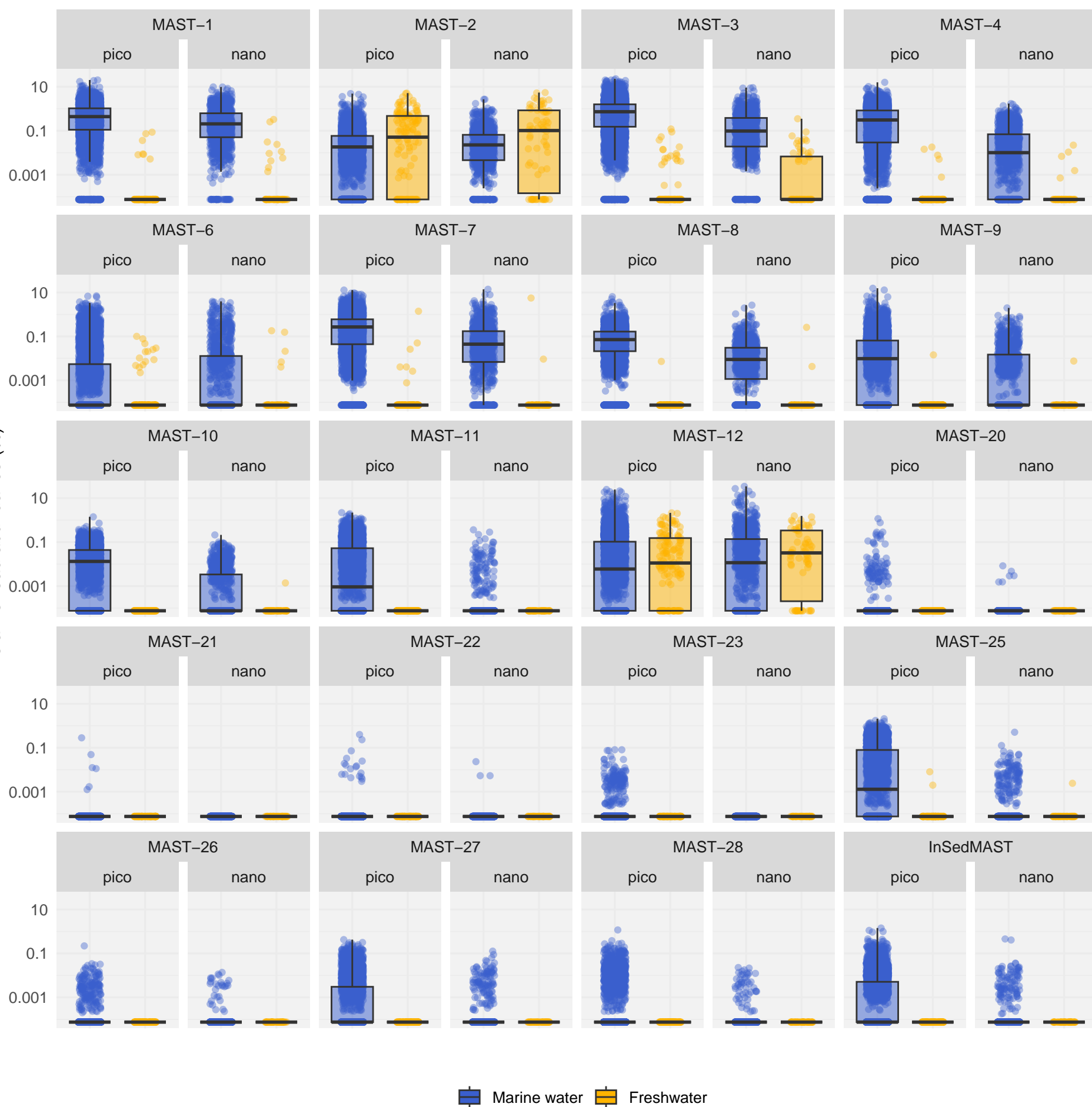

Supplement: fiae130_Supplemental_Files [file fiae130_supplemental_files.zip › figureS3.pdf]

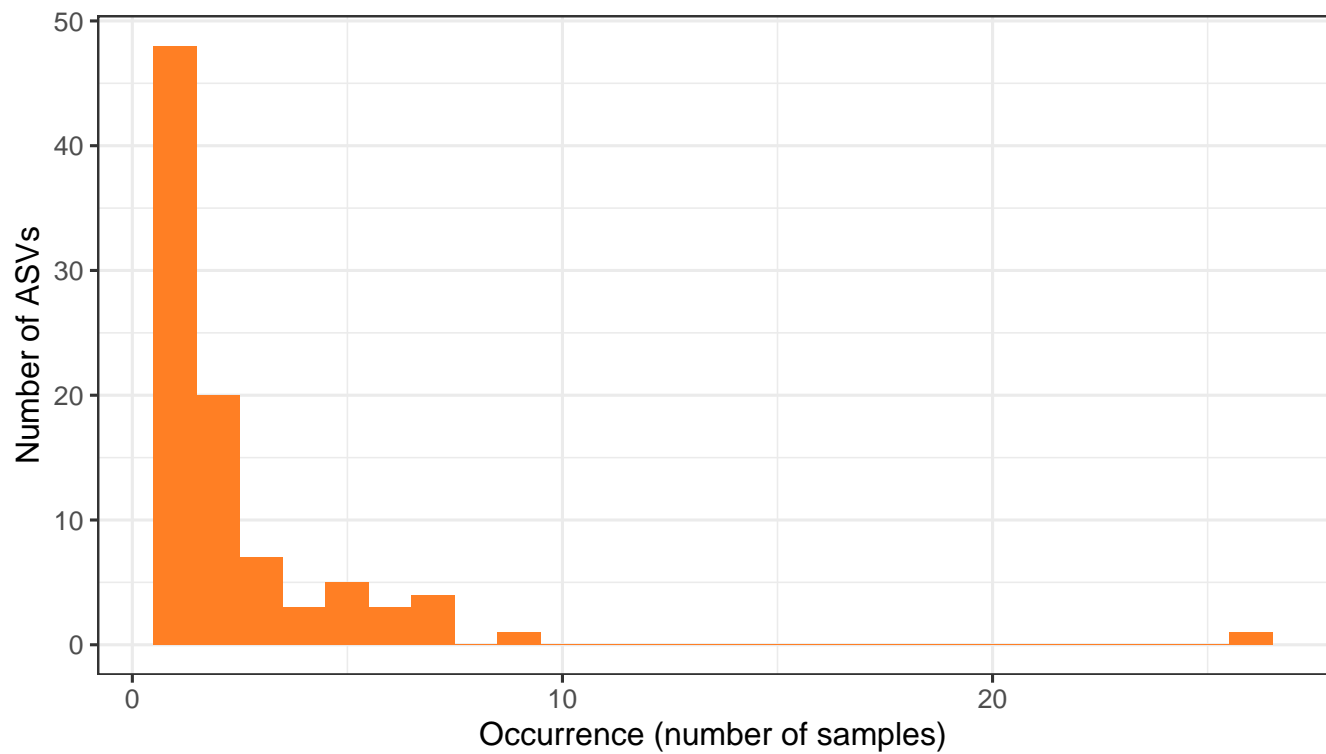

Supplement: fiae130_Supplemental_Files [file fiae130_supplemental_files.zip › figureS5.pdf]

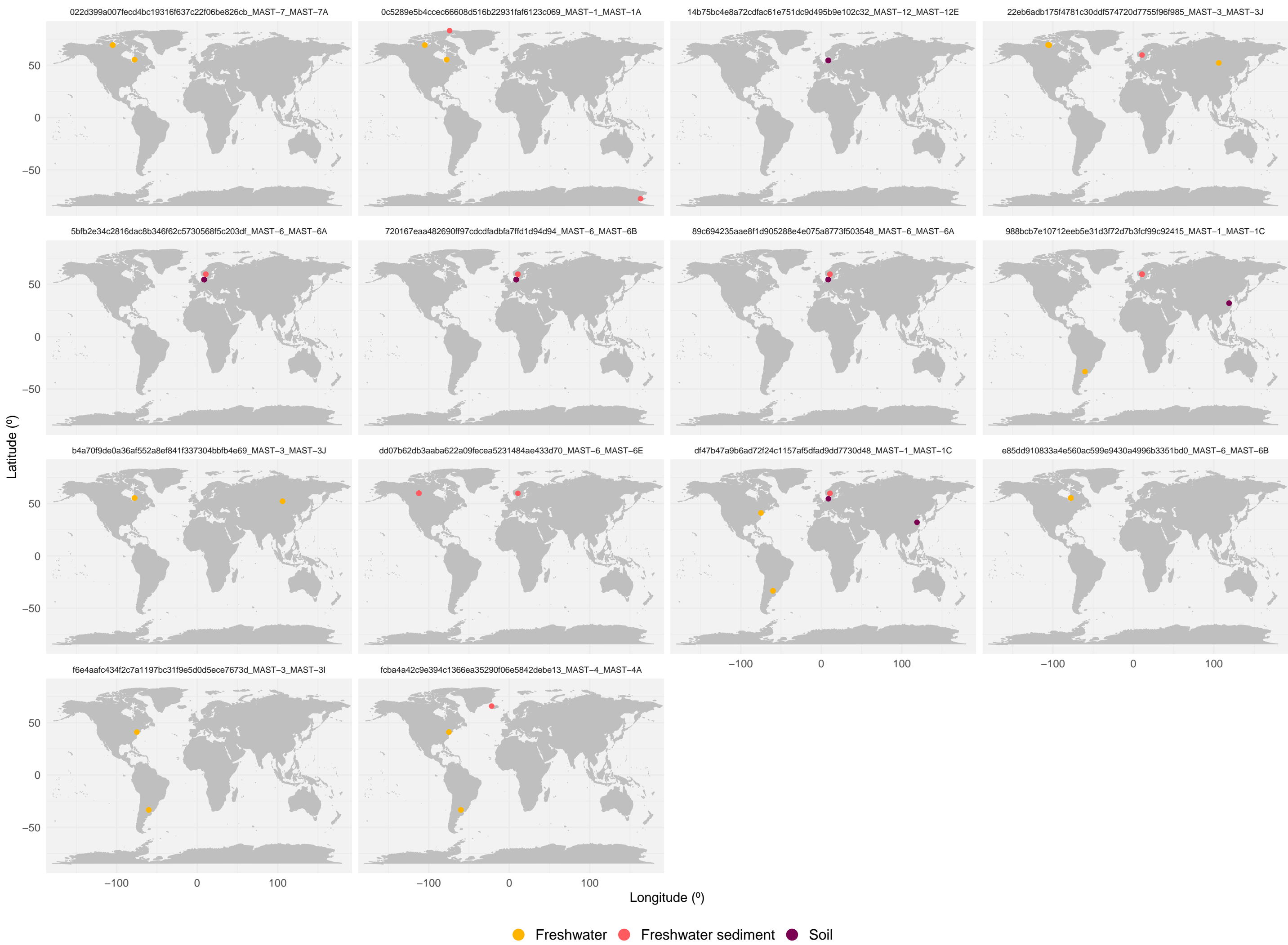

Supplement: fiae130_Supplemental_Files [file fiae130_supplemental_files.zip › figureS6.pdf]

**Marine water**

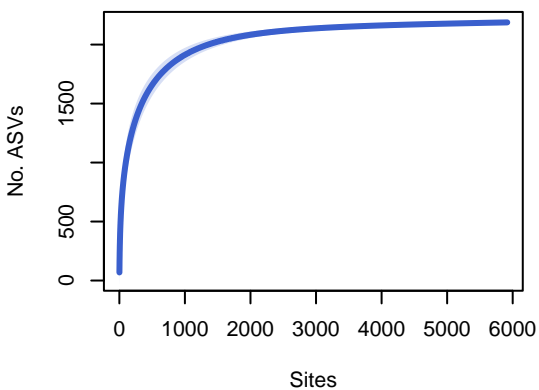

**Marine sediment**

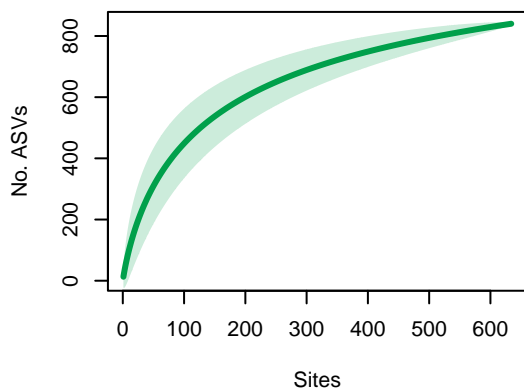

**Freshwater**

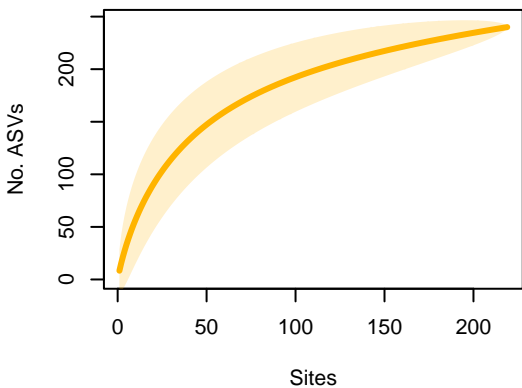

**Freshwater sediment**

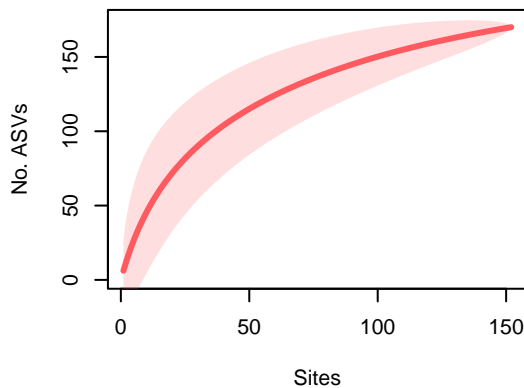

**Soil**

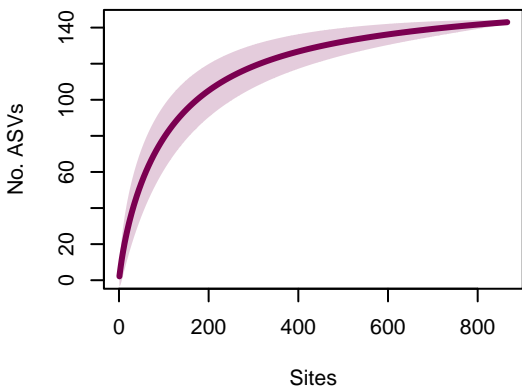

Supplement: fiae130_Supplemental_Files [file fiae130_supplemental_files.zip › figureS7.pdf]

A

## Relative read abundance

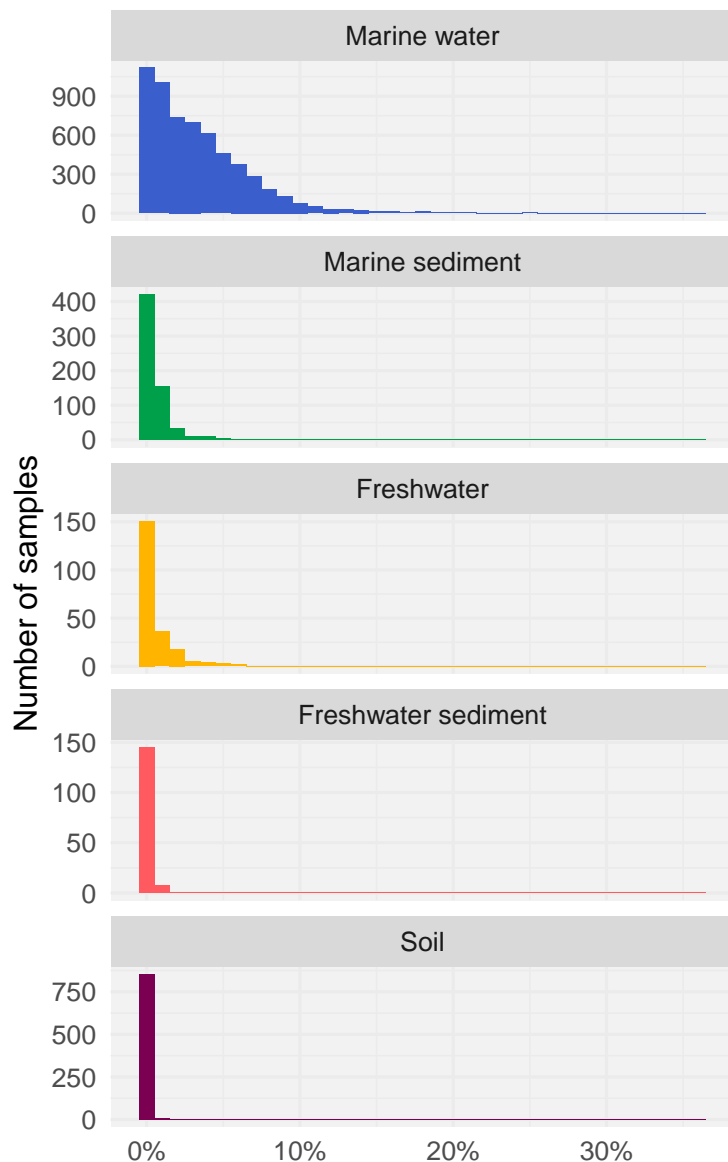

B

## Occurrence

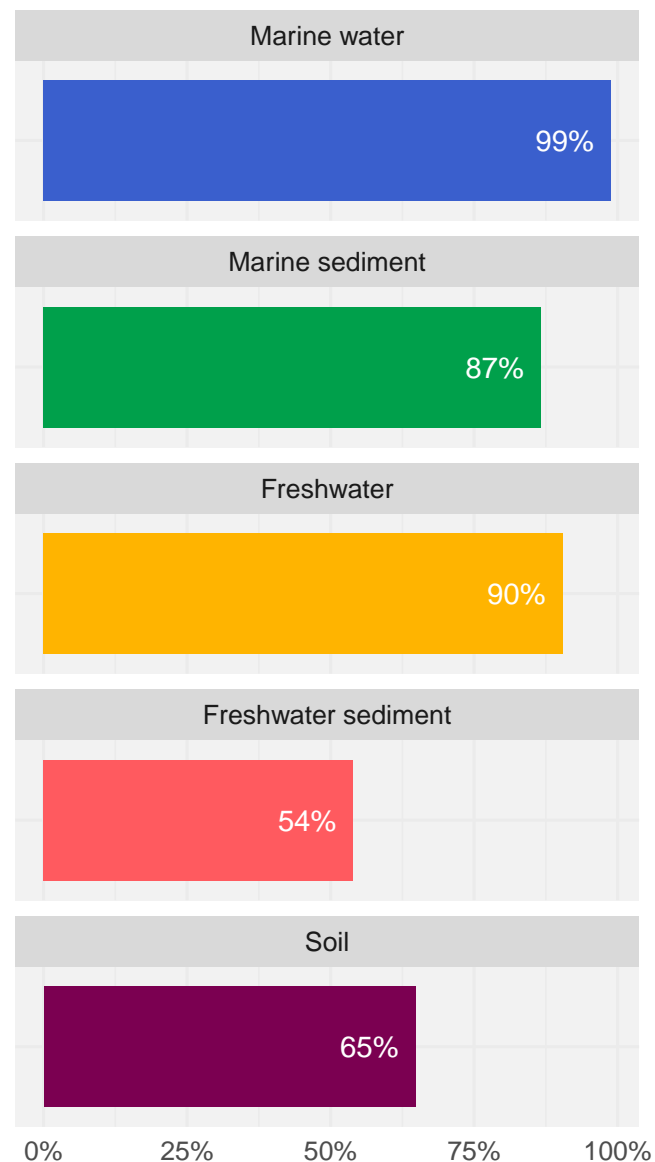

Supplement: fiae130_Supplemental_Files [file fiae130_supplemental_files.zip › figureS8.pdf]
